# A pipeline for complete characterization of complex germline rearrangements from long DNA reads

**DOI:** 10.1186/s13073-020-00762-1

**Published:** 2020-07-31

**Authors:** Satomi Mitsuhashi, Sachiko Ohori, Kazutaka Katoh, Martin C. Frith, Naomichi Matsumoto

**Affiliations:** 1 Department of Human Genetics, Yokohama City University Graduate School of Medicine, Fukuura 3-9, Kanazawa-ku, Yokohama, 236-0004 Japan; 2 Research Institute for Microbial Diseases, Osaka University, Suita, Japan; 3 Artificial Intelligence Research Center, National Institute of Advanced Industrial Science and Technology (AIST), 2-3-26 Aomi, Koto-ku, Tokyo, 135-0064 Japan; 4 Graduate School of Frontier Sciences, University of Tokyo, Kashiwa, Japan; 5 Computational Bio Big-Data Open Innovation Laboratory (CBBD-OIL), AIST, Tokyo, Japan

**Keywords:** Long read sequencing, Chromosomal translocations/rearrangements, Chromothripsis, Structural variation

## Abstract

**Background:**

Many genetic/genomic disorders are caused by genomic rearrangements. Standard methods can often characterize these variations only partly, e.g., copy number changes or breakpoints. It is important to fully understand the order and orientation of rearranged fragments, with precise breakpoints, to know the pathogenicity of the rearrangements.

**Methods:**

We performed whole-genome-coverage nanopore sequencing of long DNA reads from four patients with chromosomal translocations. We identified rearrangements relative to a reference human genome, subtracted rearrangements shared by any of 33 control individuals, and determined the order and orientation of rearranged fragments, with our newly developed analysis pipeline.

**Results:**

We describe the full characterization of complex chromosomal rearrangements, by filtering out genomic rearrangements seen in controls without the same disease, reducing the number of loci per patient from a few thousand to a few dozen. Breakpoint detection was very accurate; we usually see ~ 0 ± 1 base difference from Sanger sequencing-confirmed breakpoints. For one patient with two reciprocal chromosomal translocations, we find that the translocation points have complex rearrangements of multiple DNA fragments involving 5 chromosomes, which we could order and orient by an automatic algorithm, thereby fully reconstructing the rearrangement. A rearrangement is more than the sum of its parts: some properties, such as sequence loss, can be inferred only after reconstructing the whole rearrangement. In this patient, the rearrangements were evidently caused by shattering of the chromosomes into multiple fragments, which rejoined in a different order and orientation with loss of some fragments.

**Conclusions:**

We developed an effective analytic pipeline to find chromosomal aberration in congenital diseases by filtering benign changes, only from long read sequencing. Our algorithm for reconstruction of complex rearrangements is useful to interpret rearrangements with many breakpoints, e.g., chromothripsis. Our approach promises to fully characterize many congenital germline rearrangements, provided they do not involve poorly understood loci such as centromeric repeats.

## Background

Various germline DNA sequence changes are known to cause rare genetic disorders. Many small nucleotide-level changes (one to a few bases) in 4209 genes have been reported in OMIM (https://www.omim.org/) (as of Jan 21, 2020), which are known as single gene disorders. In addition to these small changes, large structural variations of the chromosomes can also cause diseases.

Previous studies on pathogenic structural changes in patients with genetic/genomic disorders found chromosomal abnormalities by microscopy, by detecting copy number variations (CNVs) using microarrays [1], or by detecting both CNVs and breakpoints using high-throughput short read sequencing [2]. However, there are difficulties in precisely identifying sequence-level changes especially in highly similar repetitive sequences (e.g., simple repeats, recently integrated transposable elements) or in finding how these rearrangements are ordered [3]. Long read sequencing (PacBio or nanopore) is advantageous for characterizing rearrangements in such cases and is recently beginning to be used for patient genome analysis to identify pathogenic variations [4–6]. In addition, if rearrangements are complex (e.g., chromothripsis), long read sequencing (reads often exceed 10 kb in length) has a further advantage, because one read may encompass all or much of a complex rearrangement [7]. Chromothripsis is a chaotic complex rearrangement, where many fragments of the genome are rearranged into derivative chromosomes. Current approaches to analyze chromothripsis usually require manual inspection to reconstruct whole rearrangements. Detection and reconstruction methods for complex rearrangements are needed to characterize pathogenic variations from whole genome sequencing data.

**Fig. 1:**
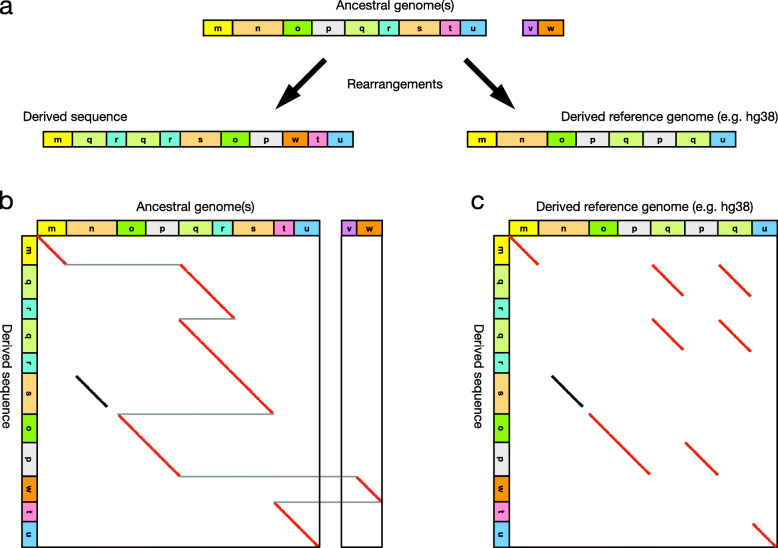
Illustration of genome evolution with rearrangements. **a** Starting from ancestral DNA, evolution with rearrangements results in derived sequences with deletions, duplications, and re-ordered fragments. Each colored block represents a piece of a chromosome, e.g., a few thousand basepairs. The blocks labeled n and s are similar repeated sequences (same color). **b** Comparison of derived sequence (vertical) to ancestral sequence (horizontal). The diagonal red lines show which ancestral basepair each basepair in the derived sequence is descended from: starting at a derived basepair, go horizontally to the right until hitting a red line, then go up vertically to find the ancestral basepair. The diagonal black line indicates a misleading (paralogous) similarity between the ancestral and derived sequences. **c** Comparison of the same derived sequence (vertical) to a derived reference genome (horizontal). Diagonal red lines show basepairs in the horizontal and vertical sequences that are descended from the same basepair in the most recent common ancestor of the sequences. The diagonal black line shows similar segments that are not descended from the same part of the most recent common ancestor

Rearrangements arise in various ways such as gene conversion, processed pseudogene integration, aberrant DNA replication with template switching [8, 9], and probably as-yet unknown mechanisms. Regardless, the result is duplicated, deleted, re-ordered and/or re-oriented fragments (Fig. 1a). No matter how complex the rearrangement, there is a simple relationship between ancestral and derived sequences: every part of the derived sequence comes from a unique part of the ancestor [10]. (The unusual exception is “spontaneously generated” sequence not descended from an ancestor, a.k.a. non-templated insertion: we allow for it by allowing parts of the derived sequence to not align anywhere.) Thus, a rearrangement can be displayed as in Fig. 1b: the derived sequence is shown vertically, and we can see from top to bottom where each part came from (red diagonal lines).

Unfortunately, we do not have an ancestral genome sequence (further discussed in Additional file 1). The reference genome has its own rearrangements: this makes it qualitatively harder to identify segments descended from the same segment in the most recent common ancestor of the genomes (red diagonal lines in Fig. 1c). Even if we could identify them, the result is hard to understand. To make the problem tractable, we assume the reference is ancestral: though false, this works well enough to be useful.

**Fig. 2:**
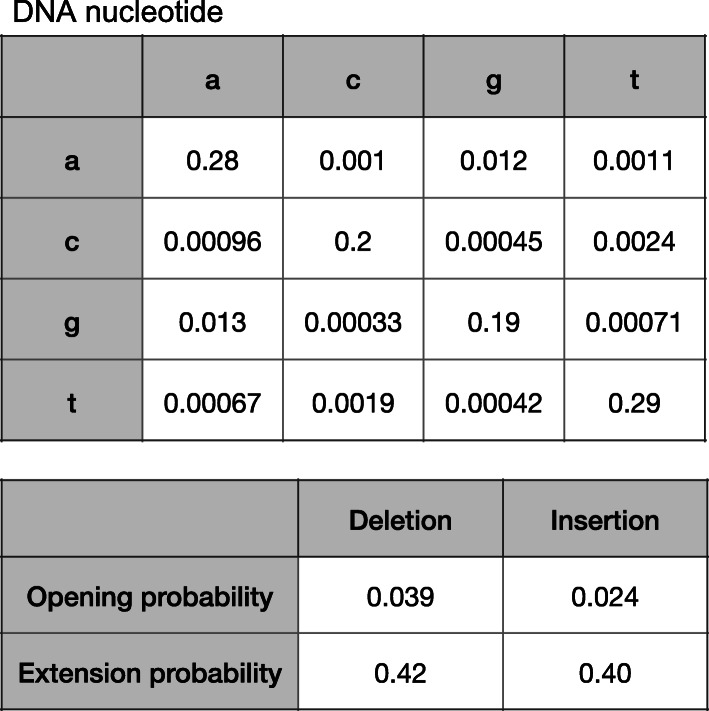
Rates (probabilities) of substitutions, deletions, and insertions between a set of nanopore human DNA reads and reference genome hg38. The 4 × 4 matrix shows substitution probabilities: rows correspond to genome bases and columns correspond to read bases. The rates in Fig. 2 are a combination of sequencing errors and real differences

Concretely, we compare long DNA reads to an assumed-ancestral reference genome, by inferring which part of the reference each part of the read comes from. Thus, we need to accurately divide the read into (one or more) parts and align each part to the genome. To do this, we first learn the rates of small insertions, deletions, and each kind of nucleotide substitution between reads and genome (e.g., Fig. 2) [11], then find the most-likely division and alignment based on these rates [10, 12]. We can also calculate the probability that each base is wrongly aligned, which is high when part of a read aligns almost equally well to several genome loci. This approach was previously used to characterize rearrangements that are “localized,” i.e., encompassed by one DNA read [10].

Here we extend this approach, to find arbitrary (non-localized) rearrangements, subtract rearrangements found in control individuals, then order and orient rearranged DNA reads to fully reconstruct complex rearrangements in derivative chromosomes. To the best of our knowledge, there is no other tool to fully reconstruct complex rearrangements from only long reads and filter out benign changes. Chromothripsis has been analyzed by NanoSV [7], but its website states: “we decided to call only breakpoints instead of SV types (such as inversions, deletions, etc.).” Indeed, it is hard to tell whether (e.g.) a split alignment of a DNA read to both strands of a chromosome indicates a simple inversion, or part of a more complex rearrangement (see examples below).

Recently, long read sequencing was used to detect structural variants in human genomes, but focusing on simple insertions, deletions, and inversions [13]. However, another study used linked-read sequencing to document more complex types of rearrangement such as del-INV-del and del-INV-dup [14]. There have been several approaches to characterize pathogenic complex rearrangements in congenital diseases [7, 15, 16]. Beck et al. used long reads to detect chr17p11.2 recurrent rearrangement using a targeted approach [15]. Targeted approaches are limited and hard to use for complex chromothripsis. Eisfeldt et al. analyzed three patients with complex chromosomal rearrangement (CCR) [16]. This approach required several different methods to fully understand the CCR (including short read sequencing, optical mapping, and linked-read sequencing). For clinical application, a single method that can characterize complex rearrangements would be useful. Our approach can characterize pathogenic rearrangements using only long read whole genome sequencing and thus should be useful for further clinical applications.

Moreover, we show that complex rearrangements can have emergent properties, such as deletions, that are knowable only after fully reconstructing the whole rearrangement. Finally, we believe our pipeline for long DNA reads is unique in discarding rearrangements shared by other genomes (controls), which is critical for practical utility, because human genomes typically differ by thousands of, presumably benign, large-scale rearrangements.

## Methods

### Patients

We studied 4 patients whose breakpoints were previously not fully detected by high-throughput analysis, among 9 patients with chromosomal abnormalities [17]. Patients 1 and 2 have primary ovarian failure (detailed clinical information in Additional file 1 and elsewhere [18, 19]). Patient 3 has split-hand-foot malformation (detailed information was published elsewhere [20]). Patient 4 has intractable epilepsy and is suspected to have a chromosomal translocation breakpoint in centromeric repeats [21].

### Controls

We used 33 human controls to filter out benign rearrangements in the patients. Because genome-wide long read sequencing remains expensive, we re-used data from previous studies [6]. Thus, many of these controls have genetic disorders (Additional file 1: Table S1), which are unlikely to be related to those of the 4 patients.

### Nanopore sequencing using PromethION

DNA was extracted from patients’ blood cells. Libraries were prepared for nanopore sequencing using DNA ligation kit (SQK-LSK109) then subjected to PromethION sequencing (Oxford Nanopore Technologies) using one PRO-002 (R9.4.1) flowcell according to the manufacturer’s protocol. Base-calling and fastq conversion were performed with MinKNOW ver1.14.2. Control datasets were also sequenced by PromethION. Base-calling and fastq conversion were performed with MinKNOW ver1.11.5. The genome sequencing coverage ranged from 12x to 42x (Additional file 1: Table S1).

### Data analysis

Our task is to find and fully characterize rearrangements in a patient’s genome that are absent in control genomes. By “fully characterize,” we mean to determine which part of the reference genome each part of the rearranged sequence comes from and determine the order and orientation of these parts. We do so by these steps (details in Additional file 1: Supplementary Methods and Fig. S1–4), using software named dnarrange that was developed for this study.

1. Align the DNA reads to the reference genome, by probability-based split alignment. This gives us rearranged reads, but there are two difficulties:
  (i) There seem to be many artifactually rearranged reads, at least in some datasets [10]. Some putative artifacts are shown in Additional file 1: Fig. S3. These artifacts seem to be mostly sporadic [10], so they can be excluded by requiring at least 2 or 3 reads to cover the same rearrangement.
  (ii) It is hard to tell whether a rearranged read covers a whole rearrangement, or part of a larger rearrangement, or multiple independent rearrangements. We defer making this judgment, and eventually do so manually.
2. Discard any patient read that has any two rearranged fragments in common with any control read. Ideally, we would discard whole rearrangements rather than reads, but whole rearrangements have not been determined yet due to difficulty (ii).
3. Discard any patient read that has any rearrangement not shared by any other read from the same patient. This aims to remove artifacts.
4. Group reads from one patient that cover the same rearrangement (i.e., have two rearranged fragments in common). Discard groups with fewer than 3 reads: this also aims to remove artifacts.

In the following results, we at first omit step 2 to show the results without control filtering, then re-run steps 2–4 to show the results with filtering. Steps 2–4 can be done with one simple “dnarrange” command:

dnarrange patient-file : control1 control2 ... > groups

5. Examine dotplots showing how each read group aligns to the reference genome. Manual examination is feasible because the number of groups, after filtering, is typically a few dozen. In practice, we can often tell that a group of reads covers a whole rearrangement of a specific type, e.g., integration of a processed pseudogene, transposable element, or NUMT (nuclear mitochondrial DNA). Other read groups are suspected to cover parts of larger rearrangements.
6. Merge each group of reads into a more accurate consensus sequence, using lamassemble [22], and re-align these consensus sequences to the genome. This step has a chance of characterizing rearranged fragments more accurately, but in practice, it rarely changes the picture and is not critical. In previous work, such consensus sequences were important for revealing the sequences of tandem repeat expansions [6].
7. Infer the order and orientation of read groups that are suspected to cover parts of a larger rearrangement. This is done by a parsimony argument: we find an order and orientation that links the groups into a minimal number of rearranged chromosomes. We could always suggest a trivial solution where the genome is highly aneuploid and each read group is on a separate chromosome, but that is not parsimonious and does not match the patient karyotypes determined by microscopy. There could be more than one most-parsimonious solution (in which case we fail at full characterization), but sometimes it is unique.

### Sanger sequence confirmation of breakpoints

PCR primers for breakpoints estimated from rearrangements were designed using primer3 plus software (Additional file 1: Table S2). PCR amplification was done using ExTaq, PrimeSTAR GXL, and LATaq (Takara), then amplified products were Sanger sequenced using BioDye Terminator v3.1 Cycle Sequencing kit with 3130xl genetic analyzer (Applied Biosystems, CA, USA).

## Results

### Nanopore sequencing of 4 patients with chromosomal translocations

**Fig. 3:**
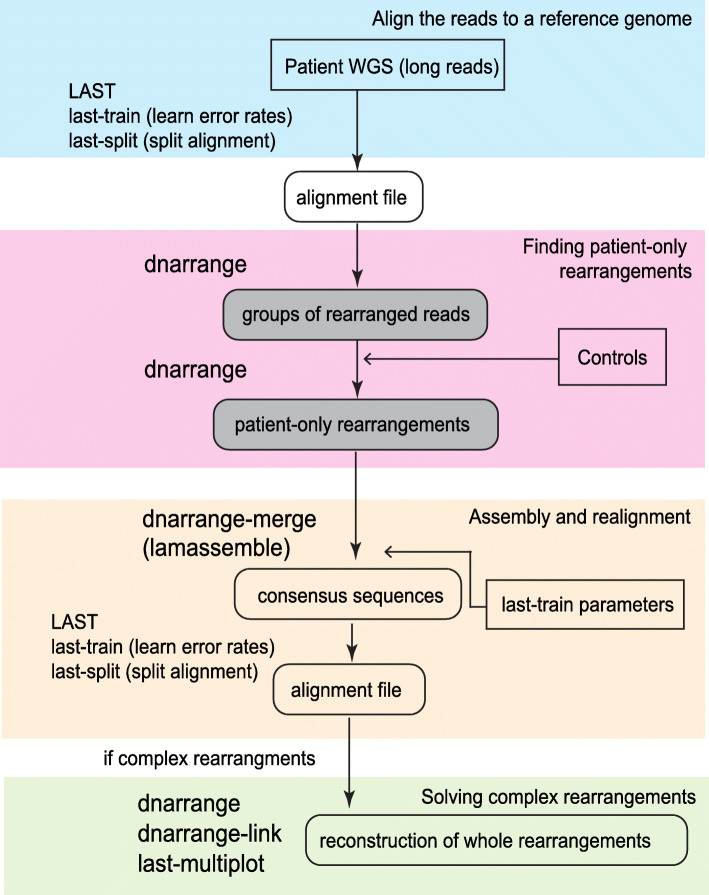
Schematic diagram of chromosomal rearrangement analysis pipeline. Long DNA reads are aligned to a reference genome using LAST (blue box), then dnarrange finds rearranged reads and groups reads that overlap the same rearrangement (pink box). lamassemble merges/assembles each group of reads into a consensus sequence (yellow box). When there is a “complex” rearrangement (more than one group of rearranged reads is needed to understand the full structure of the rearrangement), dnarrange-link was used to infer the order and orientation of the groups and thereby reconstruct derivative chromosomes (green box)

**Fig. 4:**
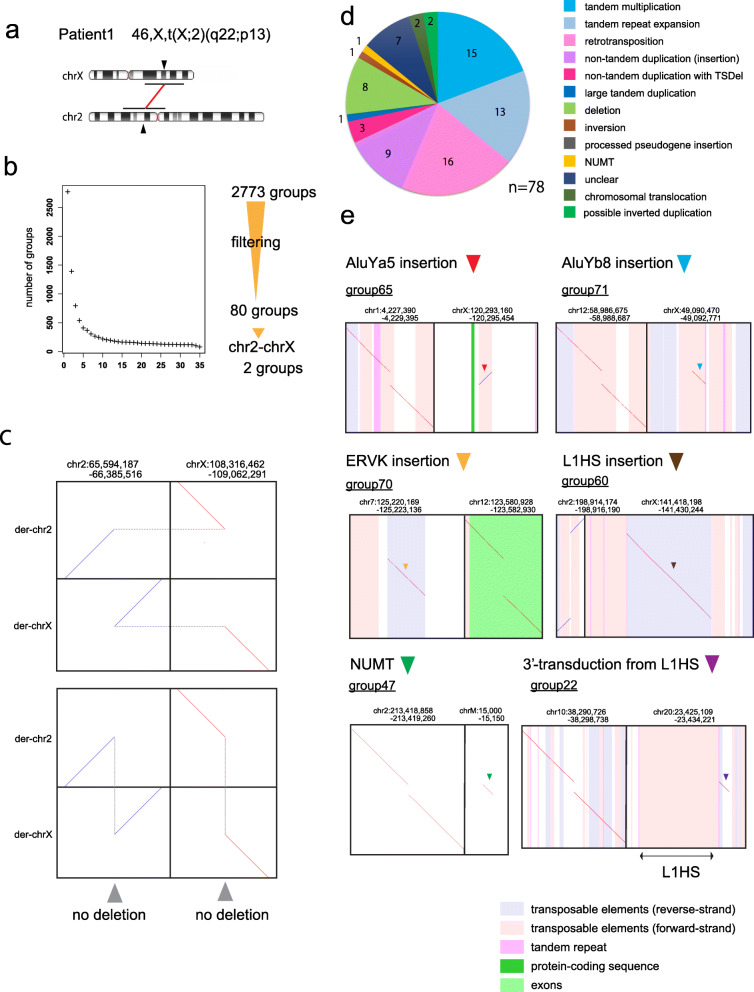
Chromosomal rearrangement in patient 1 with 46,X,t(X;2)(q22;p13). **a** Ideograms showing patient 1’s translocation between chrXq22 and chr2p13. Chromosome images are from NCBI genome decoration (https://www.ncbi.nlm.nih.gov/genome/tools/gdp). **b** Filtering out rearrangements shared with 33 controls. Finally, 80 groups of reads with patient-only rearrangements are found. Two of the 80 groups show reciprocal chr2-chrX translocation. **c** Dotplot of reconstructed derivative chromosomes shows reciprocal balanced chromosomal translocation (upper panel: horizontal dotted gray lines join the parts of each derivative chromosome; lower panel: vertical dotted gray lines join fragments that come from adjacent parts of the reference genome, showing there is no large deletion or duplication). **d** Pie chart of the types of rearrangement. TSDel target site deletion, NUMT nuclear mitochondrial DNA insertion. **e** Examples of retrotransposition and NUMT insertion (the alignments to retrotransposons, e.g., the AluYa5 in chrX, often have low confidence, indicating uncertainty that this specific AluYa5 is the source)

**Fig. 5:**
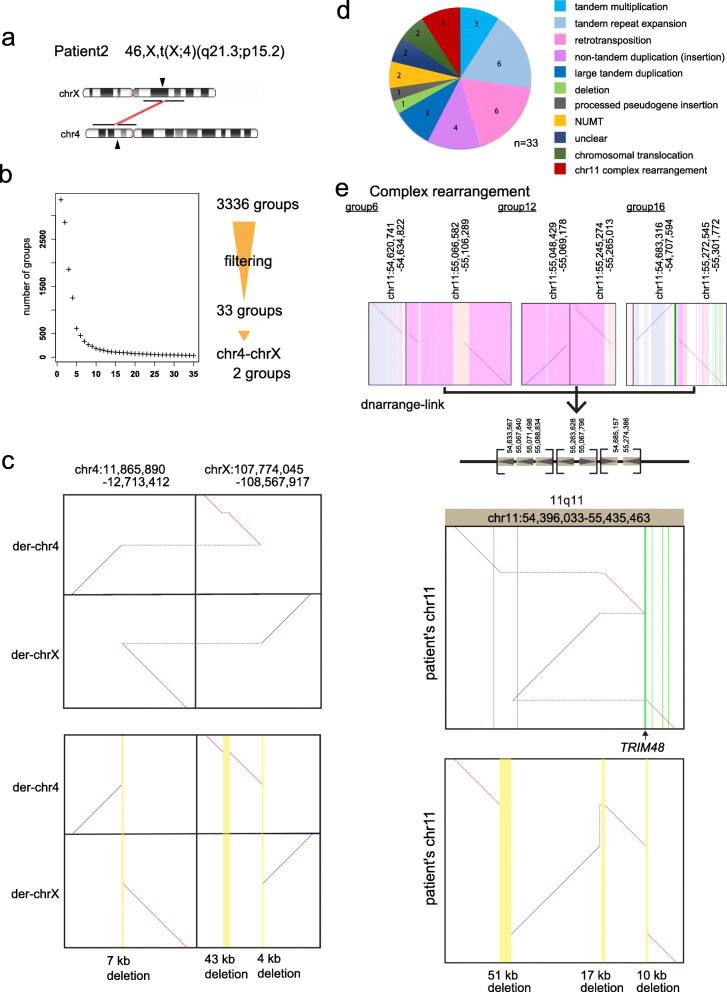
Chromosomal rearrangement in patient 2 with 46,X,t(X;4)(q21.3;p15.2). **a** Ideograms showing patient 2’s translocation between chr4p.15.2 and chrXq21.3. **b** Filtering out rearrangements shared with controls produces 33 groups of reads with patient-only rearrangements. Two of the 33 groups show chr4-chrX translocation. **c**. Dotplot of derivative (vertical) versus ancestral/reference (horizontal) chromosomes showing reciprocal chromosomal translocation. There are 7-kb and 4-kb deletions at the breakpoints in chr4 and chrX, respectively. There is also a 43-kb deletion in chrX. Yellow vertical lines show deletions. **d** Pie chart of patient-only rearrangements. **e** A complex rearrangement on chr11. Three dotplots at chr11q11 were linked to reconstruct a complex rearrangement with three sequence losses, chr11:54633567-54685157 (51 kb), chr11:55071498-55088834 (17 kb), and chr11:55263629-55274386 (10 kb). The latter disrupts the *TRIM48* gene, which is not known to cause any diseases. Upper dotplot panel: horizontal dotted gray lines join the parts of each derivative chromosome. Green vertical lines show exons. Lower dotplot panel: vertical dotted gray lines join fragments that come from adjacent parts of the reference genome. Yellow vertical lines show deletions

**Fig. 6:**
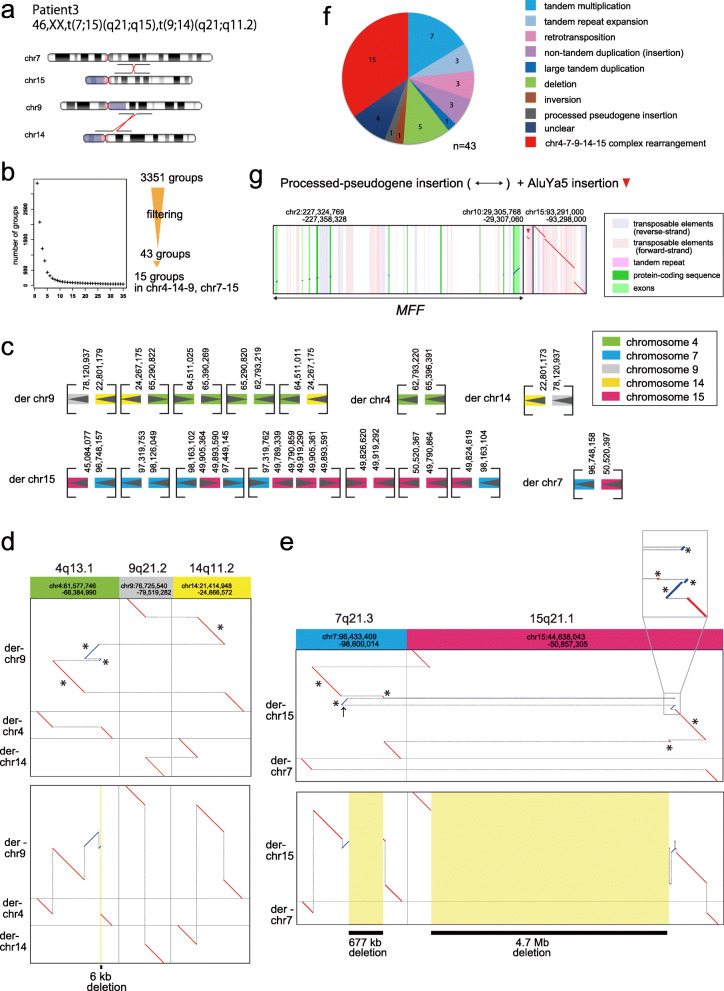
Chromosomal rearrangement in patient 3 with 46,XX,t(7;15)(q21;q15),t(9;14)(q21;q11.2). **a** Ideograms showing translocation positions of patient 3. **b** Filtering out rearrangements shared with 33 controls produces 43 groups of reads with patient-only rearrangements. While there are 2 groups indicating chr9-chr14 reciprocal translocation, there are 8 groups involved in the chr7-chr15 translocation. **c**dnarrange-link with 5 additional groups involving chr14q11.2, 15 groups in total, which were linked to construct 5 derivative chromosomes. **d** Dotplot of reconstructed derivative chr9 and chr14 shows reciprocal balanced chromosomal translocation. Upper panel: horizontal dotted gray lines join the parts of each derivative chromosome. Asterisks indicate fragments. Lower panel: vertical dotted gray lines join fragments that come from adjacent parts of the reference genome, showing there is a 6-kb deletion on chr4. **e** Dotplot of joined fragments showing reciprocal chr7-chr15 translocation with complex rearrangements. Black arrow indicates inverted 129-kb region that caused misinterpretation of deletion size. Upper panel: horizontal gray lines join the parts of each derivative chromosome. Asterisks indicate fragments. An inset magnifies 4 tiny fragments. Lower panel: vertical gray lines join fragments that come from adjacent parts of the reference genome, showing loss of 677 kb and 4.7 Mb of chr7 and chr15, respectively. **f** Pie chart of patient-only rearrangements. **g** Processed pseudogene insertion in chr15 from exons of *MFF* on chr2, with nearby AluYa5 insertion

**Fig. 7:**
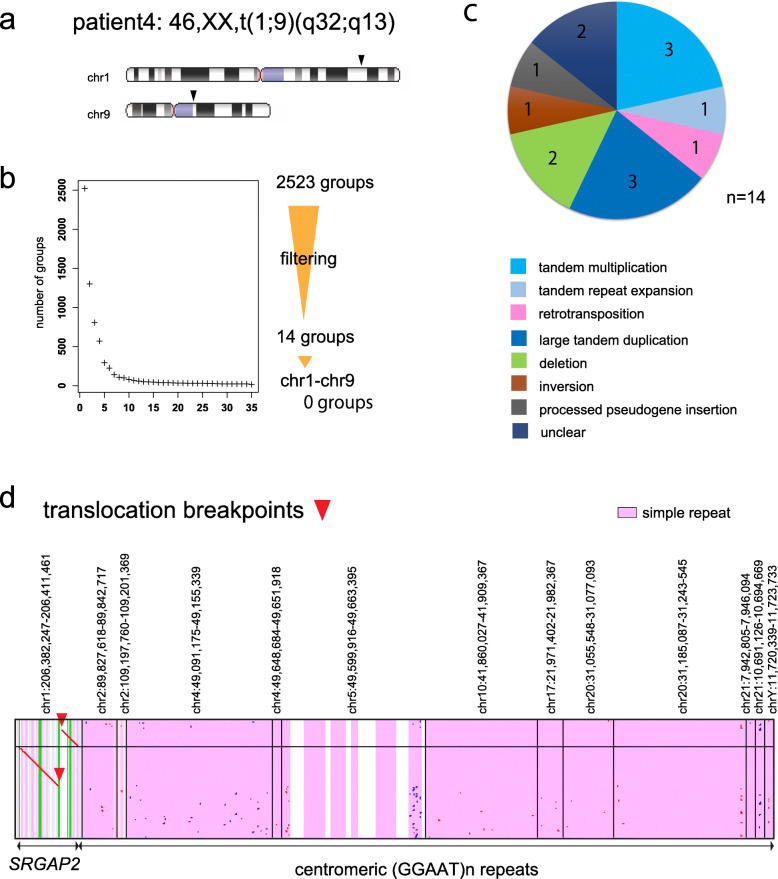
Chromosomal rearrangement in patient 4 with 46,XX, t(1;9)(q32;q13). **a** Ideograms showing translocation position of patient 4. **b** Filtering out rearrangements shared with controls produces 14 groups of reads with patient-only rearrangements. There is no group supporting chr1-chr9 translocation. **c** Pie chart of patient-only rearrangements. **d** Dotplot of two reads that cross the chr1 breakpoint

We sequenced genomic DNA from 4 patients with reciprocal chromosomal translocations using a nanopore long read sequencer, PromethION (Additional file 1: Table S1). We applied newly developed software, dnarrange (https://github.com/mcfrith/dnarrange), to find and characterize DNA sequence rearrangements in these patients. dnarrange finds DNA reads that have rearrangements relative to a reference genome, and groups reads that overlap the same rearrangement (Additional file 1: Supplementary Methods). It also filtered out rearrangements that are seen in any of 33 control individuals (Fig. 3, Additional file 1: Table S1). The number of read groups decreased exponentially with the first several controls, then stabilized, which suggests that there are numerous commonly shared rearrangements in the population (Figs. 4b, 5b, 6b, and 7b; Additional file 1: Table S3). Because we are not interested in simple deletions, we ignored gaps < 10 kb; we also tested a lower gap threshold (100 bp) which produced vastly more output at first, but after discarding rearrangements shared with the controls, the output size became closer to the default (*g* = 10 kb), suggesting that many of these gaps are shared with controls (Additional file 1: Fig. S5). Next, we merged (a.k.a. assembled) the reads of each group into a consensus sequence using lamassemble (https://gitlab.com/mcfrith/lamassemble) and realigned to the reference genome. Representative examples of detected rearrangements are shown with raw reads and consensus sequences in Additional file 1: Fig. S6. Computation time measurements for this method (including filtering with 33 controls) and comparison to different methods are shown in Additional file 1: Tables S4 and S5. Finally, we used dnarrange-link to infer the order and orientation of multiple read groups, to understand the whole rearrangement (Figs. 4c, 5c, e, and 6d, e).

### Patient 1

Patient 1 (case 2 in Nishimura et al. and Bano et al. case report) [18, 19] has de novo reciprocal translocation between chr2 and chrX, 46,X,t(X;2)(q22;p13) (Fig. 4a). The breakpoints were not detected by short read sequencing [17] though they were detected by more-painstaking breakpoint PCR [19], so we tested whether we could find this rearrangement with long reads. We performed PromethION DNA sequencing (112 Gb) and found 2773 groups of rearranged reads compared to human reference genome hg38. After subtracting rearrangements present in 33 controls, we found 80 patient-only read groups, of which two involve both chr2 and chrX (Fig. 4b). These are exactly the reciprocal chr2-X translocation (Fig. 4c, Additional file 1: Fig. S7a). The breakpoints agreed with reported breakpoints determined by Sanger sequencing (Additional file 1: Fig. S7b) [19].

The other 78 groups of rearranged reads are mostly tandem multiplications (duplications, triplications, etc.), tandem repeat expansion/evolution, deletions, retrotransposon insertions (five L1HS, four AluYa5, two AluYb8, three SVA, and one or two ERV-K LTRs), and other non-tandem duplications (Fig. 4d, e, Additional file 1: Table S6, Additional file 2: Table S12, Fig. S8). These types of retrotransposon are known to be active or polymorphic in humans [23–25]. We checked three AluYa5 insertions by PCR: all were confirmed (Additional file 1: Fig. S9). One insertion appears to be an orphan 3′-transduction from an L1HS in chr20: the L1HS was transcribed with readthrough into 3′ flanking sequence, then the 3′-end of this transcript (without any L1HS sequence) was reverse-transcribed and integrated into chr10 (Fig. 4e). Such orphan transductions can cause disease [26]. We also found an insertion of mitochondrial DNA (NUMT) into chr2 (Fig. 4e). Some of these rearrangements have been previously found in other humans, e.g., the ERV-K LTR inserted in chr12 [27]. Thus, our subtraction of rearrangements found in other humans was not thorough, especially because patient 1 is Caucasian whereas most of our controls (32/33) are Japanese.

### Patient 2

Patient 2 (case 1 in Nishimura et al.) [19] has reciprocal chromosomal translocation between chr4 and chrX: 46,X,t(X;4)(q21.3;p15.2), a 4-kb deletion in chrX, and a 7-kb deletion in chr4 (Fig. 5a). These were found previously by Southern blot combined with inverse PCR sequencing [19], but not by short read sequencing [17]. We performed PromethION DNA sequencing (117 Gb) and found 3336 groups of rearranged reads relative to the reference genome, which reduced to 33 groups after control subtraction (Fig. 5b). Only 2 out of 33 groups involve both chr4 and chrX: they show a reciprocal unbalanced chromosomal translocation exactly as described previously and confirmed by Sanger sequencing [17, 19] (Fig. 5c, Additional file 1: Fig. S10a,b). We examined DNA of the patient and parents by breakpoint PCR and confirmed that the translocation breakpoints occurred de novo (Additional file 1: Fig. S10b, c). Another of the 33 read groups shows a 43-kb deletion near the translocation site at chrX:107943899-107986412 (Fig. 5c, Additional file 1: Fig. S10a), which eliminates the *TEX13B* gene (Additional file 1: Fig. S10a), and was not previously described [17]. We found that this deletion is inherited from the father (Additional file 1: Fig. S10b, c). About half of the other rearrangements were tandem multiplications and retrotranspositions (Fig. 5d, Additional file 1: Fig. S11, Table S6, Additional file 2: Table S12). Three of the 33 groups lie near each other in chr11q11 (Fig. 5e): they have a unique order and orientation that produces one linear sequence, whereby we fully inferred the structure of this previously unknown rearrangement (Fig. 5e). This rearrangement has translocated and inverted fragments and three deletions, including a 10-kb deletion that removes most of the *TRIM48* gene. Breakpoint confirmation of this rearrangement by PCR and Sanger sequencing showed inheritance from the mother (Additional file 1: Fig. S12a, b).

### Patient 3: complex rearrangements at chr7-chr15 translocation

We next analyzed patient 3 whose precise structure of chromosomal translocations was only partly solved before [17, 20]. Patient 3 was reported to have reciprocal chromosomal translocation between chr7 and chr15 and also between chr9 and chr14, t(7;15)(q21;q15) and t(9,14)(q21;q11.2) (Fig. 6a) and has 4.6-Mb and ~ 1-Mb deletions on chr15 and chr7, respectively, which were predicted by microarray, although the precise locations of breakpoints were not detected in detail. We performed whole genome nanopore sequencing (95 Gb) on this patient and found 3351 groups of rearranged reads relative to the reference genome, which reduced to 43 groups after control subtraction (Fig. 6b). Fifteen out of 43 groups are involved in the two translocations: dnarrange-link found a unique way to order and orient them without changing the number of chromosomes (Fig. 6c, Additional file 1: Fig. S13). At first, there seem to be two read groups involving both chr9 and chr14, which accurately indicate the balanced chr9-chr14 translocation described previously [17]. However, dnarrange-link additionally identified a complex rearrangement for t(9,14)(q21;q11.2). A part of chr4 was unexpectedly inserted into derivative chr9 (Fig. 6d). This rearrangement was not investigated in the previous analyses, as chr7q21 was the primary locus for split-foot. In addition to this, dnarrange identified 8 out of 43 groups involving chr7 and chr15 (Fig. 6c, Additional file 1: Fig. S13). The order and orientation of these groups was difficult to determine by manual inspection, but dnarrange-link found only one possible way to connect them without changing the number of chromosomes (Fig. 6c). Finally, dnarrange-link could automatically reconstruct the whole rearrangements (Fig. 6d, e). The reconstructed rearrangements show that 3 fragments (breakpoint-to-breakpoint, asterisks in Fig. 6d, e) from chr4 and 1 fragment from chr14 were inserted into derivative chr9 (Fig. 6c, d), and 3 fragments from chr7 and 6 fragments from chr15 were inserted into derivative chr15 (Fig. 6c, e). They show 677-kb and 4.7-Mb deletions on chr7 and chr15, respectively, which were detected by microarray (Fig. 6e). Note that these deletions are not present in any part of the rearrangement, but only in the fully reconstructed rearrangement: they are holistic properties of the complex rearrangement. One candidate gene for split-foot, *SEM1*, was not disrupted, nor had altered expression in lymphoblastoid cells (Additional file 1: Fig. S14a, b).

A striking feature of these rearrangements is that the rearranged fragments come from near-exactly adjacent parts of the ancestral genome (Fig. 6d, e). This suggests that the rearrangements occurred by shattering of the ancestral genome into multiple fragments, which rejoined in a different order and orientation with loss of some fragments. Such shattering naturally explains why the fragments come from adjacent parts of the ancestor [10].

We performed Sanger sequence confirmation for all 18 breakpoints (Additional file 1: Fig. S15, primer sequences: Additional file 1: Table S2). There were only minor differences (usually 0 or 1 bases) between Sanger sequence-confirmed breakpoints and dnarrange-predicted breakpoints from lamassemble consensus sequences (Additional file 1: Fig. S16). Breakpoint junctions show blunt ends or microhomologies, which suggests nonhomologous or microhomology mediated end joining as reported in other congenital chromothripsis cases (Additional file 1: Table S7) [28, 29].

The other rearrangements are mostly local tandem duplications or insertions (Additional file 1: Table S6, Fig. S17, Additional file 2: Table S12). We found one processed pseudogene insertion, where exons of the *MFF* gene (chr10) were inserted into chr15 (Fig. 6g). Interestingly, there is also an AluYa5 insertion into chr15 nearby (Fig. 6g). Both Alu and processed pseudogene insertions are thought to be catalyzed by LINE-1-encoded proteins [30]: thus, we speculate that these two insertions did not occur independently.

### Patient 4: difficult case with translocation breakpoint in centromere repeat

Patient 4 had a reciprocal translocation between chr1 and chr9 (Fig. 7a). Breakpoints in chr1 were previously described at chr1:206,401,153 and chr1:206,402,729, which disrupted *SRGAP2*, by intensive investigations using fluorescent in situ hybridization (FISH), Southern hybridization and inverse PCR [21], or short read whole genome sequencing [17]. Chr9 breakpoints have not been found and were suspected to reside in repetitive centromeric heterochromatin. We performed PromethION DNA sequencing (41 Gb) and found 2523 groups of rearranged reads relative to the reference genome, which reduced to 14 after control subtraction, none of which indicates chr1-chr9 translocation (Fig. 7b, c, Additional file 1: Fig. S18, Additional file 2: Table S12). Dotplot pictures of reads that cross the chr1 breakpoint suggest that there is a reciprocal translocation, but the other half of the read aligns (with low confidence) to satellite or simple repeat sequences at centromeric regions on multiple different chromosomes (Fig. 7d, two example reads are shown). This limitation might be overcome by obtaining reads long enough to extend beyond the centromeric repeats, or perhaps by obtaining a reference genome that is more accurate in centromeric regions.

### Comparison to other tools

We also tried two existing structural variant (SV) detection methods: LAST-NanoSV [7] and ngmlr-Sniffles [31] (Additional file 1: Supplementary Methods). These methods mainly detect breakpoints and categorize them into 4 SV types (insertion, deletion, inversion, and duplication) or breakpoints (described as “BND”). Because there is no method to filter SVs that are present in controls using these tools, we manually examined breakpoints in the translocation sites predicted by G-band analysis.

In patient 1, ngmlr-Sniffles called two candidate breakpoints in the translocation site, but they were ± ~ 600 bp different from the Sanger sequence results and the reciprocal change was not detected (Additional file 3: Table S13). LAST-NanoSV could detect the breakpoints accurately, similarly to dnarrange (+lamassemble), with only − 1 ~ + 6 bp differences (Additional file 3: Table S13). It is not too surprising that LAST-NanoSV can detect the breakpoints similarly to dnarrange, because they are based on identical LAST alignments. We also examined LAST-NanoSV results for four TE integration examples in Fig. 4e (Additional file 1: Table S8). The AluYa5 integration was described as insertion (though “duplication” would be more precise); however, others are reported as BND and it was difficult to know if these are TE integrations. The AluYb8 integration has two different calls (insertion and BND) which could lead to misinterpretation (Additional file 1: Table S8).

In patient 2, ngmlr-Sniffles called four candidate breakpoints near the translocation sites but there were ± ~ 500 bp discordances from the Sanger sequence results. The most critical thing is that orientations were wrong and could cause misinterpretation of this reciprocal chromosomal translocation (Additional file 3: Table S13). LAST-NanoSV accurately detected the breakpoints similarly to dnarrange.

In patient 3, ngmlr-Sniffles missed several breakpoints which made it impossible to reconstruct this patient’s complex rearrangement (Additional file 3: Table S13). LAST-NanoSV detected all breakpoints (16 out of 18 with high confidence, i.e., “PASS”); however, it has no further function to reconstruct the rearranged genome: so it would be hard to understand this rearrangement, especially without filtering the numerous rearrangements shared with controls (Additional file 3: Table S13). We also checked the processed pseudogene/AluYa5 insertion in patient 3 (Fig. 6g) in NanoSV calls. The *MFF* gene (chr2) insertion into chr15 was described in 9 calls including deletion, insertion, and BND. The AluYa5 integration was not detected by NanoSV (Additional file 1: Table S8). This also illustrates the importance of understanding the whole rearrangement: NanoSV misleadingly reports deletions in chr2 for some of the removed introns in the processed pseudogene, and the distinctions between “insertion”, “BND”, etc. may be more confusing than helpful.

### Trio analysis

Among the control datasets, controls 1, 2, and 3 are a parent-child trio (Additional file 1: Table S1). By the same filtering as for patients 1–4, but without using controls 1–3, we obtained 27 groups of rearranged reads in the child (control 1). If the mother (control 3) is used for further filtering, nearly half of the groups (*n* = 14) are removed, and if the father is used as a control, the others (*n* = 12) are removed, except one (group23, Additional file 1: Table S9, Additional file 1: Fig. S19). The one remaining rearrangement is actually present in the mother, but not automatically filtered. This is an insertion of an SVA repeat, so its alignment to the genome is highly ambiguous and inconsistent between reads; thus, the shared rearrangement was not automatically recognized. We recognized it by manually investigating dnarrange results for reads aligned to this region. In summary, trio analysis is a powerful way to filter rearrangements.

### Re-analysis of deletions found from long reads

As a further test and comparison, we checked large deletions (more than 5 kb) in one human genome (NA12878) that were reported previously [13]. We used publicly available nanopore sequencing data (rel6, https://github.com/nanopore-wgs-consortium/NA12878/blob/master/Genome.md). Our pipeline without control filtering found rearrangements at the sites of all 30 reported deletions (Additional file 1: Table S10, Fig. S20). At 20/30 sites, we confirmed the presence of a simple deletion. Two other sites (1 and 13 in Additional file 1: Fig. S20) do not have deletions in NA12878 relative to the ancestral state, but rather have retrotransposon insertions in the reference genome (hg38). Sites 3 and 9 do not have simple deletions: they are more-complex rearrangements that include loss of sequence. Site 28 has a more-complex rearrangement with a larger deletion than reported. Sites 8 and 20 appear to have gene conversions, not simple deletions. At three sites (16, 18, 29), we find extremely complex rearrangements: these are in segmental duplications (large, recent duplications) and near assembly gaps in the reference genome. The rearrangements suggest rampant homologous recombination between the segmental duplicates, which is plausible, but the reference genome may not be reliable at these loci. In summary, we mostly confirm the previous results, but find greater complexity in some cases.

## Discussion

We analyzed a variety of chromosomal translocations in 4 patients, who were selected because previous studies had difficulty in determining precise breakpoints by conventional approaches including microarrays and short read sequencing. Especially, complex rearrangements in patient 3 were not solved even by intensive analysis [17, 20]. Our method could not only precisely detect breakpoints but also characterize how shattered fragments were ordered and oriented. To the best of our knowledge, there has been no method to filter patient-only rearrangements and connect them to reconstruct rearranged chromosomes from long read sequencing by an automatic algorithm. As we have shown, existing methods for long read sequencing (e.g., NanoSV) could only find breakpoints instead of SV types, which can be confusing in some cases (e.g., TE insertions; shown in Additional file 1: Table S8). In contrast, our method could semi-automatically find patient-only rearrangements and their types, which is indeed advantageous when looking for a potentially pathogenic rearrangement.

Recently, long read sequencing is becoming available for individual genome analysis due to a decrease in cost and increase in output data size. Accordingly, there have been a few approaches to use long read sequencing to detect structural variations [7, 10, 31], including tandem repeat changes in rare genetic diseases [6], providing evidence that long read sequencing has a clear advantage in precisely detecting rearrangements. We observed that multiple breakpoints were jointly detected in a single read in patient 3 (Additional file 1: Fig. S15d, e), because long enough reads can cover several breakpoints, which is helpful to phase and order rearrangements. There are continuous efforts to obtain longer nanopore reads; however, in case of complex rearrangements (e.g., chromothripsis), it is not easy to cover whole rearrangements, as seen in patient 3, by current read lengths. Our new tool, dnarrange-link, is useful to infer a complete picture of complex rearrangements. In addition, dnarrange-link can provide a clear visualization of reciprocal chromosomal translocations, inversions, or complex rearrangements with or without loss of sequence as seen in patients 1, 2, and 3. Most importantly, sequence loss was indicated after reconstructed derivative chromosomes were compared to the reference genome. We have shown that sequence losses in patient 3 agree with previously described microarray results. Previous studies on patient 3 predicted 802-kb deletion (microarray could only suggest ~ 1-Mb deletion due to low resolution), because a small inversion (arrow in Fig. 6e) was missed by previous studies using long PCR. We also presented an example in patient 1, who has an inverted duplication on chr16, which was only understood as copy number gain, or simply inversion, by microarray or conventional sequencing technologies (Additional file 1: Fig. S8, Additional file 2: Table S12). In summary, our approach using dnarrange and long read sequencing is superior to conventional approaches (e.g., microarray) because it can (1) connect multiple rearrangements, (2) subtract shared rearrangements, and (3) detect balanced chromosomal rearrangements (e.g., inversion). Recently, our pipeline fully characterized another chromothripsis more complex than that of patient 3, enabling diagnosis [32]: this shows our method is robust and useful in actual medical settings. We also showed a limitation of our method: detecting rearrangements in large repetitive regions beyond the length of long reads in patient 4. To date, there is no good method to detect rearrangements in large repetitive regions (e.g., centromeric or telomeric repeats) genome-wide. We hope our understanding of these still-intractable regions will expand as sequencing technologies advance.

Our approach in this study narrowed down patient-only rearrangements using 33 controls. The number of rearrangements decreased exponentially with the first few samples to a few hundreds. This may be due to the presence of common rearrangements in the population. We suspect large numbers of controls will not be needed if there is a target rearrangement locus (e.g., 4p15.2). In all 4 patients, patient-only (not present in at least 66 autosomal alleles of 33 controls) rearrangements were fewer than 100. If we were to further narrow down to ultra-rare variations that may cause rare congenital disorders, a larger number of controls may be considered. Patient 1 has more patient-only groups of rearranged reads (80) than the other patients (33, 43, and 14). This is because the patient is Caucasian and most of the control data used were Japanese (32/33 datasets). Applying ethnicity-matched controls, or parents or other relatives, will be useful to further remove benign rearrangements.

We noticed that large fractions of these rearrangements are insertions or tandem multiplications (Additional file 1: Table S6). Perhaps surprisingly, patient-specific simple inversions were uncommon. There are several types of insertions which are also known to cause diversity of human genomes [33], e.g., transposable element (TE) insertions, especially L1HS, AluYa5 or AluYb8 [34], ERV [35], nuclear mitochondrial DNA insertions (NUMT) [36], or processed pseudogene insertions [37] (Additional file 1: Supplementary Results, Fig. S21, Table S11). Interestingly, most of the inserted sequences were aligned to TEs. TE insertions may be a common type of rare variation seen in individuals. In addition to TE insertion, we detected rare processed pseudogene insertion in 3 patients. Two of these insertions were previously described with allele frequency 1–10% in Japanese (*MFF*) and 1–10% in non-Japanese (*MATR3*) [37]. We also observed non-tandem duplications that do not seem to be retrotranspositions: interestingly, about half of these are localized, i.e., a copy of a DNA segment is inserted near (e.g., within a few kb of) the original segment [10] (see blue highlighted loci in Additional file 2: Table S12).

Our analysis proves useful despite its dubious assumption that the reference genome is ancestral to the DNA reads. This may be partly because we focus on disease-causing rearrangements, which are likely to be derived. Also, incorrect rearrangements due to a non-ancestral reference may be found in both patients and controls, thus filtered out. It would be useful to construct a reference human genome that is ancestral (and complete), as far as possible, because this simplifies the relationship between the reference and extant human DNA sequences [10]. We also tested if dnarrange can detect reported deletions from a human genome (NA12878) that is widely used for benchmarking. Interestingly, 8/30 deletions show more complex structure than simple deletion/insertion, supporting the importance of considering more complex types of rearrangement [14].

There are some previously proposed methods for reconstructing rearranged genomes from short DNA reads, e.g., [38–40]. Our reconstruction method is simpler: the main difference is that we do not use data on the copy number of each part of the genome. These previous methods start from adjacencies between rearranged genomic segments, so they cannot (without modification) exploit the fact that one long read may already indicate the order and orientation of multiple adjacencies. Our reconstruction method is appropriate for the germline rearrangements we have encountered so far, but perhaps not for the complex and aneuploid rearrangements of cancer.

## Conclusions

We developed an effective method to find chromosomal aberration, with precise breakpoint identification, only from long read sequencing. Our method also provides an automatic algorithm for reconstruction of complex rearrangements. Long read sequencing may be considered when chromosomal abnormalities are suspected.

## Supplementary information

**Additional file 1.** Supplementary methods and results, Figs. S1–21, and Tables S1–11.

**Additional file 2: Table S12.** Detailed description of patient-only rearrangements

**Additional file 3: Table S13.** Comparison of the breakpoints.

## Data Availability

The patients’ sequence data is unavailable because we have not obtained consent for publishing even in a repository with restricted access. Publishing these sequencing data would violate the Japanese Personal Information Protection Act: http://www.japaneselawtranslation.go.jp/law/detail/?id=2781&vm=2&re=02. Source codes for dnarrange and lamassemble used and developed in this study are available under open source licenses: dnarrange: https://github.com/mcfrith/dnarrange [41] lamassemble: https://gitlab.com/mcfrith/lamassemble [42] Other web resources or tools used in this study are as follows: LAST: http://last.cbrc.jp MAFFT: https://mafft.cbrc.jp/alignment/software/ NCBI genome decoration: https://www.ncbi.nlm.nih.gov/genome/tools/gdp Primer3: http://bioinfo.ut.ee/primer3-0.4.0/ UCSC genome browser: https://genome.ucsc.edu/ ngmlr: https://github.com/philres/ngmlr nanoSV: https://github.com/mroosmalen/nanosv sniffles: https://github.com/fritzsedlazeck/Sniffles

## References

[1] Harada N, Hatchwell E, Okamoto N, Tsukahara M, Kurosawa K, Kawame H, Kondoh T, Ohashi H, Tsukino R, Kondoh Y (2004). Subtelomere specific microarray based comparative genomic hybridisation: a rapid detection system for cryptic rearrangements in idiopathic mental retardation. J Med Genet.

[2] Cameron DL, Di Stefano L, Papenfuss AT (2019). Comprehensive evaluation and characterisation of short read general-purpose structural variant calling software. Nat Commun.

[3] Schroder J, Wirawan A, Schmidt B, Papenfuss AT (2017). CLOVE: classification of genomic fusions into structural variation events. BMC Bioinformatics.

[4] Merker JD, Wenger AM, Sneddon T, Grove M, Zappala Z, Fresard L, Waggott D, Utiramerur S, Hou Y, Smith KS (2018). Long-read genome sequencing identifies causal structural variation in a Mendelian disease. Genet Med.

[5] Sanchis-Juan A, Stephens J, French CE, Gleadall N, Megy K, Penkett C, Shamardina O, Stirrups K, Delon I, Dewhurst E (2018). Complex structural variants in Mendelian disorders: identification and breakpoint resolution using short- and long-read genome sequencing. Genome Med.

[6] Sone J, Mitsuhashi S, Fujita A, Mizuguchi T, Hamanaka K, Mori K, Koike H, Hashiguchi A, Takashima H, Sugiyama H (2019). Long-read sequencing identifies GGC repeat expansions in NOTCH2NLC associated with neuronal intranuclear inclusion disease. Nat Genet.

[7] Cretu Stancu M, van Roosmalen MJ, Renkens I, Nieboer MM, Middelkamp S, de Ligt J, Pregno G, Giachino D, Mandrile G, Espejo Valle-Inclan J (2017). Mapping and phasing of structural variation in patient genomes using nanopore sequencing. Nat Commun.

[8] Smith CE, Llorente B, Symington LS (2007). Template switching during break-induced replication. Nature.

[9] Hastings PJ, Lupski JR, Rosenberg SM, Ira G (2009). Mechanisms of change in gene copy number. Nat Rev Genet.

[10] Frith MC, Khan S (2018). A survey of localized sequence rearrangements in human DNA. Nucleic Acids Res.

[11] Hamada M, Ono Y, Asai K, Frith MC (2017). Training alignment parameters for arbitrary sequencers with LAST-TRAIN. Bioinformatics.

[12] Frith MC, Kawaguchi R (2015). Split-alignment of genomes finds orthologies more accurately. Genome Biol.

[13] Audano PA, Sulovari A, Graves-Lindsay TA, Cantsilieris S, Sorensen M, Welch AE, Dougherty ML, Nelson BJ, Shah A, Dutcher SK (2019). Characterizing the major structural variant alleles of the human genome. Cell.

[14] Collins RL, Brand H, Redin CE, Hanscom C, Antolik C, Stone MR, Glessner JT, Mason T, Pregno G, Dorrani N (2017). Defining the diverse spectrum of inversions, complex structural variation, and chromothripsis in the morbid human genome. Genome Biol.

[15] Beck CR, Carvalho CMB, Akdemir ZC, Sedlazeck FJ, Song X, Meng Q, Hu J, Doddapaneni H, Chong Z, Chen ES (2019). Megabase length hypermutation accompanies human structural variation at 17p11.2. Cell.

[16] Eisfeldt J, Pettersson M, Vezzi F, Wincent J, Kaller M, Gruselius J, Nilsson D, Syk Lundberg E, Carvalho CMB, Lindstrand A (2019). Comprehensive structural variation genome map of individuals carrying complex chromosomal rearrangements. PLoS Genet.

[17] Suzuki T, Tsurusaki Y, Nakashima M, Miyake N, Saitsu H, Takeda S, Matsumoto N (2014). Precise detection of chromosomal translocation or inversion breakpoints by whole-genome sequencing. J Hum Genet.

[18] Bano G, Mansour S, Nussey S (2008). The association of primary hyperparathyroidism and primary ovarian failure: a de novo t(X; 2) (q22p13) reciprocal translocation. Eur J Endocrinol.

[19] Nishimura-Tadaki A, Wada T, Bano G, Gough K, Warner J, Kosho T, Ando N, Hamanoue H, Sakakibara H, Nishimura G (2011). Breakpoint determination of X;autosome balanced translocations in four patients with premature ovarian failure. J Hum Genet.

[20] Saitsu H, Kurosawa K, Kawara H, Eguchi M, Mizuguchi T, Harada N, Kaname T, Kano H, Miyake N, Toda T, Matsumoto N (2009). Characterization of the complex 7q21.3 rearrangement in a patient with bilateral split-foot malformation and hearing loss. Am J Med Genet A.

[21] Saitsu H, Osaka H, Sugiyama S, Kurosawa K, Mizuguchi T, Nishiyama K, Nishimura A, Tsurusaki Y, Doi H, Miyake N (2012). Early infantile epileptic encephalopathy associated with the disrupted gene encoding Slit-Robo Rho GTPase activating protein 2 (SRGAP2). Am J Med Genet A.

[22] Frith M. C MS, Katoh K. lamassemble: multiple alignment and consensus sequence of long reads. Methods Molecular Biol 2020. in press. 10.1007/978-1-0716-1036-7_9 33289891

[23] Mills RE, Bennett EA, Iskow RC, Devine SE (2007). Which transposable elements are active in the human genome?. Trends Genet.

[24] Kvikstad EM, Piazza P, Taylor JC, Lunter G (2018). A high throughput screen for active human transposable elements. BMC Genomics.

[25] Stewart C, Kural D, Stromberg MP, Walker JA, Konkel MK, Stutz AM, Urban AE, Grubert F, Lam HY, Lee WP (2011). A comprehensive map of mobile element insertion polymorphisms in humans. PLoS Genet.

[26] Solyom S, Ewing AD, Hancks DC, Takeshima Y, Awano H, Matsuo M, Kazazian HH (2012). Pathogenic orphan transduction created by a nonreference LINE-1 retrotransposon. Hum Mutat.

[27] Marchi E, Kanapin A, Magiorkinis G, Belshaw R (2014). Unfixed endogenous retroviral insertions in the human population. J Virol.

[28] Kloosterman WP, Guryev V, van Roosmalen M, Duran KJ, de Bruijn E, Bakker SC, Letteboer T, van Nesselrooij B, Hochstenbach R, Poot M, Cuppen E (2011). Chromothripsis as a mechanism driving complex de novo structural rearrangements in the germline. Hum Mol Genet.

[29] Kloosterman WP, Tavakoli-Yaraki M, van Roosmalen MJ, van Binsbergen E, Renkens I, Duran K, Ballarati L, Vergult S, Giardino D, Hansson K (2012). Constitutional chromothripsis rearrangements involve clustered double-stranded DNA breaks and nonhomologous repair mechanisms. Cell Rep.

[30] Richardson SR, Doucet AJ, Kopera HC, Moldovan JB, Garcia-Perez JL, Moran JV: The influence of LINE-1 and SINE retrotransposons on mammalian genomes. Microbiol Spectr 2015, 3:MDNA3–0061-2014. 10.1128/microbiolspec.MDNA3-0061-2014 PMC4498412 26104698

[31] Sedlazeck FJ, Rescheneder P, Smolka M, Fang H, Nattestad M, von Haeseler A, Schatz MC (2018). Accurate detection of complex structural variations using single-molecule sequencing. Nat Methods.

[32] Lei M, Liang D, Yang Y, Mitsuhashi S, Katoh K, Miyake N, Frith MC, Wu L, Matsumoto N. Long-read DNA sequencing fully characterized chromothripsis in a patient with Langer-Giedion syndrome and Cornelia de Lange syndrome-4. J Hum Genet. 2020;65:667–74. 10.1038/s10038-020-0754-6 PMC7324355 32296131

[33] Sudmant PH, Rausch T, Gardner EJ, Handsaker RE, Abyzov A, Huddleston J, Zhang Y, Ye K, Jun G, Fritz MH (2015). An integrated map of structural variation in 2,504 human genomes. Nature.

[34] Carroll ML, Roy-Engel AM, Nguyen SV, Salem AH, Vogel E, Vincent B, Myers J, Ahmad Z, Nguyen L, Sammarco M (2001). Large-scale analysis of the Alu Ya5 and Yb8 subfamilies and their contribution to human genomic diversity. J Mol Biol.

[35] Wildschutte JH, Williams ZH, Montesion M, Subramanian RP, Kidd JM, Coffin JM (2016). Discovery of unfixed endogenous retrovirus insertions in diverse human populations. Proc Natl Acad Sci U S A.

[36] Tsuji J, Frith MC, Tomii K, Horton P (2012). Mammalian NUMT insertion is non-random. Nucleic Acids Res.

[37] Ewing AD, Ballinger TJ, Earl D, Broad Institute Genome S, Analysis P, Platform, Harris CC, Ding L, Wilson RK, Haussler D: Retrotransposition of gene transcripts leads to structural variation in mammalian genomes. Genome Biol 2013, 14:R22. 10.1186/gb-2013-14-3-r22 PMC3663115 23497673

[38] Oesper L, Ritz A, Aerni SJ, Drebin R, Raphael BJ (2012). Reconstructing cancer genomes from paired-end sequencing data. BMC Bioinformatics.

[39] Yasuda T, Miyano S (2015). Inferring the global structure of chromosomes from structural variations. BMC Genomics.

[40] Eitan R, Shamir R (2017). Reconstructing cancer karyotypes from short read data: the half empty and half full glass. BMC Bioinformatics.

[41] Frith, MC. dnarrange. Github. https://github.com/mcfrith/dnarrange (2019).

[42] Frith, MC. lamassemble. GitLab https://gitlab.com/mcfrith/lamassemble (2019).

