# Supplementary material for: A pipeline for complete characterization of complex germline rearrangements from long DNA reads
Source: Genome Med. 2020 Jul 31;12:67. doi: 10.1186/s13073-020-00762-1 (PMC7393826; doi:10.1186/s13073-020-00762-1)
Supplement: Supplementary file 1 — Additional file 1. Supplementary methods and results, Figs. S1–21, and Tables S1–11. [file 13073_2020_762_MOESM1_ESM.pdf]

Supplementary information

A pipeline for complete characterization of complex germline rearrangements from long DNA reads

Satomi Mitsuhashi[1#], Sachiko Ohori[1#], Kazutaka Katoh[2,3], Martin C Frith[3-5,*], Naomichi Matsumoto[1*]

1. Department of Human Genetics, Yokohama City University Graduate School of Medicine
2. Research Institute for Microbial Diseases, Osaka University, Suita, Japan
3. Artificial Intelligence Research Center, National Institute of Advanced Industrial Science and Technology (AIST)
4. Graduate School of Frontier Sciences, University of Tokyo
5. Computational Bio Big-Data Open Innovation Laboratory (CBBD-OIL), AIST

# Contributed equally

To whom correspondence should be addressed:

Martin C Frith, PhD

Artificial Intelligence Research Center

National Institute of Advanced Industrial Science and Technology (AIST)

2-3-26 Aomi, Koto-ku, Tokyo, 135-0064, Japan

Telephone: +81-3-3599-8001

Fax: +81-3-5530-2061

E-mail: mcfrith@edu.k.u-tokyo.ac.jp

Naomichi Matsumoto, MD, PhD
Department of Human Genetics
Yokohama City University Graduate School of Medicine
Fukuura 3-9, Kanazawa-ku, Yokohama, 236-0004, Japan
Telephone: +81-45-787-2606
Fax: +81-45-786-5219
E-mail: naomat@yokohama-cu.ac.jp

Ancestral genome

Here, we clarify the concept of "ancestral reference genome" mentioned in the main text. An ancestral human genome sequence could be constructed in the following way. For each known genetic variant (e.g. an inversion) in the extant human population, determine which allele is ancestral (e.g. by comparison to ape genomes), and put that allele in the reference. Note that the genome constructed in this way may never have existed.

No doubt, this construction cannot be perfectly finished, especially in genome regions with complex recent rearrangements. Nevertheless, ancestral alleles could be determined in many simple cases, and this would already be useful.

For example, consider a deletion variant, where an ancestrally-present 1 kb segment is deleted in some extant genomes. It is useful for this segment to be present in the reference genome. If it is absent, analysis of genomes where the segment is present is difficult: in particular, the segment may be incorrectly compared to paralogous parts of the reference.

The reader may wonder about the converse situation: a 1 kb insertion, which was ancestrally absent. Here, it is important to consider that deletions and insertions are not entirely symmetric. Deletion of large, non-repetitive segments is usual. On the other hand, insertion of a large non-repetitive segment, not derived from any ancestral segment, is unusual. Typically, sequences are descended from ancestral sequences, rather than appearing de novo. Thus, a large "insertion" is more likely to be a duplication or translocation, for example of a transposable element. Analysis of such sequence changes is tractable, for example the retrotransposon integrations characterized in this study.

There is certainly room for debate about the practical merits of an ancestral reference, but the idea at least merits consideration.

Supplementary Methods

Clinical details of the patients

Patient 1

Detailed clinical information was described elsewhere [1, 2]. Briefly, the patient was a Caucasian female. She was 40 years old at the time of the previous study. She was delivered at 37 weeks of gestation with a birth weight of 2,930g. She presented with amenorrhea at age 17. Primary ovarian failure was indicated by hormonal level and her hypoplastic uterus with bilateral streak gonads were found by laparotomy. G-banded chromosomal analysis showed a balanced reciprocal translocation 46X, t(X;2) (q22;p13). Array CGH analysis using the Agilent 4×44K oligo array platform at a resolution of 44K showed no deletions at the breakpoint. She was taller than other members of her family (177cm, 98th tile). She got pregnant by in vitro fertilization with egg donation at the age of 36 years. She underwent parathyroidectomy at 24-week gestation due to primary hypoparathyroidism.

Patient 2

Detailed clinical information was described elsewhere [2]. Briefly, the patient was a female born to non-consanguineous Japanese parents and was 38 years old at the time of the previous study. She was born with neonatal asphyxia. She had her first menstrual period once at 14 years old, but then became amenorrheic and started on hormonal replacement therapy. She was diagnosed with primary hyperthyroidism at the age of 18 years and underwent subtotal thyroidectomy and started thyroid hormonal therapy. G-banded chromosomal analysis showed a balanced reciprocal translocation 46X, t(X;4)(q21.3;p15.2) at the age of 38 years.

Patient 3

Detailed clinical information was described elsewhere [3]. Briefly, the patient was a girl to non-consanguineous Japanese parents and was 9 years old at the time of the previous

study. She had no family with SHFM. She was delivered at 38 weeks of gestation after an uneventful pregnancy with a birth weight of 2,850g (-0.02SD), length of 48cm (-0.4SD), and occipitofrontal circumference of 32cm (-0.5SD). She was admitted to a hospital for weak sucking when she was one month old. She showed cutaneous syndactyly of 4th and 5th digits of the right foot and 1st, 2nd, 4th and 5th digits of the left foot. Her hands were normal. In addition, she had strabismus, micrognathia, full lower lip, bilateral ear canal stenosis, a severe mixed type deafness, and developmental disorder. She walked alone at 21 months. Her developmental stage at 25 months was equal to 7-8 months. Since 3 years of age, self-injuries, hyperactivity, and sleep disorders appeared.

Patient 4

Detailed clinical information was described elsewhere [4]. Briefly, the patient was a girl to non-consanguineous Japanese parents and was 5 years old at the time of the previous study. She was delivered at term without asphyxia after an uneventful pregnancy. She started clonic convulsions of extremities 2 days after birth. She was diagnosed with WEST syndrome at 5 months of age. She had intellectual disability without head control, series of tonic-spasms, and hypsarrhythmia on Electroencephalogram.

`dnarrange` details

As stated in the main text, `dnarrange` performs these three steps:

1. Discard any patient read that has any two rearranged fragments in common with any control read.
2. Discard any patient single read that has any rearrangement not shared by any other patient read. More precisely: discard any patient read that has a pair of consecutive rearranged fragments not shared by any other patient read.
3. Group patient reads that cover the same rearrangement. Discard groups with fewer than *s* reads. (In this study, *s*=3.)

`dnarrange` assumes that it is given read-to-genome alignments with this property, which is

guaranteed by `last-split`: each read base is aligned to at most one genome base. In other words, the alignments indicate the unique source, in the assumed-ancestral reference genome, of each part of the read.

In detail, `dnarrange` performs these steps:

1. In order to recognize large "deletions" as rearrangements, if an alignment has deletions >= *g* (a threshold; default 10kb), split it into separate alignments either side of these deletions.
2. Get rearranged reads. We classify rearrangements into four types (Additional file 1: Fig. S1): inter-chromosome, inter-strand (if a read's alignment jumps between the two strands of a chromosome), non-colinear (if a read's alignment jumps backwards on the chromosome), and "big gap" (if a read's alignment jumps forwards on the chromosome by >= *g*). The reason for excluding gaps < *g* is simply that we wish to focus on complex rearrangements rather than simple deletions.
3. Discard any patient single read that shares a rearrangement with any control read. The precise criteria for "shares a rearrangement" are in the next subsection.
4. Discard any patient read with any rearrangement not shared by any other patient read (precise criteria below). Repeat this step until no further reads are discarded (so that `dnarrange` has the useful property of *idempotence*).
5. Group patient reads that share rearrangements. First, a link is made between any pair of reads that share a rearrangement. Then, groups are connected components, i.e. sets of reads linked directly or indirectly.
6. Discard groups with fewer than *s* reads.

Definition of two reads sharing a rearrangement

Two reads *R* and *S* (Additional file 1: Fig. S2) are deemed to share a rearrangement if:

1. The alignments of *R* to the genome include two alignments *A* and *B*, and the alignments of *S* include *X* and *Y*, such that:

2. *A* overlaps *X* in the genome.
3. *B* overlaps *Y* in the genome.
4. *A* and *B* exhibit one of the four rearrangement types (inter-chromosome, inter-strand, non-colinear, or big gap), and *X* and *Y* exhibit the same rearrangement type.
5. If the rearrangement type is non-colinear (jumps backwards in the chromosome) or big gap (jumps forwards in the chromosome):
    1. The chromosome range jumped between *A* and *B* overlaps the range jumped between *X* and *Y*.
    2. The number of chromosome bases jumped between *A* and *B* is <= 2x that between *X* and *Y*, and vice-versa.
6. The alignments have consistent strandedness. Strandedness means: which chromosome strand the read aligns to. "Consistent" means that either: *A* and *X* have the same strandedness and so do *B* and *Y*, or: *A* and *X* have opposite strandedness and so do *B* and *Y*.
7. The alignments' order in their reads is consistent with the strandedness. If the alignments have the same strandedness they must occur in the same order in their reads, else they must occur in the opposite order.
8. The number of bases in read *R* between *A* and *B* is close to the number of bases in read *S* between *X* and *Y*. Specifically: abs(s-r+m-n) <= d (default 1000), where s, r, m, and n are defined in Additional file 1: Fig. S2.

Discarding any read with any rearrangement not shared by another read

For each read: check each pair of alignments (*A* and *B*) that occur *consecutively* in the read and exhibit one of the four rearrangement types (Additional file 1: Fig. S2). Require that this rearrangement is shared (as defined above) by a pair of alignments (*X* and *Y*) that occur *consecutively* in another read.

Miscellaneous `dnarrange` details

Two alignments are considered to be on different chromosomes only if the chromosomes are known to be different. E.g. "chr7" and "chrUn" are not known to be different, but "chr7" and "chr5_random" are. The non-colinear rearrangement type is not considered for chrM, which is circular. Two alignments *A* and *B* of read *R* are not deemed to exhibit a "big gap" if any other alignment is between them in read *R* (Additional file 1: Fig. S2).

Limitations of `dnarrange`

1. It may have trouble finding patient-specific rearrangements that are close to rearrangements shared with controls. This is because, if a patient read shares a rearrangement with a control read, the patient read is discarded.
2. It will not work well with extremely long reads, or assembled chromosomes. This is because it starts by seeking reads that contain patient-specific rearrangements and lack rearrangements shared with controls. It may work best with a mixture of shorter reads (to separate nearby rearrangements) and longer reads (to span huge repeats, and know the order and orientation of rearranged fragments unambiguously).
3. It is not really designed to find transposable element insertions. If two reads overlap the same TE insertion, they may get aligned to different source TEs in the genome, because this alignment is highly ambiguous. Then `dnarrange` will not consider these reads to share a rearrangement, so will not group them. This could be fixed by loosening the criteria for sharing a rearrangement, at a risk of retaining many artefactual rearrangements (e.g. Additional file 1: Fig. S3).

`dnarrange-link`

`dnarrange-link` infers the order and orientation of read groups that are suspected to cover parts of a larger rearrangement. In other words, it infers how the groups are linked to each other, and thereby reconstructs the derived chromosomes. It uses (the alignments of) one representative read per group. The representative could be one actual read, or a consensus sequence (in this study, a `lamassemble` consensus sequence). Based on the

alignments, the two ends of each read are classified as "left" if the alignment extends rightwards/downstream along the chromosome starting from that end (shown as "[" in Additional file 1: Fig. S4) or "right" if the alignment extends leftwards/upstream ("]"). Two ends may be directly linked only if:

- They are on the same reference chromosome.
- One is left and the other is right.
- The left end is downstream of (has higher reference coordinate than) the right end.

In order to infer the actual links, we require some further information or assumption. We make this assumption: there are as many links as possible, or equivalently, the derived genome has as few chromosomes as possible. For example, in Additional file 1: Fig. S4a, B1 may be linked to C2, but in that case it becomes impossible to link C1 to anything, and D1 to anything. Based on our assumption, we instead link B1 to C1 and D1 to C2. In this example, `dnarrange-link` infers two derivative chromosomes: one is reconstructed from two reads by linking A2 to E1, the other is reconstructed from three reads by linking D1 to C2 and C1 to B1 (Additional file 1: Fig. S4b).

The two types of end, with linkability relationship, define a bipartite graph. To infer the links based on our assumption, we find a "maximum matching" in this graph. If there is more than one maximum matching, one is chosen arbitrarily, and a warning message is printed. In Additional file 1: Fig. S4, there is only one maximum matching

In Additional file 1: Fig. S4a, the left and right ends occur in an alternating pattern along each reference chromosome. In this case, we get a unique maximum matching by linking adjacent left and right ends. This alternating pattern seems to occur often in practice

Coordinates of left and right ends

`dnarrange-link` needs to decide whether a left end is "downstream of" a right end, i.e. whether it is rightwards/downstream in the chromosome. We wish to allow for some overlap,

and some slop in the alignments. In the current version, `dnarrange-link` crudely defines the chromosome coordinate of a (left or right) end as: the average of the start and end coordinates of the alignment at that end.

Alignment to human reference genome

Reads were aligned to human reference genome (hg38) using LAST version 959 (http://last.cbrc.jp) as follows. First, the genome was analyzed by WindowMasker [5] and a converted into a LAST database:

```
windowmasker -mk_counts -in hg38.fa > hg38.wmstat
windowmasker -ustat hg38.wmstat -outfmt fasta -in hg38.fa > hg38-wm.fa
lastdb -P8 -uNEAR -R11 -c hg38 hg38-wm.fa
```

This LAST database can be re-used for any future reads. Then, `last-train` was used to determine the rates of small insertions, deletions, and each kind of substitution between reads and genome:

```
last-train -P8 hg38 reads.fa > train.out
```

This training result can be re-used for any future reads that are expected to have the same rates (e.g. same sequencing hardware and base-calling software). Finally, the alignments were determined by:

```
lastal -P8 -p train.out hg38 reads.fa | last-split –m1 > alns.maf
```

(Since LAST version 983, "`-m1`" can be omitted, because it is the default setting.)

Finding reads with translocations and complex rearrangements

Rearrangements were found using `dnarrange` (https://github.com/mcfrith/dnarrange):

```
dnarrange –s3 patient.maf > patient-groups.maf
```

`dnarrange` finds rearranged reads, and groups those reads that share a rearrangement. To remove rearrangements that are shared by other individuals, we used 33 controls (Figure 3, Additional file 1: Table S1):

```
dnarrange –s3 patient.maf : control.maf > patient-only.maf
```

Option –s3 means that the minimum number of supporting reads per group is 3.

The patient-only groups file was re-analyzed with option –c1 to remove unreliable reads (Additional file 1: Fig. S3):

```
dnarrange –c1 –s3 patient-only.maf > final.maf
```

Drawing dot-plot pictures of each group of rearranged reads

Dot plot pictures were obtained like this with some modification:

```
last-multiplot final.maf final-pic
```

Modified `last-dotplot` options were, for example:

```
last-dotplot -1 chr1:149390802-149390842 --sort2=3 --strands2=1 --rot1=v
--rot2=h --labels1=2 --rmsk1 rmsk.txt --genePred1 refFlat.txt
alignment.maf alignment.png
```

To draw gray lines joining breakpoints, this option was used: `--join=2`

Assembly of reads and breakpoint detection

Each group of rearranged reads was merged into a consensus sequence like this:

```
dnarrange-merge reads.fa train.out dnarrange-output > consensus.fa
```

Consensus sequences were re-aligned to the unmasked reference genome with these commands:

```
lastdb -P8 -uNEAR -R01 hg38 hg38.fa
last-train -P8 hg38 consensus.fa > train.out
lastal -P8 -p train.out hg38 consensus.fa | last-split -m1 > re-alns.maf
```

Breakpoints were determined from the re-aligned file:

```
dnarrange –s1 re-alns.maf > consensus-rearrangements
```

Then, dot-plot pictures were produced like this:

```
last-multiplot consensus-rearrangements dotplot-picture
```

`lamassemble` method

`lamassemble` merges overlapping DNA reads into a consensus sequence, by these steps:

1. Calculate the rates of insertion, deletion, and substitutions between two reads by "doubling" the rates from `last-train`, because errors occur in both reads.
2. Use these rates to find pairwise alignments between the reads with LAST. LAST also calculates the probability that each pair of bases is wrongly aligned (which is high when there are alternative alignments with near-equal likelihood).
3. Use the LAST alignments in descending order of score to define a tree for progressive alignment by MAFFT.
4. Constrain the MAFFT alignment by anchoring pairs of bases that were aligned by LAST with error probability <= 0.002.
5. Make a consensus sequence from the MAFFT alignment. Omit alignment columns with gaps in > 50% of sequences covering that column. For each column, get the base that maximizes prob (base|column), using the `last-train` substitution probabilities.

Some results using a prototype of `lamassemble` were published previously [6].

`lamassemble` details

"Doubling" of substitution probabilities in `lamassemble`

From `last-train`, we have a 4x4 matrix P(x,y): the probability of read base y aligned to genome base x. These 16 probabilities sum to 1. Let us define c(x) to be the complement of base x, and G(x) the probability of base x in the genome: G(x) = sum(y in a,c,g,t) P(x,y). For the subsequent steps to make sense, we require that G has parity: G(x) = G(c(x)).

`lamassemble` forces parity by rescaling:

G'(x) = [G(x) + G(c(x))] / 2

P'(x,y) = P(x,y) * G'(x) / G(x)

It then calculates the probability of base x in a read forward strand aligned to base y in a read forward strand:

F(x,y) = sum(z in a,c,g,t) [P'(z,x) * P'(z,y) / G'(z)]

And the probability of x in a read forward strand aligned to y in a read reverse strand:

R(x,y) = sum(z in a,c,g,t) [P'(z,x) * P'(c(z),c(y)) / G'(z)]

"Doubling" of gap probabilities in `lamassemble`

`last-train` calculates read-to-genome gap probabilities like this[7]:

delOpenProb = delOpenCount / n

insOpenProb = insOpenCount / n

delExtendProb = (delCount - delOpenCount) / delCount

insExtendProb = (insCount - insOpenCount) / insCount

`lamassemble` crudely calculates these read-to-read gap probabilities:

gapOpenProb = 1 - (1 - delOpenProb) * (1 - insOpenProb)

gapExtendProb = (gapCount - gapOpenCount) / gapCount

where:

gapCount = delCount + insCount

gapOpenCount = delOpenCount + insOpenCount

By basic algebra, we can calculate gapExtendProb in terms of the four gap probabilities from `last-train`.

Alignment in `lamassemble`

`lamassemble` finds pairwise alignments between the reads like this:

```
lastdb -uNEAR -c -R01 -W19 my_db seq_file
lastal -j4 -D1e9 -m5 -z30g -s1 -p fwd_scores my_db seq_file
lastal -j4 -D1e9 -m5 -z30g -s0 -p rev_scores my_db seq_file
```

where the 1st `lastal` command compares read forward strands to each other, and the 2nd `lastal` command compares forward strands to reverse strands. Alignments of a sequence to itself are discarded.

The alignments are sorted in descending order of score. For each alignment in turn:

* Record a link between the 2 aligned sequences, only if they are not yet linked directly or indirectly. This link defines the next step of progressive alignment. The (forward or reverse) strands in the alignment define which strands will be used for progressive alignment.

* If the alignment uses forward/reverse strands inconsistent with strands aligned (directly or indirectly) by previous alignments: discard this alignment.

* If the alignment is not "roughly colinear" with previous alignments of these 2 sequences: discard it. Two alignments A and B, between sequences S and T, are "roughly colinear" if:

start_coordinate(A,S) < start_coordinate(B,S)
start_coordinate(A,T) < start_coordinate(B,T)
end_coordinate(A,S) < end_coordinate(B,S)
end_coordinate(A,T) < end_coordinate(B,T)

Before running MAFFT, `lamassemble` trims bases at the start and end of each sequence that are outside any non-discarded LAST alignment.

Inferring retrotransposition

We inferred that many of the patient-specific rearrangements are retrotransposon integrations, by manual inspection and comparison to genome annotation from RepeatMasker (http://www.repeatmasker.org). It is not necessarily easy to distinguish retrotransposition from other types of rearrangements that happen to overlap retrotransposons. Our main criterion was whether the rearrangement involves a retrotransposon of a type known to be active or polymorphic (e.g. L1HS, AluYa5, AluYb8, SVA, ERVK). These elements are a tiny fraction of genomic repeats (e.g. ~0.2% of L1 annotations are L1HS, ~0.2% of Alus are AluYb8, ~0.3% of Alus are AluYa5), but overlap a large fraction of our rearrangements. Moreover, some rearrangements are near-exact

insertions of whole retrotransposons (e.g. Additional file 1: Fig. S8, group 60, 61, 65, 72): this would be an extreme coincidence if it were not retrotransposition of such an element. Retrotransposition often exhibits 5'-truncation [8]: accordingly, we observe insertions that coincide with the 3'-end of a retrotransposon (e.g. Additional file 1: Fig. S11, group 26). A thorough survey of retrotransposition could consider other hallmarks, such as target site duplication and LINE-1 endonuclease consensus sequence [8].

Gene expression levels in lymphoblastoid cell

Total RNA was extracted from lymphoblastoid cells from Patient 3, and 3 controls, using RNeasy Plus Mini Kit (QIAGEN, Hilden, Germany), then subjected to reverse-transcription reaction using SuperScriptIII (Thermo Fisher Scientific). Quantitative real-time PCR was performed using Rotor-Gene SYBR Green PCR Kit and Rotor-Gene (QIAGEN, Hilden, Germany). Primers used were described in Additional file 1: Table S2. delta-delta CT method was used to compare gene expression levels of *SEM1*.

Breakpoint detection by NanoSV and Sniffles

`NanoSV` website (https://github.com/mroosmalen/nanosv) states: "we found that LAST alignments give the most accurate results for SV calling with NanoSV". Thus we used the same alignment file we used for dnarrange. `NanoSV` -1.2.3 was installed via miniconda3, then run like this:

```
NanoSV -t 8 -s path-to-samtools -b hg38.bed -o out.vcf input.sorted.bam
```

NanoSV results using LAST alignment is named LAST-NanoSV hereafter.

For Sniffles, ngmlr was used to align long reads to reference genome. Then SVs are found by Sniffles.

`ngmlr` -0.2.4 (https://github.com/philres/ngmlr) and `sniffles` -1.0.11 (https://github.com/fritzsedlazeck/Sniffles) were used like this:

```
ngmlr -t 16 -r hg38 -q input.fa -x ont -o out.sam
sniffles -m out.sorted.bam -v out.vcf -s 3
```

The results are named ngmlr-Sniffles hereafter.

Supplementary Results

Gene expression level of possible causative genes in Patient 3

Patient3 had a phenotype of split-hand/foot malformation (SHFM) with hearing loss, delayed development, self-injuries, hyperactivity and sleep disorders. SHFM has several causative loci, and one has been mapped to 7q21.3-q22.1[9]. Three genes *SEM1*, *DLX5* and *DLX6* are suggested to be related to SHFM with hearing impairment[10]. None of the three genes are disrupted in this patient (Additional file 1: Fig. S14a), which is also true of other SHFM patients, suggesting some regulatory effect of the rearrangement of flanking regions in SHFM[10]. To test the effect of the complex rearrangement on expression of those genes, we analyzed mRNA expression levels using the patient's lymphoblastoid cells (LCL). As we found out that *SEM1* was expressed in LCLs, we analyzed expression level of *SEM1*. Expression levels of *SEM1* cDNA were evaluated by quantitative polymerase chain reaction (qPCR) in the patient and three healthy controls. *SEM1* expression of LCLs in this patient was not lower than the controls (Additional file 1: Fig. S14b). We could not test *DLX5* and *DLX6* because those genes were not expressed in LCLs. It is possible these two gene(s) are contributing to disease pathogenesis.

TE insertions, processed pseudogene insertions and nuclear mitochondrial sequences

Large fractions of patient-specific rearrangements were smaller-scale rearrangements including tandem duplications or insertions. Among insertions, many of them were annotated as transposable elements, especially L1HS, AluYa5 or AluYb8 (Additional file 2: Table S12). Comparison of the fraction of patient-only TE-insertions (patients) to all TEs from RepeatMasker annotations (rmsk) from the UCSC genome browser (https://genome.ucsc.edu), suggests that these active TEs are enriched in patient-only insertions (Additional file 1: Fig. S21), supporting the notion that recently integrated TEs are the source of these genomic variations. In addition, we observed an ERVK insertion in Patient1 (Fig 4e), which was previously described[11]. We also identified an SVA insertion

shared by patients 2 and 3 (Additional file 2: Table S12: group1 in Patient 2 and group6 in Patient 3).

Aside from these TE insertions, there were insertions which were aligned to multiple exons of genes that are located distant from the insertion sites, possibly due to processed pseudogene insertion, in 3 patients (Figure 6g, Additional file 1: Fig. S11, S18). Insertions were from exons of *MFF*, *MATR3* and *FXR1* genes, in patient 2, 3 and 4, respectively. *MFF* and *MATR3* processed pseudogene insertions into these loci were described previously[12], but not *FXR1*. This kind of rare structural variation might be commonly present in our genomes because we observed it in 3/4 patients in this study, although further study of multiple individuals may be necessary to conclude.

We also observed nuclear mitochondrial sequence (NUMT) in Patients 1 and 2 (Fig 4e, Additional file 1: Fig. S11, Table S11, Additional file 2: Table S12). Two of them are inserted into LINE regions of introns of *SPAG16* and *CEP128*, respectively. In all NUMTs, there were flanking A-T oligomers at the insertion loci as suggested previously (Additional file 1: Table S11)[13].

Supplementary Tables

	Sequencer	Ethnicity	Disease	Median length	Mean length	number of reads	total base	exprected coverage
Patient1	PromethION	Caucasian	Primary ovarian failure	13,104	12,957.9	8,642,604	111,990,048,861	37
Patient2	PromethION	Japanese	Primary ovarian failure	3251	6,837.4	17,131,141	117,132,669,422	39
Patient3	PromethION	Japanese	Bilateral split-foot malformation	3223	6,333.2	14,926,358	94,531,709,149	32
Patient4	PromethION	Japanese	West syndrome	2334	6,004.3	6,845,364	41,101,961,286	14
Control1	PromethION	Japanese	Epilepsy	17,218	19,058.7	5,074,319	96,709,785,254	32
Control2	PromethION	Japanese	Unaffected family control (father of control1)	18,142	20,203.6	4,497,556	90,866,959,081	30
Control3	PromethION	Japanese	Unaffected family control (mother of control1)	16,986	18,521.5	4,403,236	81,554,440,718	27
Control4	PromethION	Japanese	Neuronal Intranuclear Inclusion disease	1,558	4,477.8	12,830,261	57,451,863,375	19
Control5	PromethION	Japanese	Unaffected family control	2,452	3,632.9	9,635,261	35,004,315,947	12
Control6	PromethION	Japanese	Unaffected family control	691	2,340.2	23,303,818	54,535,886,940	18
Control7	PromethION	Japanese	Renal hypoplasia	7,127	9,953.0	7,824,636	77,878,925,289	26
Control8	PromethION	Japanese	WEST syndrome	14,670	15,094.4	3,557,359	53,696,135,713	18
Control9	PromethION	Japanese	Neuronal Intranuclear Inclusion disease	3,520	5,203.2	15,926,839	82,870,233,437	28
Control10	PromethION	Japanese	Neuronal Intranuclear Inclusion disease	3,298	5,466.3	10,589,493	57,885,853,963	19
Control11	PromethION	Japanese	Neuronal Intranuclear Inclusion disease	2,990	5,598.3	11,109,622	62,195,375,599	21
Control12	PromethION	Japanese	Neuronal Intranuclear Inclusion disease	3,736	6,820.9	10,658,181	72,698,332,750	24
Control13	PromethION	Japanese	Neuronal Intranuclear Inclusion disease	3,169	7,012.2	5,529,417	38,773,128,508	13
Control14	PromethION	Japanese	Unaffected family control	5,889	7,025.5	9,091,759	63,874,106,363	21
Control15	PromethION	Japanese	Neuronal Intranuclear Inclusion disease	1,608	2,773.6	17,969,232	49,838,565,927	17
Control16	PromethION	Japanese	Neuronal Intranuclear Inclusion disease	3,520	8,162.9	6,786,707	55,398,873,781	18
Control17	PromethION	Japanese	Neuronal Intranuclear Inclusion disease	3,110	4,902.2	11,467,114	56,214,013,607	19
Control18	PromethION	Japanese	Neuronal Intranuclear Inclusion disease	3,861	5,785.1	13,655,084	78,995,795,042	26
Control19	PromethION	Japanese	Focal cortical dysplagia	3,425	5,036.7	17,761,209	89,558,068,739	30
Control20	PromethION	Japanese	Epilepsy	3,392	6,540.9	10,500,704	68,684,084,437	23
Control21	PromethION	Japanese	Epilepsy	3,382	6,645.8	9,582,128	63,680,708,046	21
Control22	PromethION	Japanese	Epilepsy	2,645	5,105.2	12,998,831	66,361,384,504	22
Control23	PromethION	Japanese	Epilepsy	2,035	4,135.6	20,502,426	84,790,477,596	28
Control24	PromethION	Japanese	Brain abnormality	3,893	7,265.3	17,229,947	125,180,249,687	42
Control25	PromethION	Japanese	Cerebellar ataxia	3,601	6,305.7	17,880,435	112,748,995,082	38
Control26	PromethION	Japanese	Epilepsy	2,744	6,002.9	16,280,893	97,732,025,016	33
Control27	PromethION	Japanese	Epilepsy	1,719	3,774.3	23,432,032	88,440,096,328	29
Control28	PromethION	Japanese	Epilepsy	20,468	22,153.9	2,313,819	51,571,485,654	17
Control29	PromethION	Japanese	Benign adult familiay myoclonic epilepsy	3,087	4,884.0	8,709,727	42,536,118,154	14
Control30	PromethION	Japanese	Benign adult familiay myoclonic epilepsy	2,126	3,295.0	13,143,397	43,307,793,923	14
Control31	PromethION	Japanese	Benign adult familiay myoclonic epilepsy	13,749	15,332.0	2,515,618	38,569,602,139	13
Control32	PromethION	Japanese	Benign adult familiay myoclonic epilepsy	12,248	13,319.0	3,795,124	50,547,125,919	17
Control33	PromethION	Caucasian	Ablepharon and macrostomia	9,606	9,072.5	10,503,537	95,293,663,017	32

Table S1. Summary of PromethION sequencing data.

		group	Forward	Reverse	
**Patient**	**breakpoint PCR**				
**Patient 1**	der-chr2	3	gggcaacaatttccacatctgaa	tcccctgtgatggacttaacaag	Fig S7b
	der-chrX	9	gttttggaagtcttggttcccag	gcattagtctttgcacctgtgag	Fig S7b
**Patient 2**	der-chr4	2	tccctagagaatccccaagtc	ttgcctactcttactcttcagcc	Fig S10b,c
	der-chrX	5	tgcctttcatgataagctctgg	gaatgttcttgaggcctttgc	Fig S10b,c
	chrX del	10	ccacaatttggtgagtgtccttac	tgagactcaattccaattgtaggc	Fig S10b,c
	complex chr11-1	6	cttattccctctcatagatgcac	ggaaacatcttcagacagaaactag	Fig S12a,b
	complex chr11-2	6	ccaattcagtgaggaaagtcattg	gaacatttgggagtttattgaggtc	Fig S12a,b
	complex chr11-3	12	ctaaaattcagcagcgttatcaaat	gaaaggacaaaaagagtttatgcaa	Fig S12a,b
	complex chr11-4	16	tgtgctgtcttactggtaagtgtta	ccgatgacaattagacattcttggg	Fig S12a,b
**Patient 3**	der-chr7	18	caggaaataagagactggttcctaa	catgttactgcagatgatgagattt	Fig S15a
	der-chr15	17	tgattagctctgtacctgaggactt	aaaggattacattgtgatgcaaact	Fig S15b
		13	ttccagagcgattgtattatttagc	tcaatggcagtactagattcacaac	Fig S15c
		1	gtttaatttaagcgtcccacctaat	tgaaacaagccgtatgtatgatatg	Fig S15d-1
		1	aactctctcatcacatggttcattt	actaacaagcagggatacaaacaag	Fig S15d-2
		5	agaattggaaaagagatcttgtgtg	aaccctgtaaatgtggaattctgta	Fig S15e-1
		5	agtgaattccattcagtgtaccatt	gatatagcaggcatcctattttgtg	Fig S15e-2
		5	ccatagatggatacatggataaaca	actcagacttgtaggtaaccccatt	Fig S15e-3
		12	tatagcttgaaggaattccatttgt	gctatccagagcatggtctattcta	Fig S15f
		35	atagtagctgcttgtgcctgtaatc	ggtgatggttatggttatcttctca	Fig S15g
		3	accagagaattgaagatgactatgg	aattgtgggaggtaattatccattt	Fig S15h
	der-chr9	21	agctagacctgcaatagcaaactta	gcatttagaaaccattcctgtagaa	Fig S15i
		33	atttgtgaactctgacttgaggaag	ataactttcttataatgtattaaccatgctgtag	Fig S15j
		11	ctgcatggaagtaggggtgt	acactggcccacaaaaagag	Fig S15k
		30	ccagtctaggacctctcttagtacttttat	cccaactctctaatgttctactaccttact	Fig S15l
		22	ggtttcttatatattctggttattaattctttgt	ctagtccttcccaacacattagttttat	Fig S15m
		21	ggaaggcaaattcaatccaa	acctggctcacaaggtatgg	Fig S15n
	der-chr14	6	cctgacctagaactgtcccactaag	accaagatgtttctacaaaaagcac	Fig S15o
	**AluYb5 PCR**				
**Patient 1**		51	ctgtgaacaaccctagtcttttgg	gctgccctcagaagaataaatg	Fig S9
		65	tcctttaataccaacactgcacctc	cttcaaattgttcctcaggggattc	Fig S9
		72	ttcaggtctctcagcccagcatc	ggatccctttccagtaacagcacc	Fig S9
	**QPCR**				
**Patient 3**	*SEM1*		cagttacgagctgaactagagaaa	gggttctctgccctagaatgt	

Table S2. Primers.

Primers used to confirm breakpoints and qPCR in Patient1, 2 and 3.

	Patient1	Patient2	Patient3	Patient4
Original	2,773	3,336	3,351	2,523
data1	1,392	2,858	2,075	1,302
data2	794	1,860	1,206	806
data3	539	1,257	804	570
data4	409	609	428	292
data5	367	459	316	225
data6	307	335	234	140
data7	267	262	186	106
data8	246	228	160	98
data9	218	182	136	79
data10	204	159	120	66
data11	194	147	112	58
data12	181	122	103	51
data13	171	111	96	47
data14	164	105	92	45
data15	162	99	87	39
data16	160	93	82	36
data17	156	89	79	36
data18	151	82	77	35
data19	144	74	70	32
data20	141	71	67	32
data21	141	68	66	30
data22	137	66	63	29
data23	133	61	62	29
data24	129	54	59	29
data25	125	52	54	28
data26	124	48	51	27
data27	122	48	50	24
data28	121	46	48	22
data29	119	46	47	22
data30	119	45	47	21
data31	118	42	47	21
data32	117	41	47	21
data33	101	37	46	21
with -c1 option	80	33	43	14

Table S3. Number of groups of rearranged reads in each patient, after successive filtering using 33 control humans. Original: number of groups in each patient before filtering.

	Patient1	Patient2	Patient3	Patient4	other structural variation tools for Patient3	
					sniffles	NanoSV
real	205m48.036s	234m9.006s	154m17.363s	134m53.602s	198m40.757s	5361m3.771s
user	202m43.690s	232m40.968s	153m2.223s	133m49.292s	420m38.719s	3446m56.912s
sys	2m51.771s	1m30.713s	1m19.762s	1m5.775s	2m6.462s	2627m25.827s

Table S4. Computational time usage for grouping rearranged reads from the patient and subsequent filtering using 33 controls, and other SV detection tools. real: wall clock time. user: CPU time. sys: CPU time within the system.

	alignment to GRCh38			
	NGMLR	**last-train (v1060)**	**LAST (v1060) -P16**	minimap2
real	2739m1.616s	41m24.576s	1234m29.371s	693m22.452s
user	43091m36.694s	43m39.373s	10368m58.192s	2102m22.897s
sys	397m45.575s	1m4.917s	10m7.432s	10m56.283s
Max resident set size(kbytes)	76,358,704	11,662,304	12,015,872	47,371,760

Table S5. Computational time usage of LAST and other aligners. real: wall clock time. user: CPU time. sys: CPU time within the system.

	Patient1	Patient2	Patient3	Patient4
tandem multiplication	15	3	7	3
tandem repeat expansion	13	6	3	1
retrotransposition	16	6	3	1
non-tandem duplication (insertion)	9	4	3	0
non-tandem duplication with target site deletion	3	0	0	0
large tandem duplication	1	3	1	3
deletion	8	1	5	2
inversion	1	0	1	1
processed pseudogene insertion	0	1	1	1
NUMT	1	2	0	0
unclear	7	2	4	2
chromosomal translocation	2	2	0	0
possible inversion duplication	2	0	0	0
complex rearrangement	0	3	15	0

Table S6. Number of patient-only rearrangements in each category. NUMT: nuclear mitochondrial DNA insertions.

chr	Fig. S12	Sequnce in between breakpoints
der(7)	a	G overlap
der(15)	b	AT microhomology
	c	AG microhomology
	d-1	blunt
	d-2	T insertion
	e-1	C overlap
	e-2	A insertion
	e-3	TTA microhomology
	f	blunt
	g	TC microhomology
	h	blunt
der(9)	i	blunt
	j	blunt
	k	ACTTCAGG insertion
	l	blunt
	m	blunt
der(4)	n	blunt
der(14)	o	T overlap

Table S7. Sequence features of 18 breakpoints in Patient 3.

Most of the breakpoints show blunt end or microhomology-mediated ligation.

		NanoSV calls				
Examples	types of rearrangement	chr	position	ref	alt	SV length
Patient 1 (Fig. 4e)	AluYa5 insertion	chr1	3211404	T	<INS>	287
	AluYb8 insertion	chr12	58987676	A	<INS>	288
		chr12	58987679	A	]chr2:18580627]A	
	L1HS insertion	chr2	198915190	T	T[chr4:149915528[	
	ERVK insertion	chr12	123581929	A	]chr15:58834291]A	
Patient 3 (Fig. 6g)	Processed pseudogene/AluYa5 insertion	chr15	93296473	T	[chr2:227325268[T	
		chr15	93296485	T	T]chr2:227357830]	
		chr15	93292543	A	<INS>	235
		chr2	227210436	A	A[chr2:227210535[	
		chr2	227325428	G	G[chr2:227328680[	
		chr2	227328788	T	T[chr2:227329606[	
		chr2	227329782	A	A[chr2:227330626[	
		chr2	227352569	G	<DEL>	3105
		chr2	227355757	G	<DEL>	1227

Table S8. Examples of NanoSV calls for the TE-insertions of Patient 1 and processed pseudogene-AluYa5 insertion in Patient 3. AluYa5 insertion in Patient 3 was not detected.

# in Fig. S19	type	Inheritance
group1	TE-insertion (SINE-MIR)	paternal
group2	inversion	maternal
group3	(TA)n insertion	paternal
group4	tandem duplication	paternal
group5	TE insertion (SVA_F)	maternal
group6	inversion and deletion	paternal
group7	deletion	paternal
group8	deletion	maternal
group9	deletion	maternal
group10	TE insertion (L1HS 3'end)	maternal
group11	TE insertion (SVA_E)	maternal
group12	tandem duplication	maternal
group13	tandem duplication	paternal
group14	tandem duplication	maternal
group15	tandem duplication	paternal
group16	deletion	paternal
group17	tandem duplication?	maternal
group18	deletion	paternal
group19	TE insertion (L1HS 5'end)	paternal
group20	tandem multiplication	maternal
group21	TE insertion (SVA_D)	paternal
group22	tandem duplication	maternal
group23	TE insertion (SVA_E)	maternal
group24	TE insertion (L1HS)	maternal
group25	TE insertion (L1HS)	maternal
group26	tandem multiplication?	paternal
group27	TE insertion (L1HS_3'end)	maternal

Table S9. Trio analysis shows filtered rearrangements in child are inherited from either of the parents.

	Reported SV				dnarrange results					difference		
picture# in Fig. S20	Chr	start	end	Length		chr	start	end	Length	start	end	
1	chr1	65,558,490	65,564,584	6,094	detected	chr1	65558490	65564584	6,094	0	0	
2	chr1	180,780,633	180,786,257	5,624	detected	chr1	180780632	180,786,257	5,625	1	0	
3	chr2	4,165,476	4,175,889	10,413	detected	chr2	4,165,086	4,175,888	10,802	390	1	166bp insertion at the deletion (chr2:4157844-4157678)
4	chr2	14,564,046	14,569,993	5,947	detected	chr2	14564045	14,569,992	5,947	1	1	
5	chr2	109,073,445	109,078,759	5,314	detected	chr2	109073444	109,078,758	5,314	1	1	
6	chr2	129,484,672	129,493,836	9,164	detected	chr2	129484671	129,493,835	9,164	1	1	
7	chr2	150,174,623	150,181,730	7,107	detected	chr2	150174619	150,181,732	7,113	4	-2	
8	chr2	151,580,279	151,590,829	10,550	may not be a simple deletion	-	-	-	-	-	-	7665bp tandem duplication?? (chr2:151590694-151598359)
9	chr2	154,962,898	154,973,674	10,776	detected	chr2	154,962,470	154,973,695	11,225	428	-21	171bp inversion insertion at the deletion (chr2:154968628-154968457)
10	chr2	183,220,794	183,226,219	5,425	detected	chr2	183220793	183,226,218	5,425	1	1	
11	chr2	203,316,940	203,327,011	10,071	detected	chr2	203,316,939	203,327,010	10,071	1	1	
12	chr6	26,746,456	26,768,434	21,978	detected	chr6	26,746,058	26,768,413	22,355	398	21	399bp insertion at deletion site (chr6:26746457-26746058)
13	chr6	31,984,683	31,991,050	6,367	detected	chr6	31984683	31,991,051	6,368	0	-1	
14	chr7	12,982,477	12,988,926	6,449	detected	chr7	12982476	12,988,925	6,449	1	1	
15	chr7	39,780,499	39,785,603	5,104	detected	chr7	39,780,498	39,785,602	5,104	1	1	
16	chr8	7,564,985	7,572,630	7,645	may not be a simple deletion	-	-	-	-	-	-	
17	chr8	40,028,172	40,033,330	5,158	detected	chr8	40,028,170	40,033,335	5,165	2	-5	
18	chr9	66,061,642	66,070,788	9,146	may not be a simple deletion	-	-	-	-	-	-	
19	chr10	51,443,915	51,454,456	10,541	detected	chr10	51,443,916	51,454,462	10,546	-1	-6	
20	chr11	89,943,626	89,954,393	10,767	may not be a simple deletion	-	-	-	-	-	-	
21	chr11	134,732,085	134,737,768	5,683	detected	chr11	134,732,084	134,737,767	5,683	1	1	
22	chr12	183,063	188,800	5,737	detected	chr12	183062	188,799	5,737	1	1	
23	chr12	268,331	275,664	7,333	detected	chr12	268,330	275,663	7,333	1	1	
24	chr16	19,934,232	19,956,263	22,031	detected	chr16	19,934,228	19,956,257	22,029	4	6	
25	chr16	62,510,429	62,516,759	6,330	detected	chr16	62,510,425	62,516,764	6,339	4	-5	
26	chr18	32,915,724	32,921,292	5,568	detected	chr18	32,915,724	32,921,292	5,568	0	0	
27	chr19	46,119,478	46,125,056	5,578	detected	chr19	46,119,477	46,125,055	5,578	1	1	
28	chr22	22,901,309	22,906,676	5,367	may not be a simple deletion	-	-	-	-	-	-	
29	chrX	49,583,493	49,593,015	9,522	may not be a simple deletion	-	-	-	-	-	-	
30	chrX	56,771,538	56,776,939	5,401	different deletion?	chrX	56,775,380	56,780,808	5,428	-3842	-3869	

Table S10. Comparison to reported SVs in NA12878.

We checked large deletions (more than 5 kb) in one human genome (NA12878) that were reported previously. To detect deletions > 5kb, we added -g5000 option to `dnarrange`. Our pipeline without control filtering found rearrangements at the sites of all 30 reported deletions, but with further complexity in some cases.

Patient	insert locus	insert length	strand	mt gene	mismap probability	insertion locus	insertion locus gene	RepeatMasker annotation	Near-by A-T sequence (<20bp)
Patient1	chrM:15065-151	36	+	CYB	mismap=1.39e-05	chr2:213419058-213419060	SPAG16 intron	LINE L1	AATAAAA
Patient2	chrM:10556-106	126	-	ND4L	mismap=1e-10	chr14:80547592-80547596	CEP128 intron	LINE L2	TAAAAT
Patient2	chrM:11680-117	64	+	ND4	mismap=0.5	chr4:7252649-7252650	SORCS2 intron	DNA/hAT-Blackjack	AATT

Table S11 NUMT origin and insertion site.

Nuclear Mitochondrial sequences (NUMT) found in Patient 1 and Patient2.

Table S12 is shown in a separate file (Additional file 2).

Table S12. Detailed description of patient-only rearrangements in Fig. S3, S6, S11 and S14.

Group25 in Patient1 is an L1HS insertion, which has a 3'-transduction indicating that the source is a specific L1HS in chr4. This chr4 L1HS is absent in the reference genome, but is present in some humans (hg18.chr4:129184647) [14]. For group 24 & 52, inverted duplication in chr16 and chr20, are also present in public nanopore data NA12878 (rel3 and rel4. Jain et al.2018, detected by our analysis) [15], suggesting that a reported duplication (array CGH) or inversion (WGS) near this locus (International HapMap, C. et al. 2010, Genomes Project, C. et al. 2010) [16] [17] in NA12878, is actually identical to this inverted duplication. For group6, 12 and 16 in Patient2, dnarrange-link infers a chr11 complex rearrangement (Figure 5e).

Table S13 is shown in a separate file (Additional file 3).

Table S13. Comparison of the breakpoints detected by dnarrange, ngmlr-Sniffles and LAST-NanoSV to Sanger sequence-confirmed breakpoints.

Breakpoints were described according to vcf (variant call format) version 4.2 (https://cseweb.ucsd.edu/classes/sp16/cse182-a/notes/VCFv4.2.pdf). Note that in ngmlr-Sniffles and LAST-NanoSV, we only look for translocation sites (suggested by G-banded chromosomal analysis) because there is no method to filter out common/benign changes present in controls.

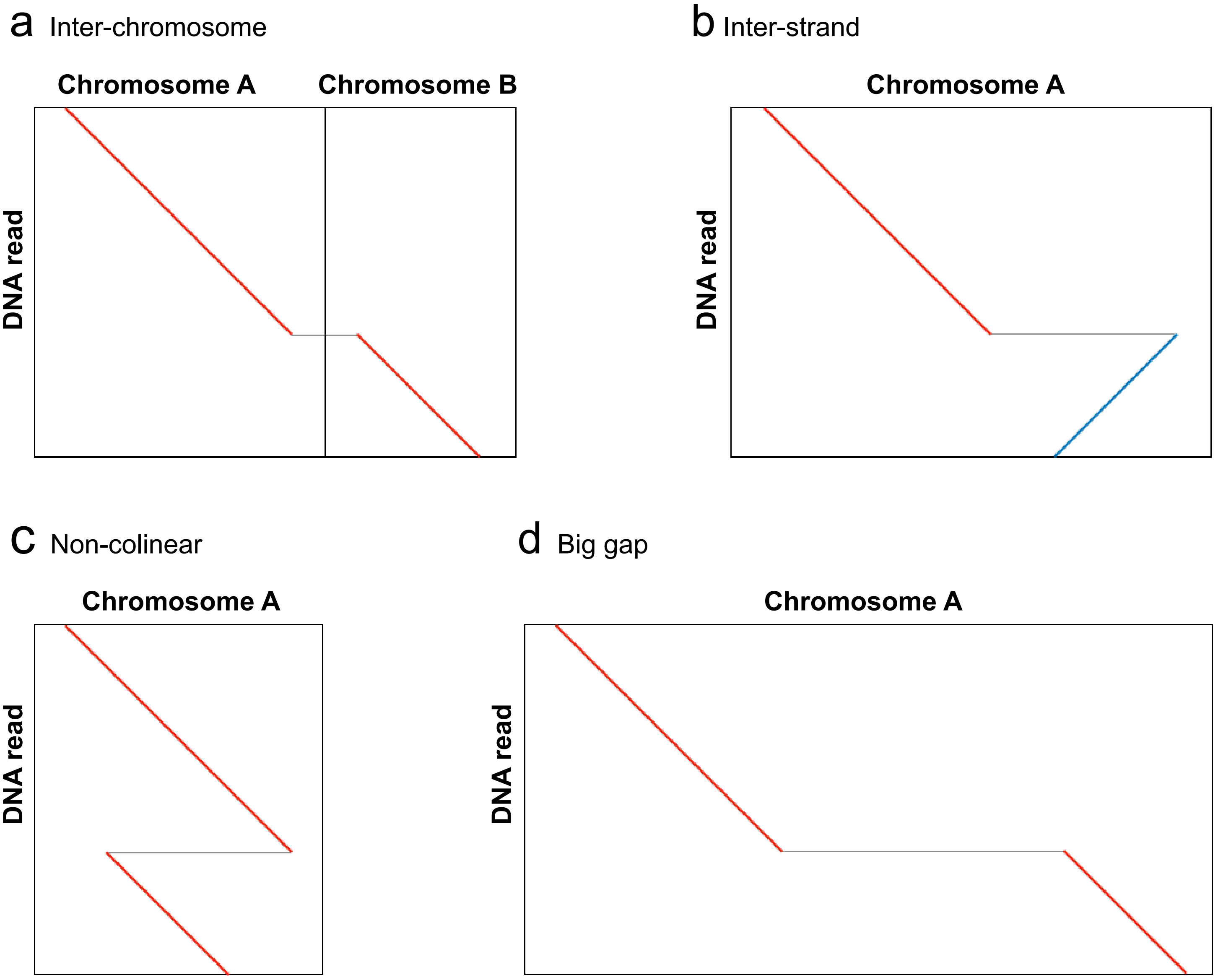

Fig S1.
The four types of rearrangement. Note this is not a classification of whole rearrangement phenomena (e.g. gene conversion, processed pseudogene insertion). This is a classification of minimal rearrangements, which could be parts of larger wholes.

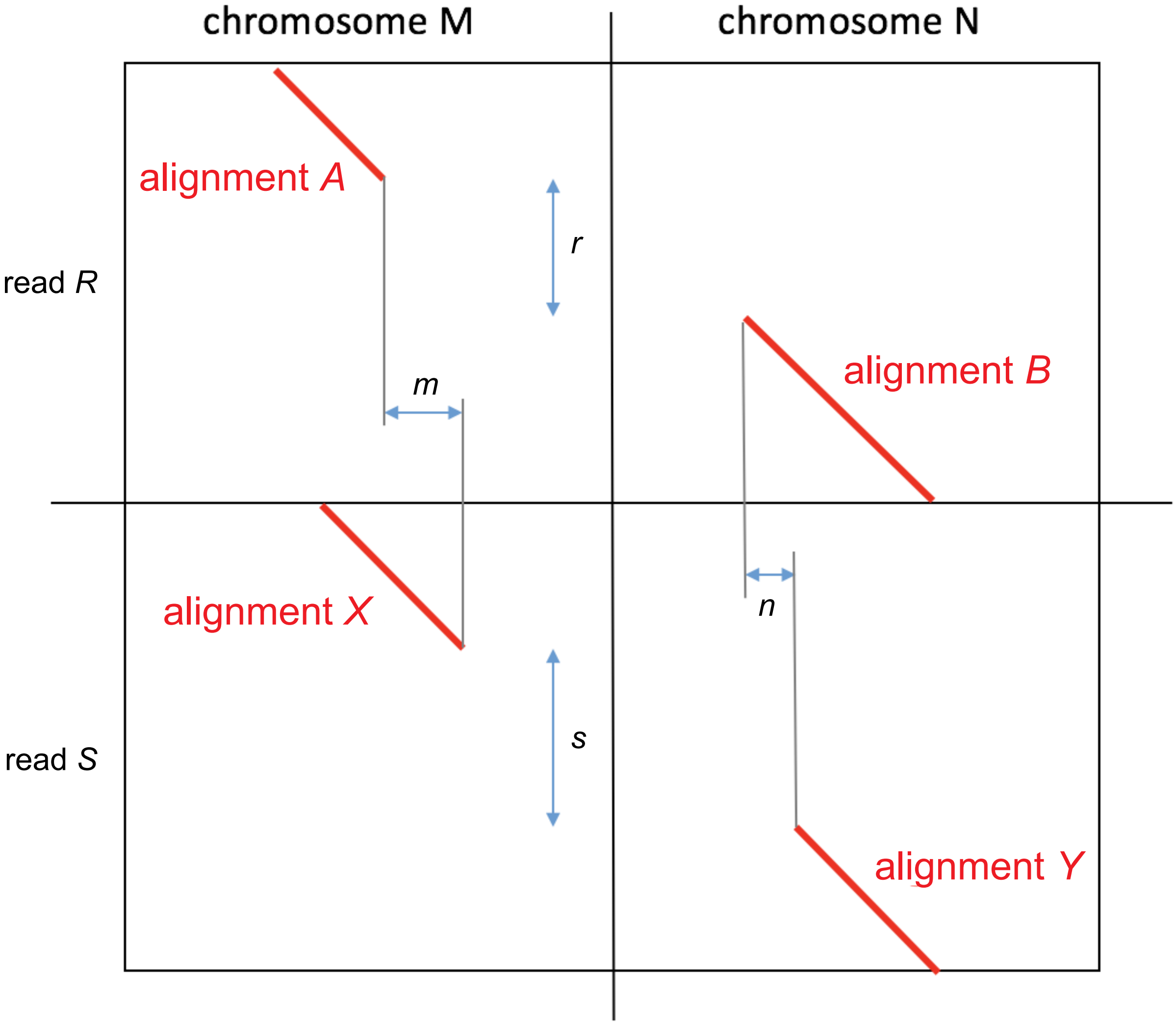

Fig S2. Sketch of the information considered by dnarrange to judge whether two reads share a rearrangement.
Note that m, n, r, and s are not absolute values: they may be less than zero. For example, n equals the chromosomal start coordinate of Y minus the chromosomal start coordinate of B. In the example shown here: m, n, r, and s are all greater than zero.

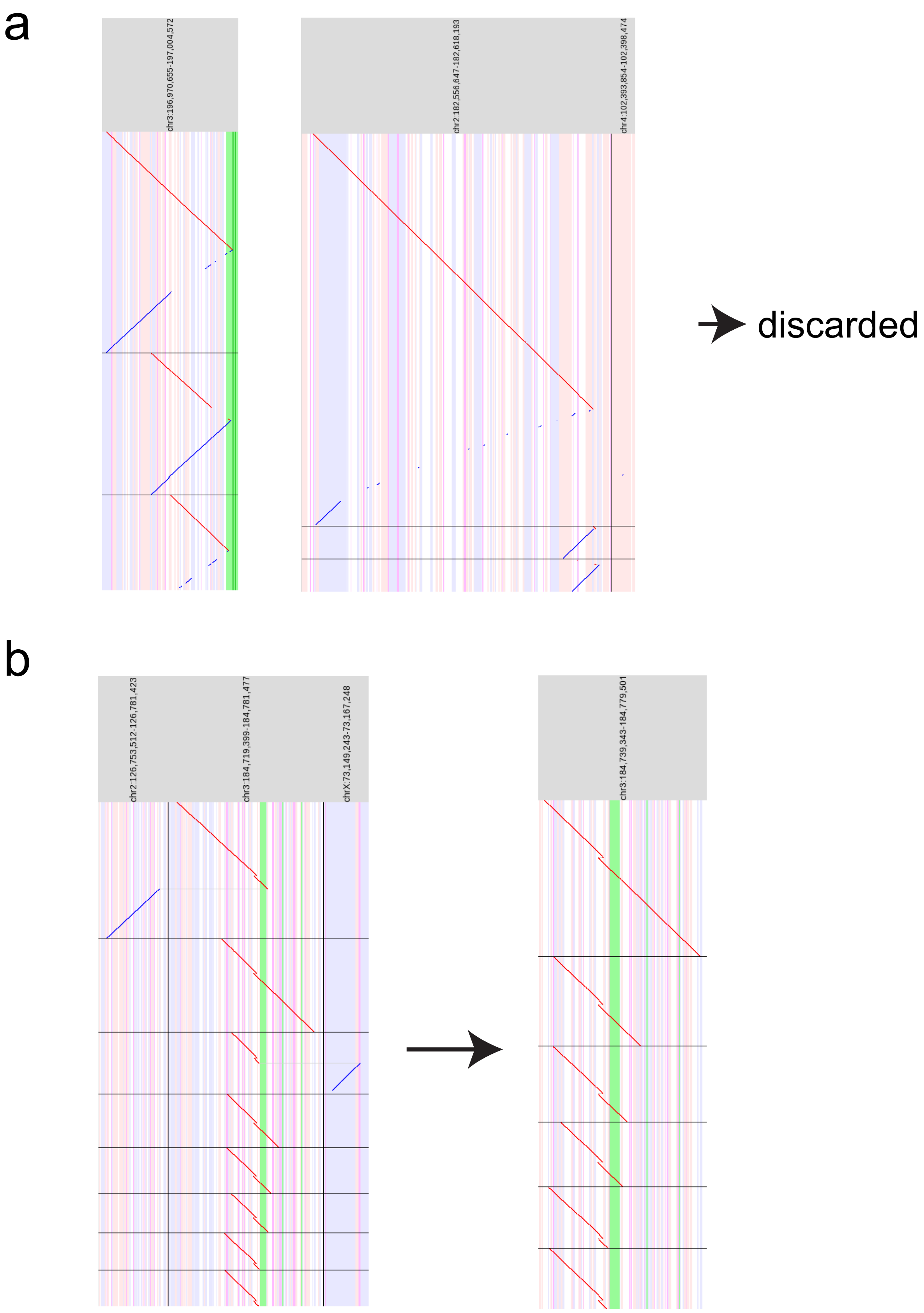

Fig S3. Example of rearrangements filtered by -c1 option.

a. Examples of rearranged reads, where we suspect the rearrangements to be artifacts of the sequencing process, which were excluded by running dnarrange with option –c1 (they are likely to be artifacts because almost the same length of reverse complimentary strand starts from the end of the other strand: we suspect this might be caused by the chimeric reads generated from the other strand of the nanopore DNA library). Three groups of rearranged reads are shown.

b. In this group of rearranged reads, two reads with unique rearrangements were excluded by the –c1 option.

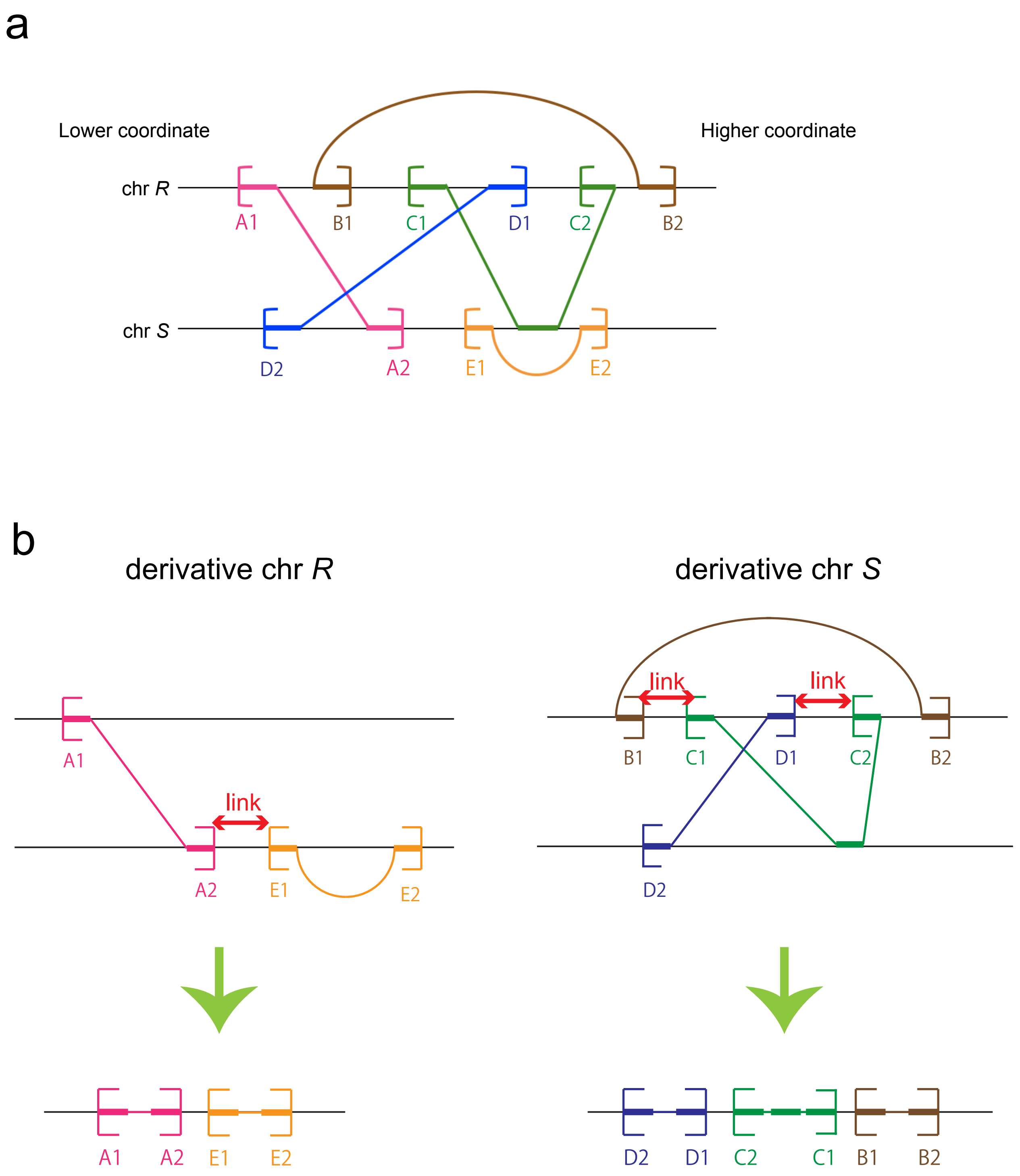

Fig S4. Illustration of data analyzed by dnarrange-link
a. The sketch shows alignment of five DNA reads (A, B, C, D, E) to a genome. The two ends of each read are arbitrarily labeled 1 and 2. b. Derivative chr R was reconstructed by linking A2 to E1 (left). Derivative chr S was reconstructed by linking B1 to C1, and D1 to C2 (right). B1 can also be linked to C2, but in that case it is impossible to link C1 to anything, and D1 to anything, thus this possibility was suppressed.

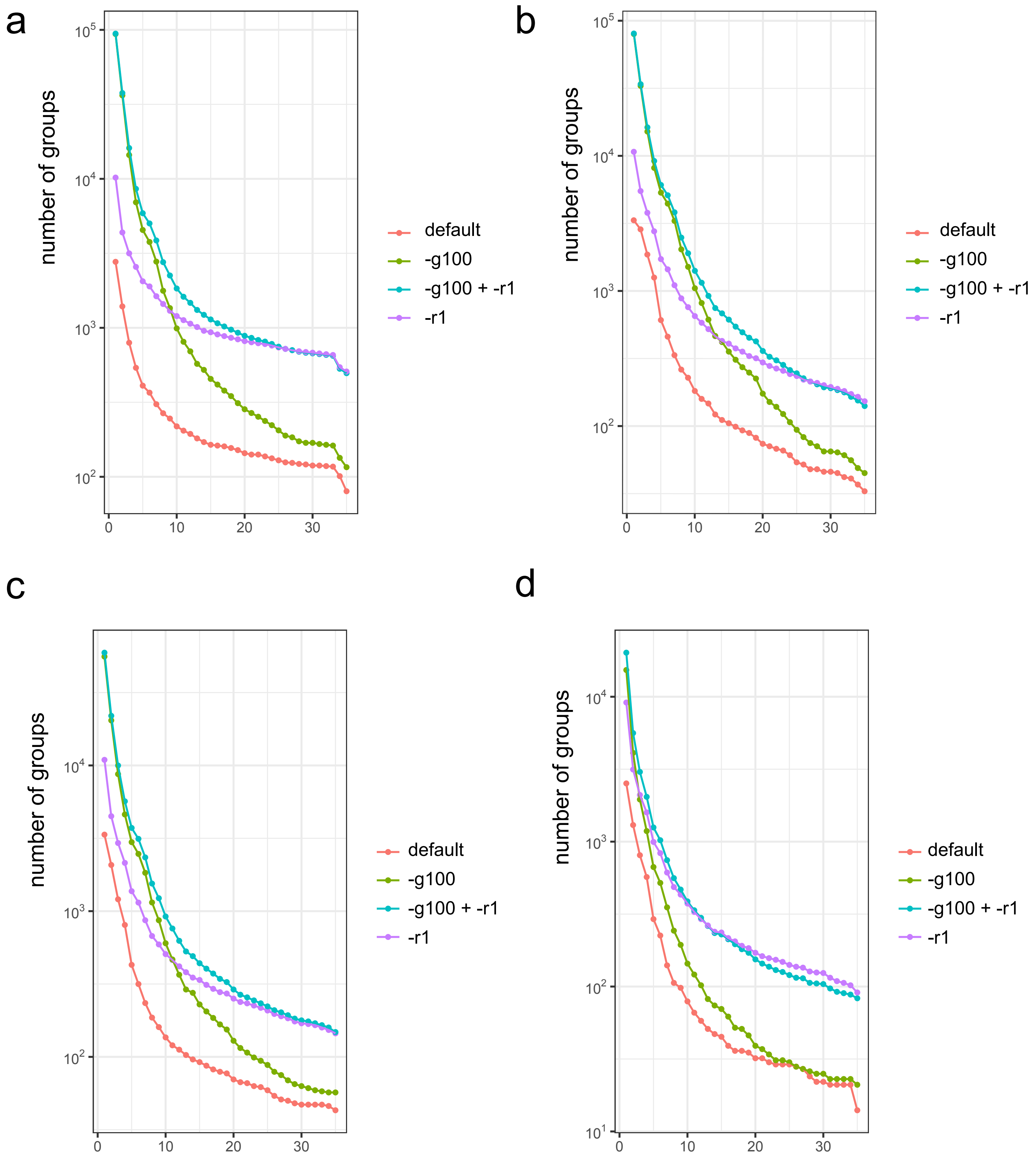

Fig S5. Effect on rearrangement numbers and filtering of changing -g and -r option.
Using -g100 and -r1 options which counts small deletions and duplications detects more rearrangements at first.
By filtering with 33 controls and -c1 option (x-axis), the number of rearrangements decrease exponentially and close to the default (-g10000, -r1000). a. Patient1, b. Patient2, c. Patient3, d. Patient4.

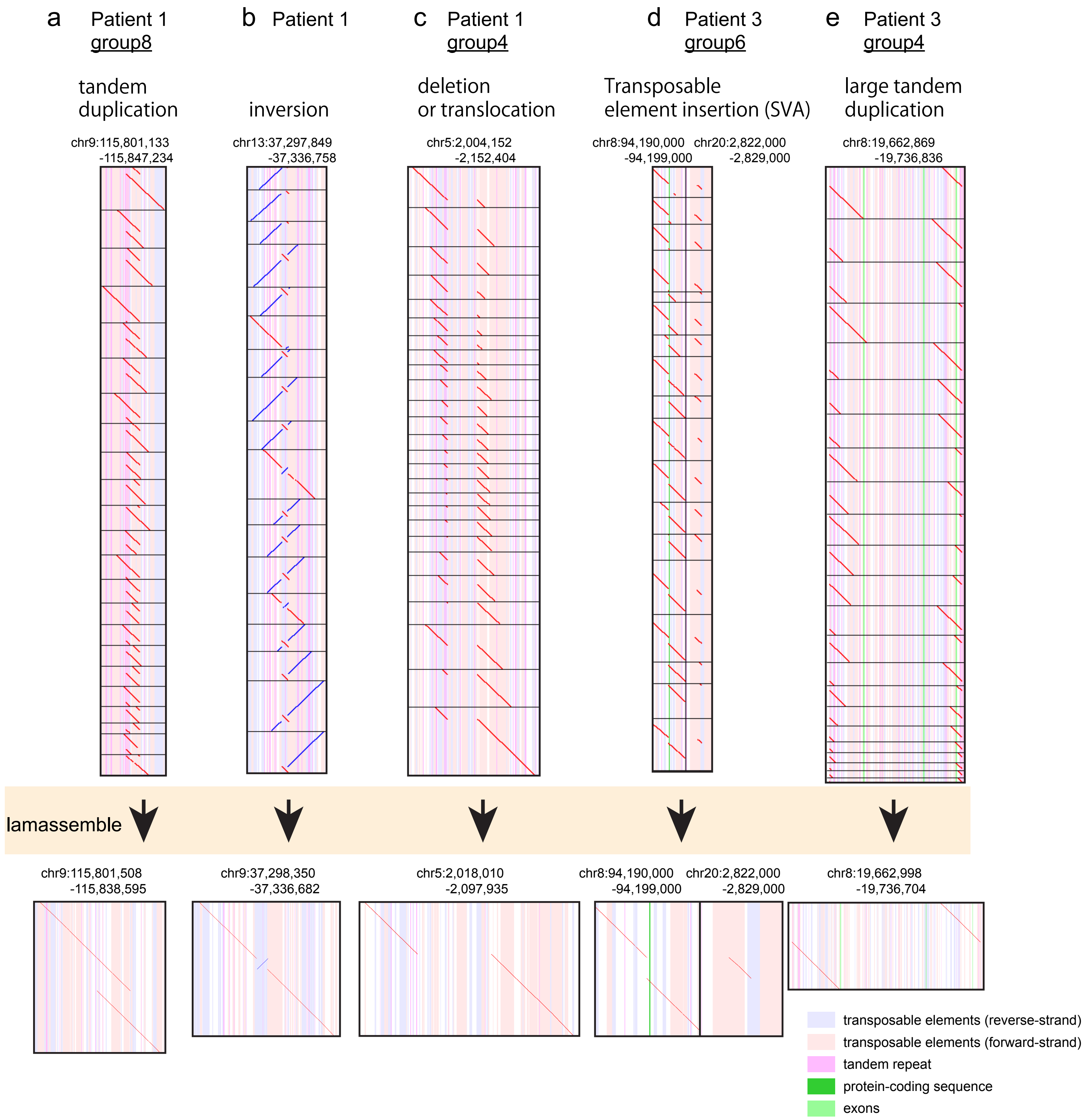

Fig S6. Examples of grouped rearranged reads and consensus sequences.
Dot-plot of examples of grouped rearranged reads. In a dot-plot, a diagonal line is drawn where a read sequence (vertical) aligned to the reference sequence (horizontal). The horizontal black lines are boundaries between different dot-plots showing different DNA reads. a. tandem-multiplication. b. inversion. c. deletion or translocation. d. non-tandem duplication or translocation (insertion). In this dotplot, the inserted sequence is aligned to a transposable element (pale pink) e. possible large tandem duplication. It is not clear if this is a tandem duplication because no one read encompasses the whole duplication. However, this is the simplest interpretation unless other rearrangements are found in the region, thus we categorized this as large duplication.
Examples a-c: Patient 1, d,e. Patient 3. The vertical stripes indicate annotations in the reference genome: tandem repeats (purple), transposable elements (pink:forward-strand, blue:reverse-strand), green (exon) and dark green (protein-coding sequence).
Each group of reads was assembled by lamassemble: the resulting consensus sequences were realigned to the reference genome.

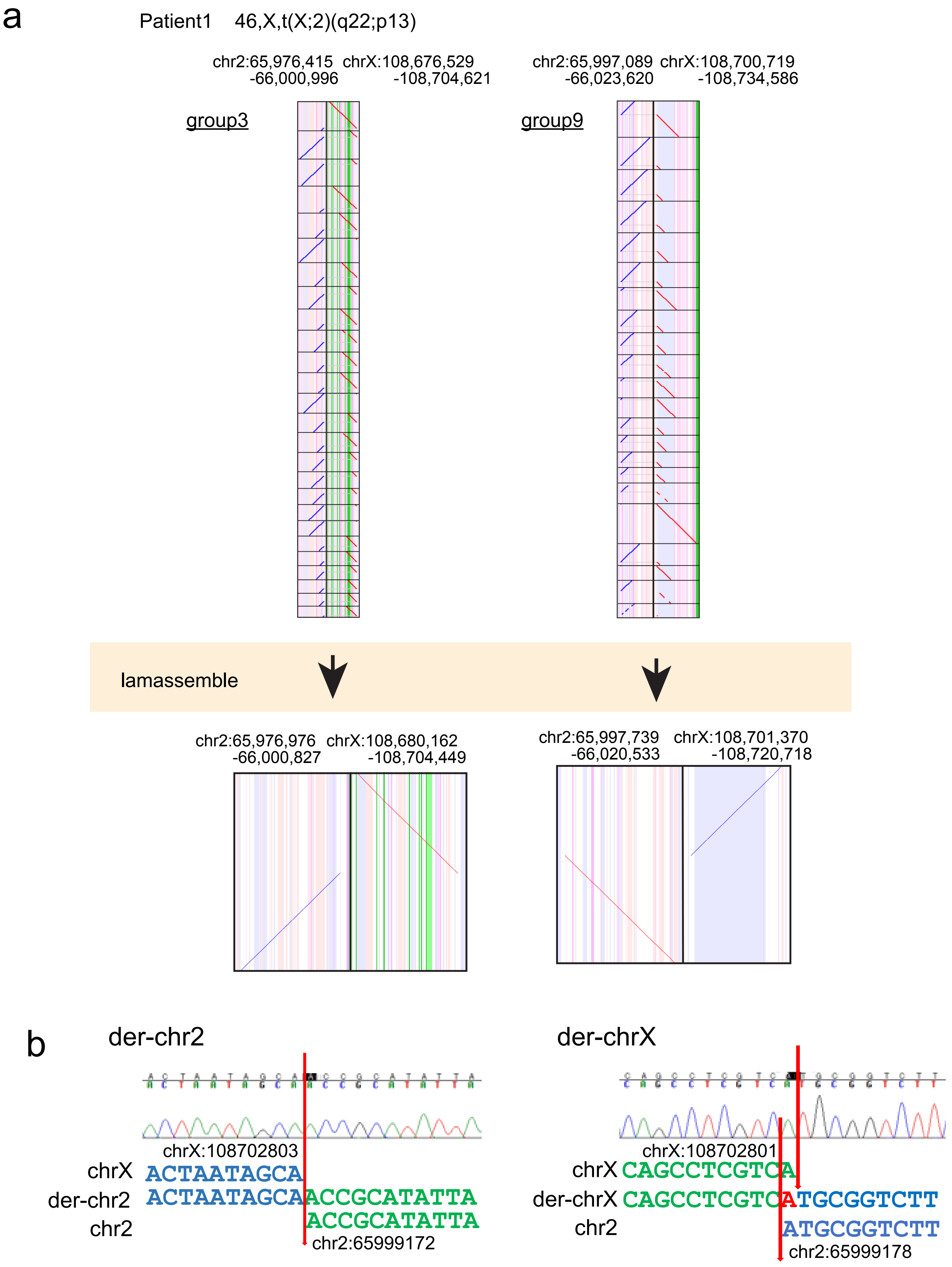

Fig S7. Reciprocal chromosomal translocation in Patient 1.
(a) Dot-plot pictures of grouped rearranged reads and lamassemble consensus sequences at the translocation sites in Patient 1. Translocation t(X;2)(q22;p13) in Patient 1 does not lose any regions nor disrupt any genes. The vertical stripes indicate annotations in the reference genome: tandem repeats (purple), transposable elements (pink:forward-strand, blue:reverse-strand), green (exon) and dark green (protein-coding sequence). The horizontal black lines indicate boundaries between different dot-plots showing different reads. (b) Sanger sequence confirmation of the breakpoints.

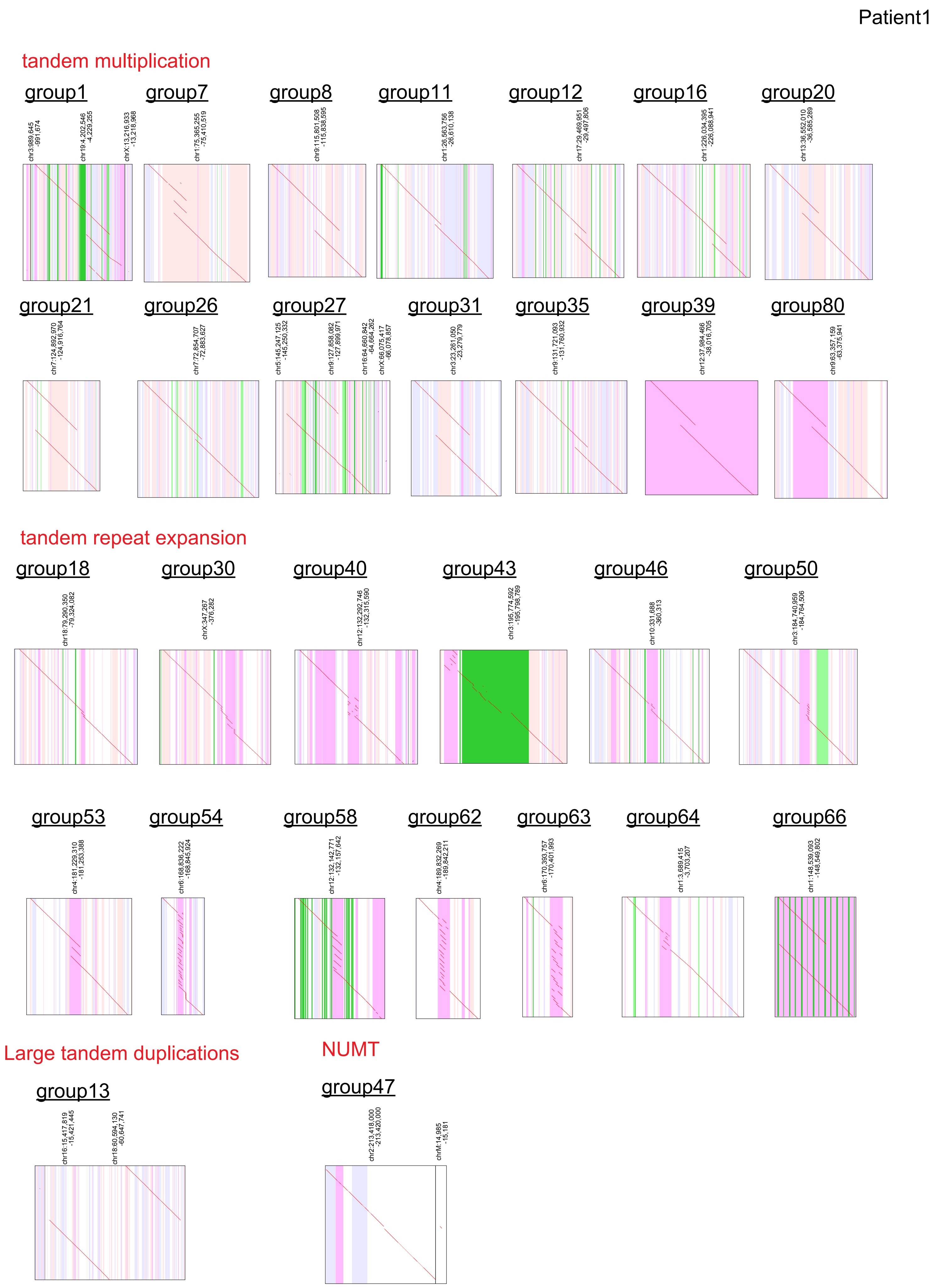

Patient1
tandem multiplication
group1
chr3:989,645 -991,674
chr19:4,202,546 -4,229,255
chrX:13,216,933 -13,218,968
group7
chr1:75,365,255 -75,410,519
group8
chr9:115,801,508 -115,838,595
group11
chr1:26,563,756 -26,610,138
group12
chr17:29,469,951 -29,497,806
group16
chr1:226,034,395 -226,088,941
group20
chr13:36,552,010 -36,585,289
group21
chr7:124,892,970 -124,916,764
group26
chr7:72,854,707 -72,883,627
group27
chr5:145,247,125 -145,250,332
chr9:127,858,082 -127,899,971
chr16:64,660,842 -64,664,262
chrX:66,075,417 -66,078,857
group31
chr3:23,261,050 -23,279,779
group35
chr9:131,721,093 -131,760,932
group39
chr12:37,984,466 -38,016,705
group80
chr9:63,357,159 -63,375,941
tandem repeat expansion
group18
chr18:79,290,350 -79,324,082
group30
chrX:347,267 -376,282
group40
chr12:132,292,746 -132,315,590
group43
chr3:195,774,592 -195,798,789
group46
chr10:331,688 -360,313
group50
chr3:184,740,959 -184,764,506
group53
chr4:181,229,310 -181,253,388
group54
chr6:168,836,222 -168,845,924
group58
chr12:132,142,771 -132,157,642
group62
chr4:189,832,269 -189,842,211
group63
chr6:170,393,757 -170,401,993
group64
chr1:3,689,415 -3,703,207
group66
chr1:148,539,093 -148,549,802
Large tandem duplications
group13
chr16:15,417,819 -15,421,445
chr18:60,594,130 -60,647,741
NUMT
group47
chr2:213,418,000 -213,420,000
chrM:14,985 -15,181

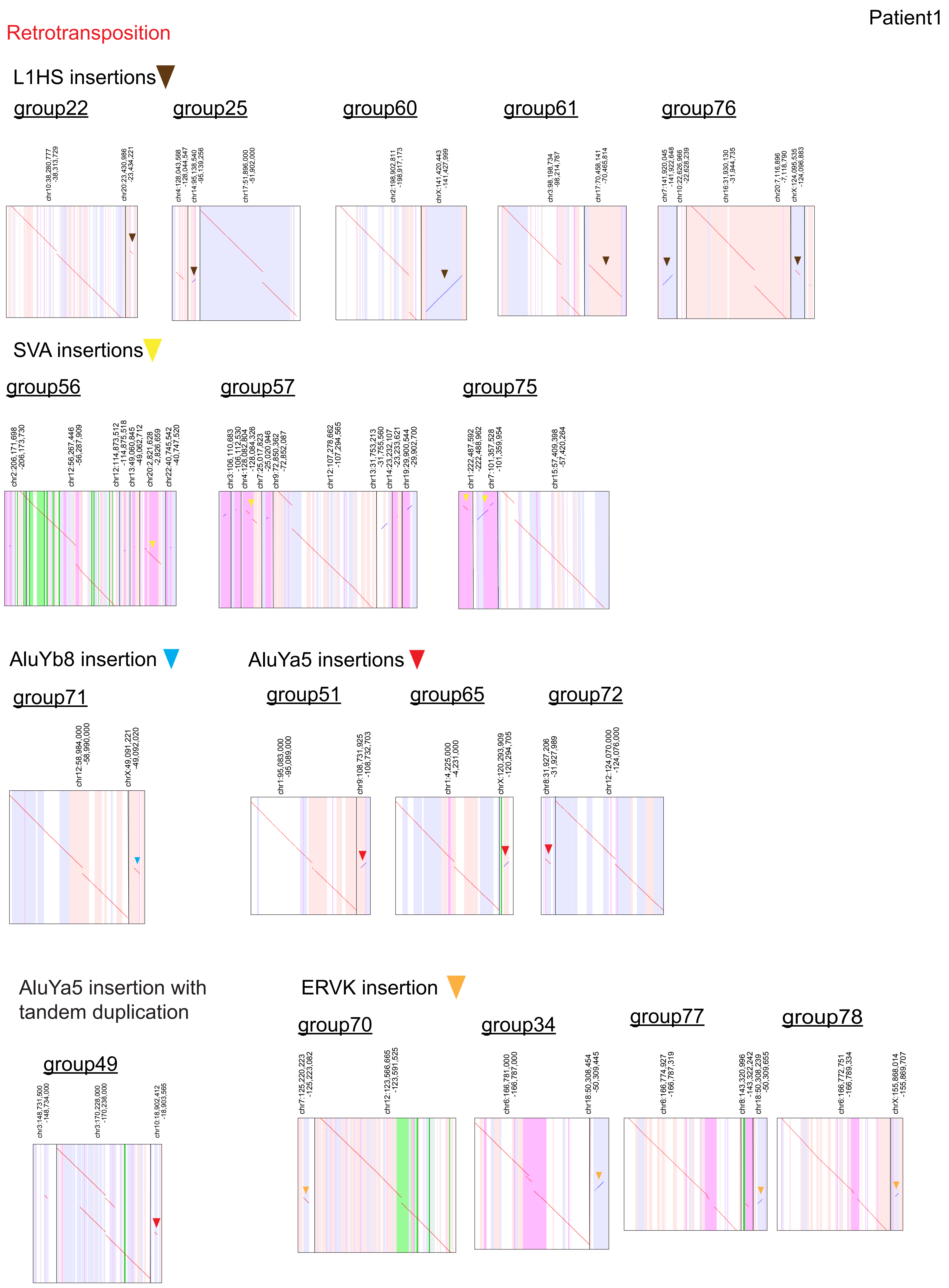
Patient1
Retrotransposition
L1HS insertions
group22
chr10:38,280,777 -38,313,729
chr20:23,430,986 -23,434,221
group25
chr4:128,043,568 -128,044,547
chr14:95,138,540 -95,139,256
chr17:51,896,000 -51,902,000
group60
chr2:198,902,811 -198,917,173
chrX:141,420,443 -141,427,999
group61
chr3:98,198,734 -98,214,787
chr17:70,458,141 -70,465,814
group76
chr7:141,920,045 -141,922,648
chr10:22,626,966 -22,628,239
chr16:31,930,130 -31,944,735
chr20:7,116,896 -7,118,790
chrX:124,095,535 -124,096,883
SVA insertions
group56
chr2:206,171,698 -206,173,730
chr12:56,267,446 -56,287,909
chr12:114,873,512 -114,875,518
chr13:49,060,845 -49,062,712
chr20:2,821,628 -2,826,659
chr22:40,745,542 -40,747,520
group57
chr3:106,110,683 -106,112,530
chr4:128,082,804 -128,084,326
chr7:25,017,823 -25,020,946
chr9:72,850,362 -72,852,087
chr12:107,278,662 -107,294,565
chr13:31,753,213 -31,755,560
chr14:23,232,107 -23,233,621
chr19:29,900,544 -29,902,700
group75
chr1:222,487,592 -222,488,962
chr7:101,357,528 -101,359,954
chr15:57,409,398 -57,420,264
AluYb8 insertion
group71
chr12:58,984,000 -58,990,000
chrX:49,091,221 -49,092,020
AluYa5 insertions
group51
chr1:95,083,000 -95,089,000
chr9:108,731,925 -108,732,703
group65
chr1:4,225,000 -4,231,000
chrX:120,293,909 -120,294,705
group72
chr8:31,927,206 -31,927,989
chr12:124,070,000 -124,076,000
AluYa5 insertion with tandem duplication
group49
chr3:148,731,500 -148,734,000
chr3:170,228,000 -170,238,000
chr10:18,902,412 -18,903,565
ERVK insertion
group70
chr7:125,220,223 -125,223,082
chr12:123,566,665 -123,591,525
group34
chr6:166,781,000 -166,787,000
chr18:50,308,454 -50,309,445
group77
chr6:166,774,927 -166,787,319
chr8:143,320,996 -143,322,242
chr18:50,308,239 -50,309,655
group78
chr6:166,772,751 -166,789,334
chrX:155,868,014 -155,869,707

Patient1

Non-tandem duplication or translocation (insertion)

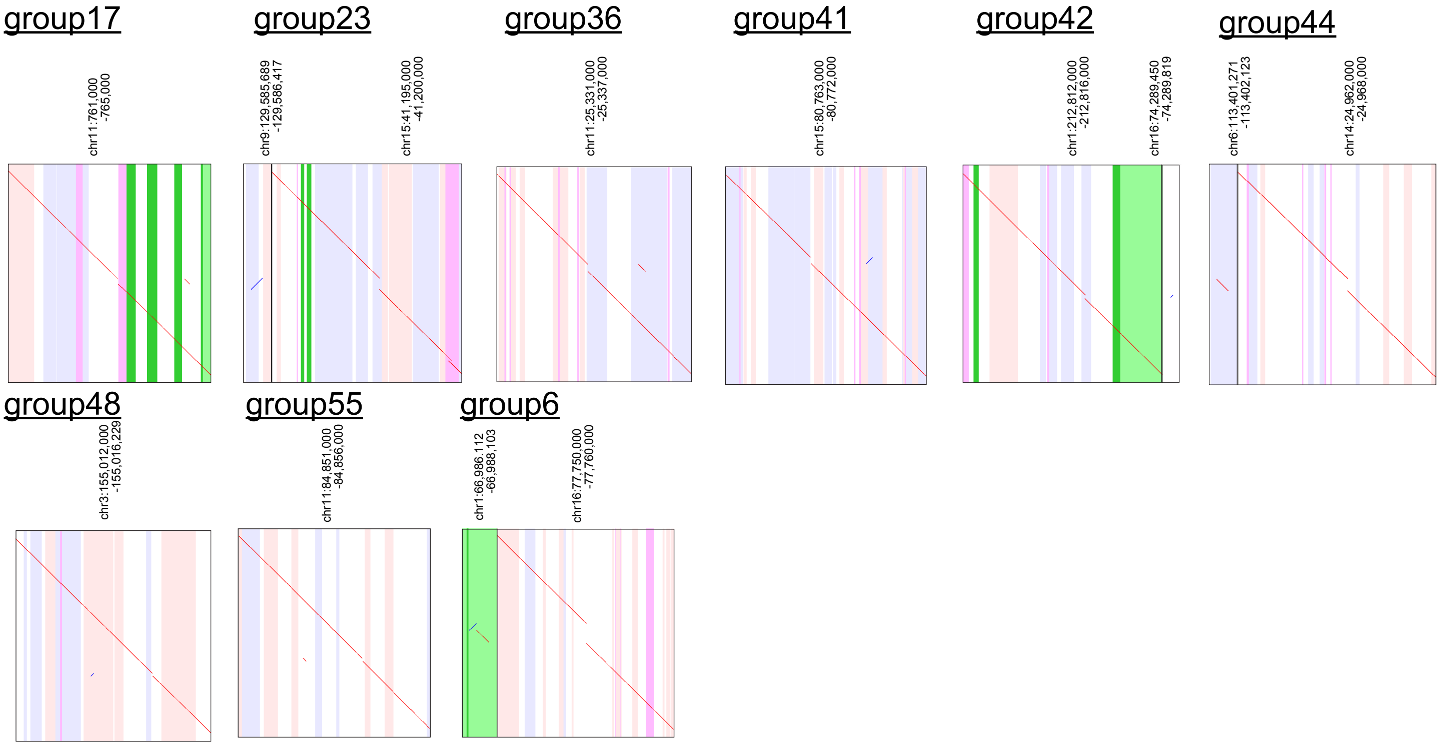

Deletions

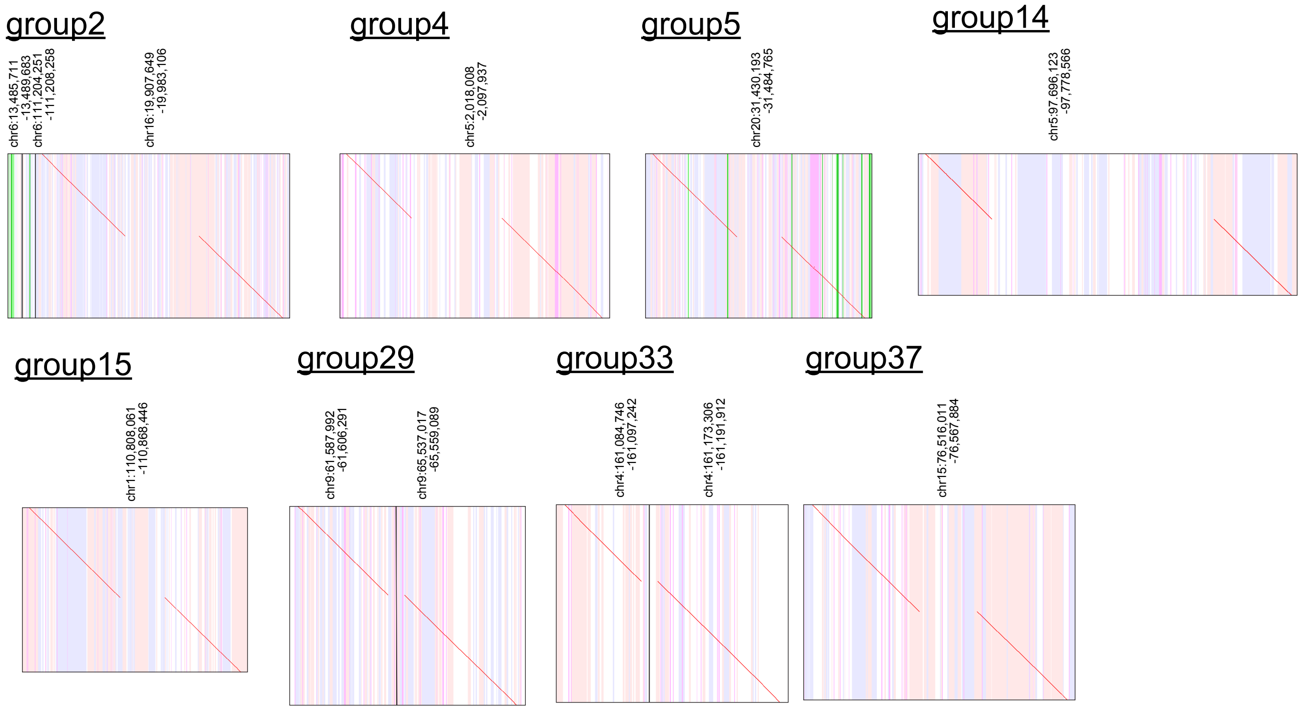

non-tandem duplication with target-site deletion

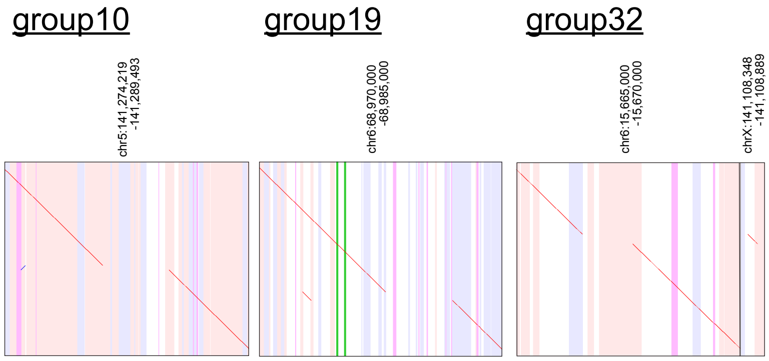

Inversions

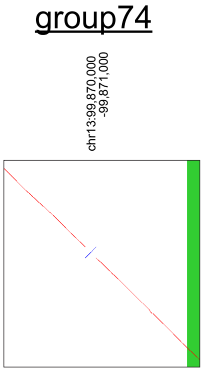

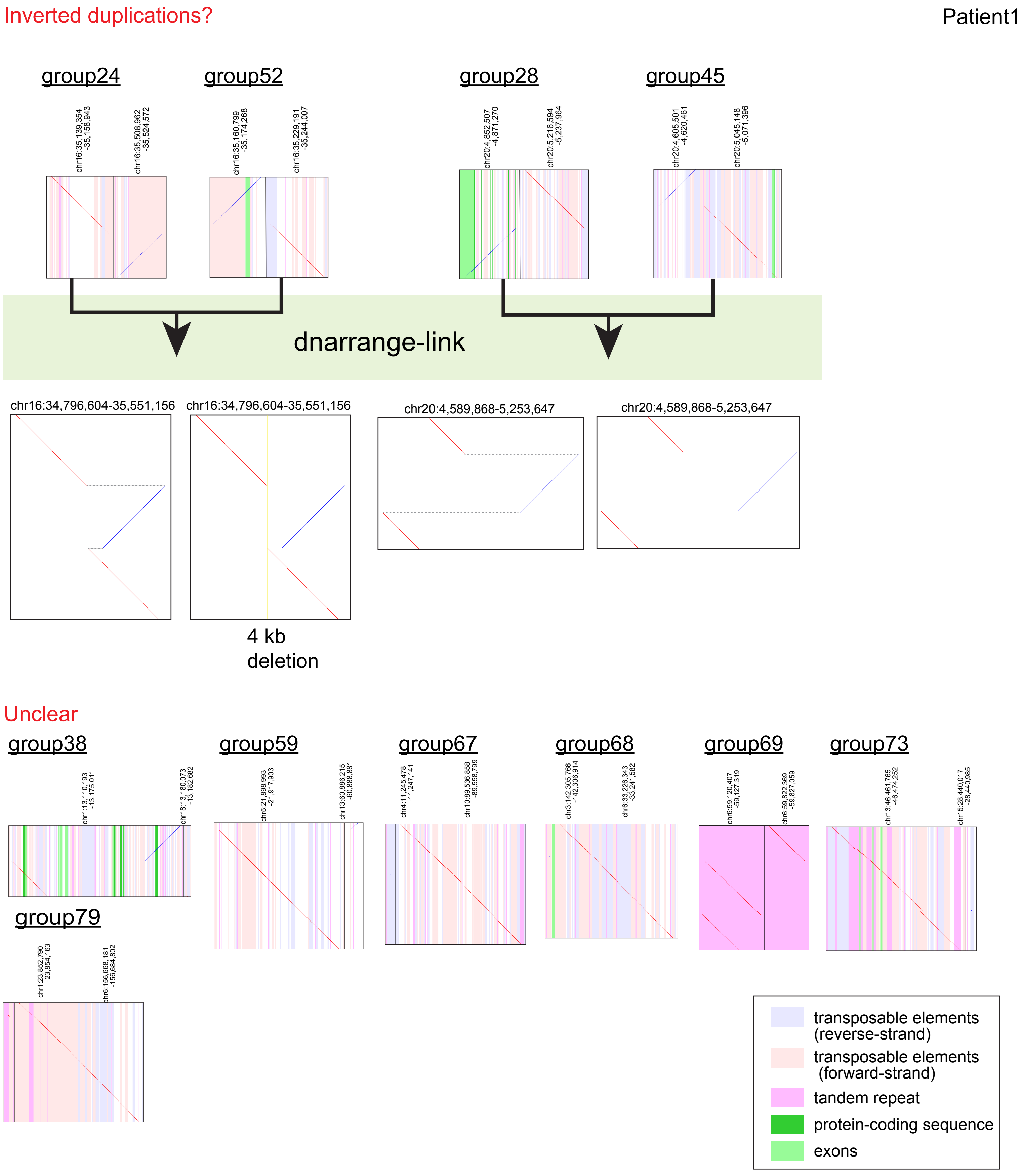

Fig S8. Other patient-only rearrangements in Patient 1.
Dot-plot pictures of lamassemble consensus sequences are shown. For some of the retrotranspositions (e.g. group57), the insertion is aligned to multiple chromosomes. In these cases, the insertion is aligned to different copies of the same type of retrotransposon (e.g. SVA). Our interpretation is that the true source copy is absent from (or misassembled in) the reference genome, so we get a fragmented alignment to different copies.

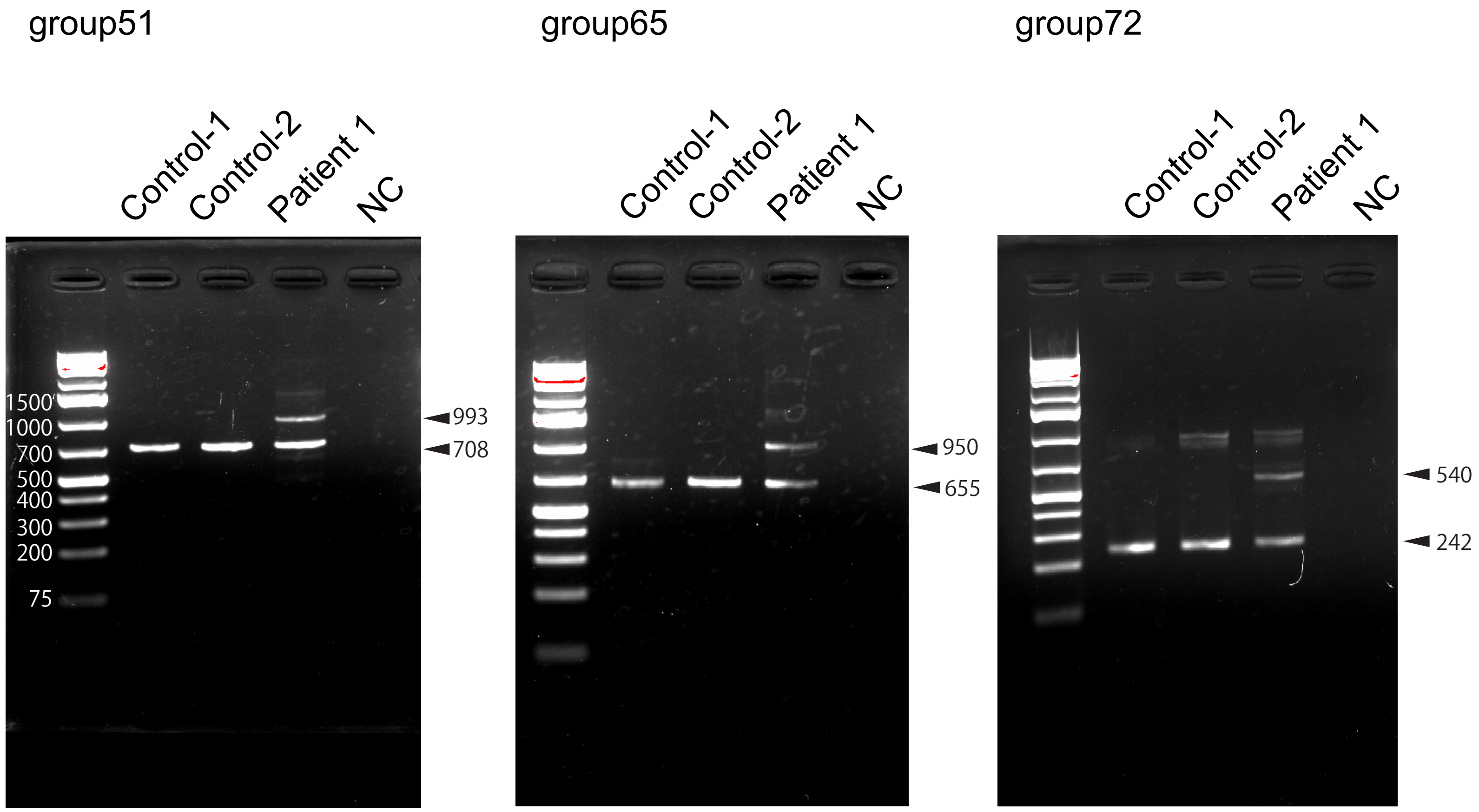

Fig S9. AluYa5 insertions in Patient 1

PCR confirmation for three TE-insertions in Patient1 (group51, 65 and 72). Primers were designed to amplify both normal and TE-inserted alleles. Agarose gel electrophoresis of the PCR products shows two PCR products in Patient 1 but not controls. The expected band size estimated from dnarrange are shown (arrow heads).

Control: control individuals without a disease. NC: non DNA template control.

.

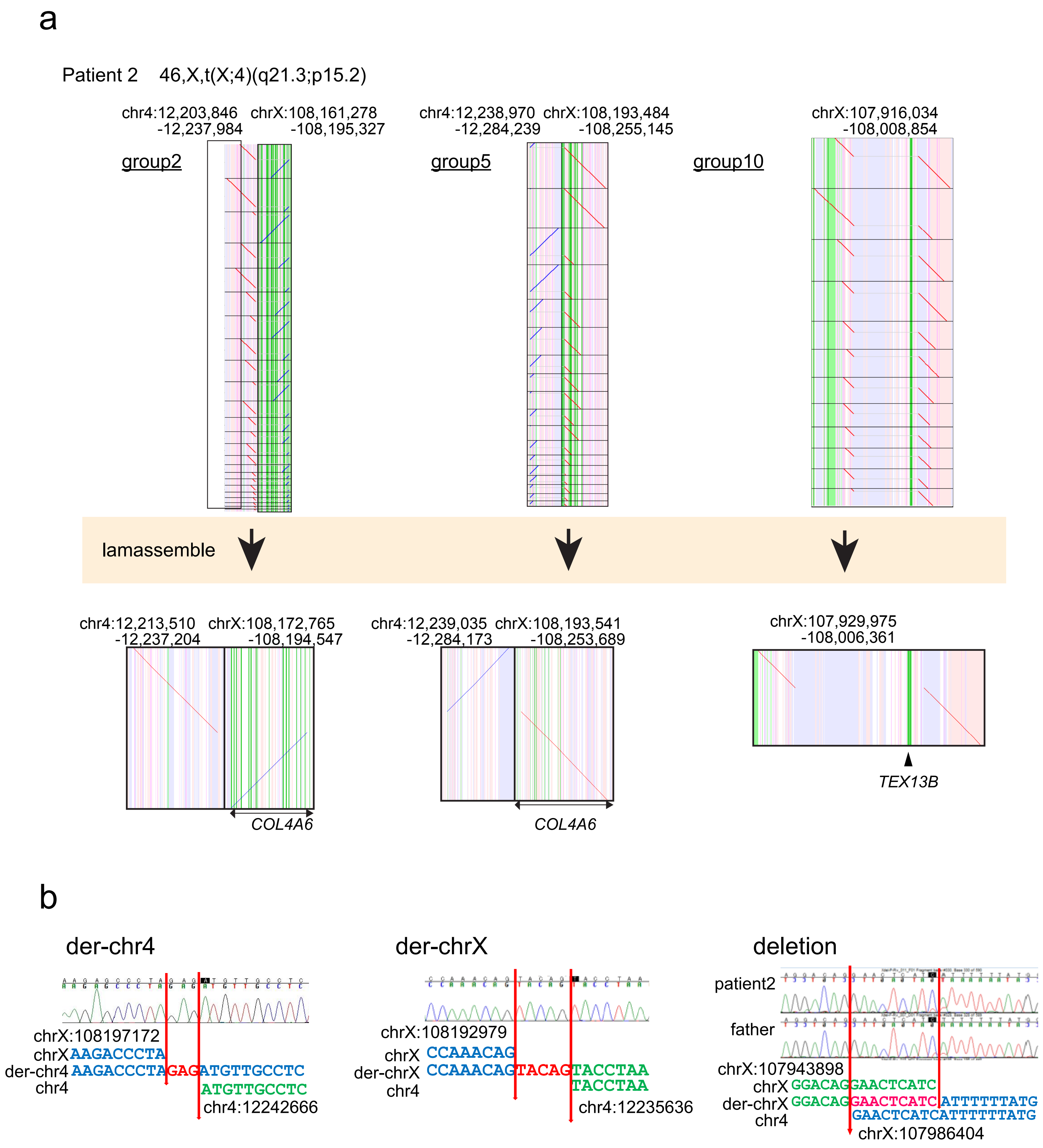
a
Patient 2 46,X,t(X;4)(q21.3;p15.2)
chr4:12,203,846 -12,237,984
chrX:108,161,278 -108,195,327
group2
chr4:12,238,970 -12,284,239
chrX:108,193,484 -108,255,145
group5
chrX:107,916,034 -108,008,854
group10
lamassemble
chr4:12,213,510 -12,237,204
chrX:108,172,765 -108,194,547
COL4A6
chr4:12,239,035 -12,284,173
chrX:108,193,541 -108,253,689
COL4A6
chrX:107,929,975 -108,006,361
TEX13B
b
der-chr4
chrX:108197172
chrX AAGACCCTA
der-chr4 AAGACCCTAGAGATGTTGCCTC
chr4 ATGTTGCCTC
chr4:12242666
der-chrX
chrX:108192979
chrX CCAAACAG
der-chrX CCAAACAGTACAGTACCTAA
chr4 TACCTAA
chr4:12235636
deletion
patient2
father
chrX:107943898
chrX GGACAGGAACTCATC
der-chrX GGACAGGAACTCATCATTTTTTATG
chr4 GAACTCATCATTTTTTATG
chrX:107986404

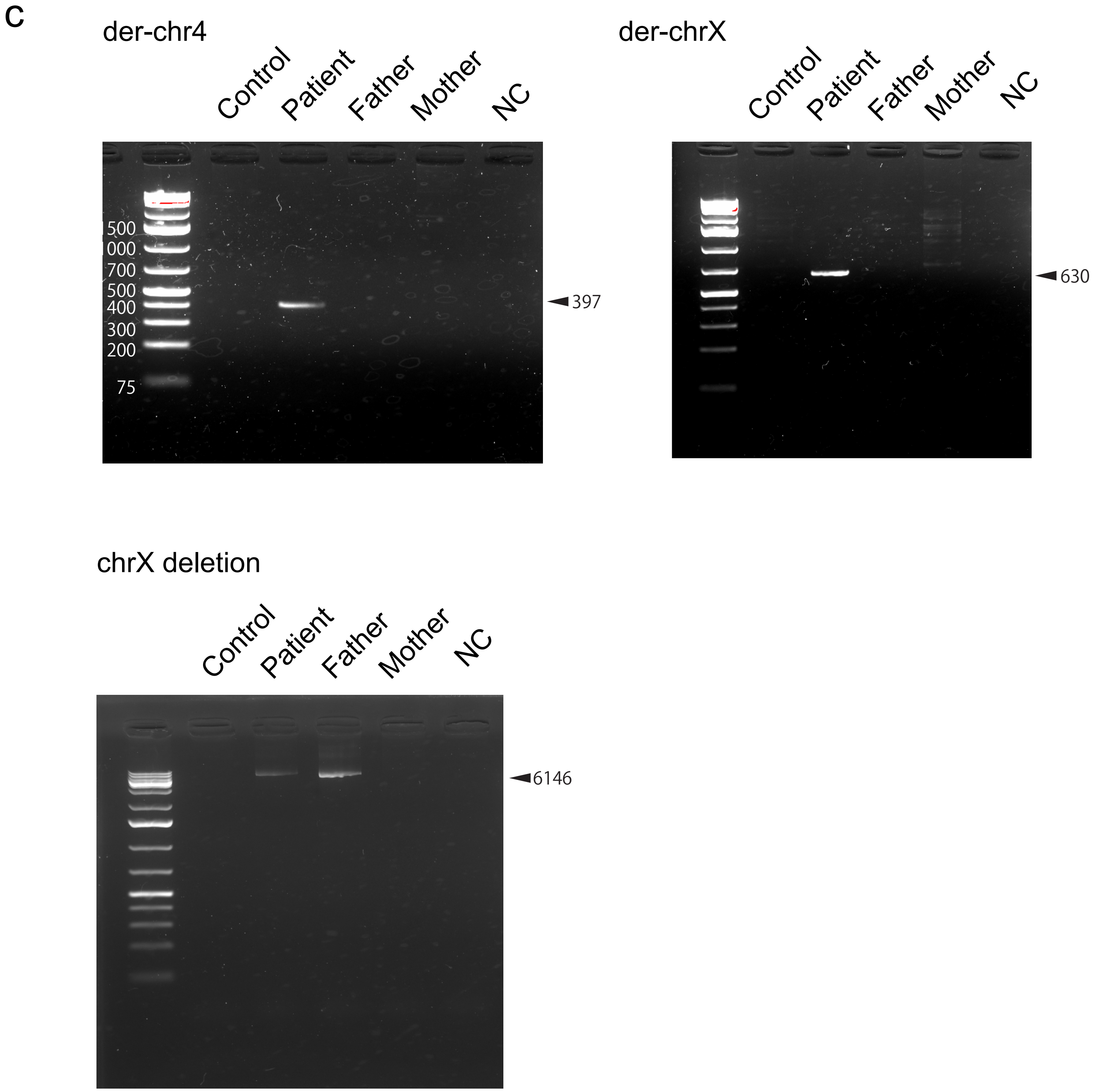

Fig S10. Reciprocal chromosomal translocation in Patient 2.
Dot-plot pictures of grouped rearranged reads and lamassemble consensus sequences at the translocation sites in Patient 2. Translocation t(X;4)(q21.3;p15.2) in Patient 2 disrupts the *COL4A6* gene. Near the translocation site, there is a 43-kb deletion, which eliminates the whole *TEX13B* gene. The vertical stripes indicate annotations in the reference genome: tandem repeats (purple), transposable elements (pink:forward-strand, blue:reverse-strand), green (exon) and dark green (protein-coding sequence). The horizontal black lines indicate boundaries between different dot-plots showing different reads. (b) Sanger sequence confirmation of the breakpoints. (c) Breakpoint PCR of the proband and parents shows the t(X;4) translocation is de novo but 43-kb deletion is inherited from father. Control: control individual without a disease. NC: non DNA template control.

Patient2

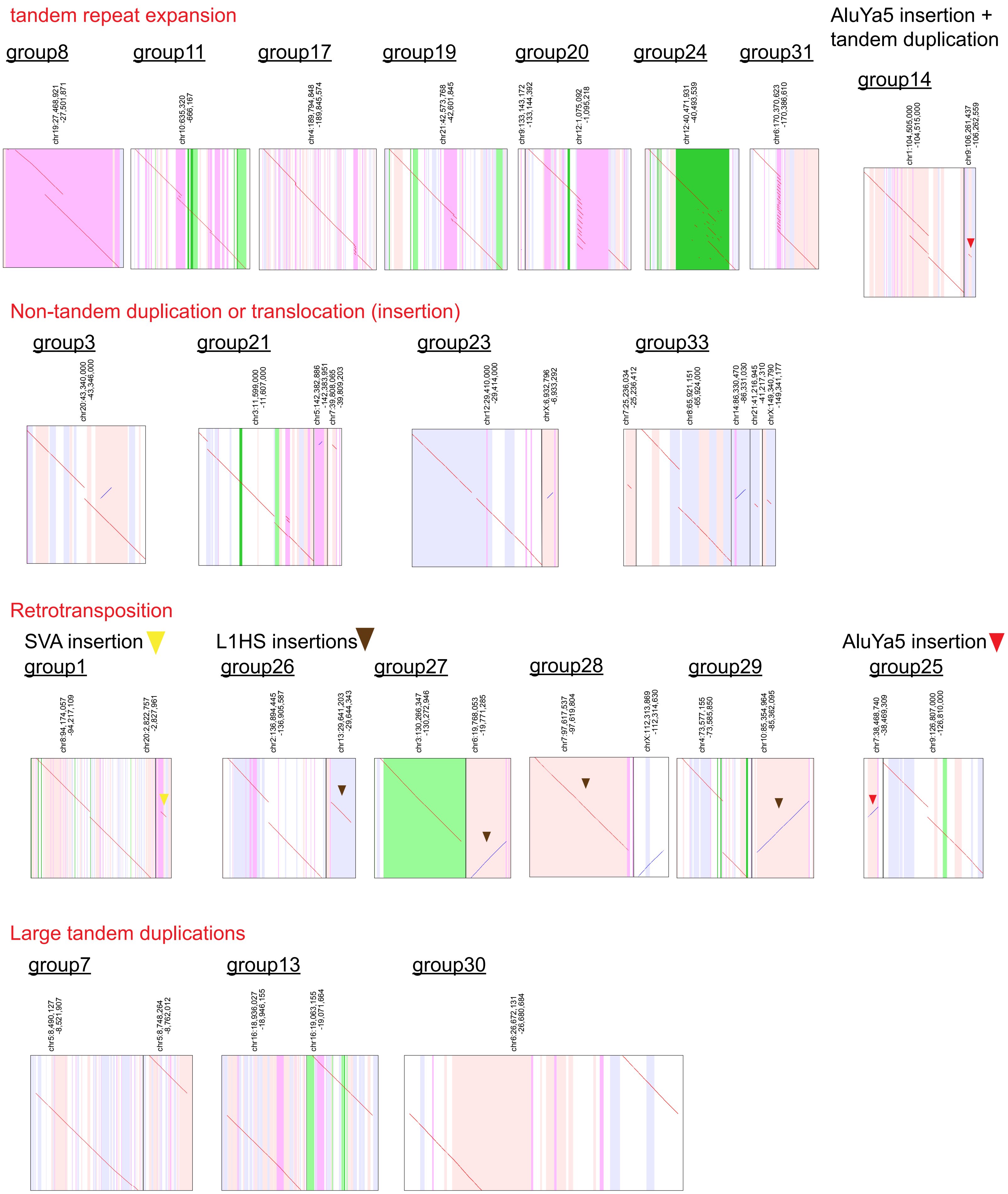

tandem repeat expansion
group8
chr19:27,468,921 -27,501,871
group11
chr10:635,320 -666,167
group17
chr4:189,794,848 -189,845,574
group19
chr21:42,573,768 -42,601,845
group20
chr9:133,143,172 -133,144,392
chr12:1,075,092 -1,095,218
group24
chr12:40,471,931 -40,493,539
group31
chr6:170,370,623 -170,386,610
AluYa5 insertion + tandem duplication
group14
chr1:104,505,000 -104,515,000
chr9:106,261,437 -106,262,559
Non-tandem duplication or translocation (insertion)
group3
chr20:43,340,000 -43,346,000
group21
chr3:11,599,000 -11,607,000
chr5:142,382,886 -142,383,951
chr7:39,808,065 -39,809,203
group23
chr12:29,410,000 -29,414,000
chrX:6,932,796 -6,933,292
group33
chr7:25,236,034 -25,236,412
chr8:65,921,151 -65,924,000
chr14:86,330,470 -86,331,030
chr21:41,216,945 -41,217,310
chrX:149,340,790 -149,341,177
Retrotransposition
SVA insertion
group1
chr8:94,174,057 -94,217,109
chr20:2,822,757 -2,827,961
L1HS insertions
group26
chr2:136,894,445 -136,905,587
chr13:29,641,203 -29,644,343
group27
chr3:130,266,347 -130,272,946
chr6:19,768,053 -19,771,285
group28
chr7:97,617,537 -97,619,804
chrX:112,313,869 -112,314,630
group29
chr4:73,577,155 -73,585,850
chr10:85,354,964 -85,362,095
AluYa5 insertion
group25
chr7:38,468,740 -38,469,309
chr9:126,807,000 -126,810,000
Large tandem duplications
group7
chr5:8,490,127 -8,521,907
chr5:8,748,264 -8,762,012
group13
chr16:18,936,027 -18,946,155
chr16:19,063,155 -19,071,664
group30
chr6:26,672,131 -26,680,684

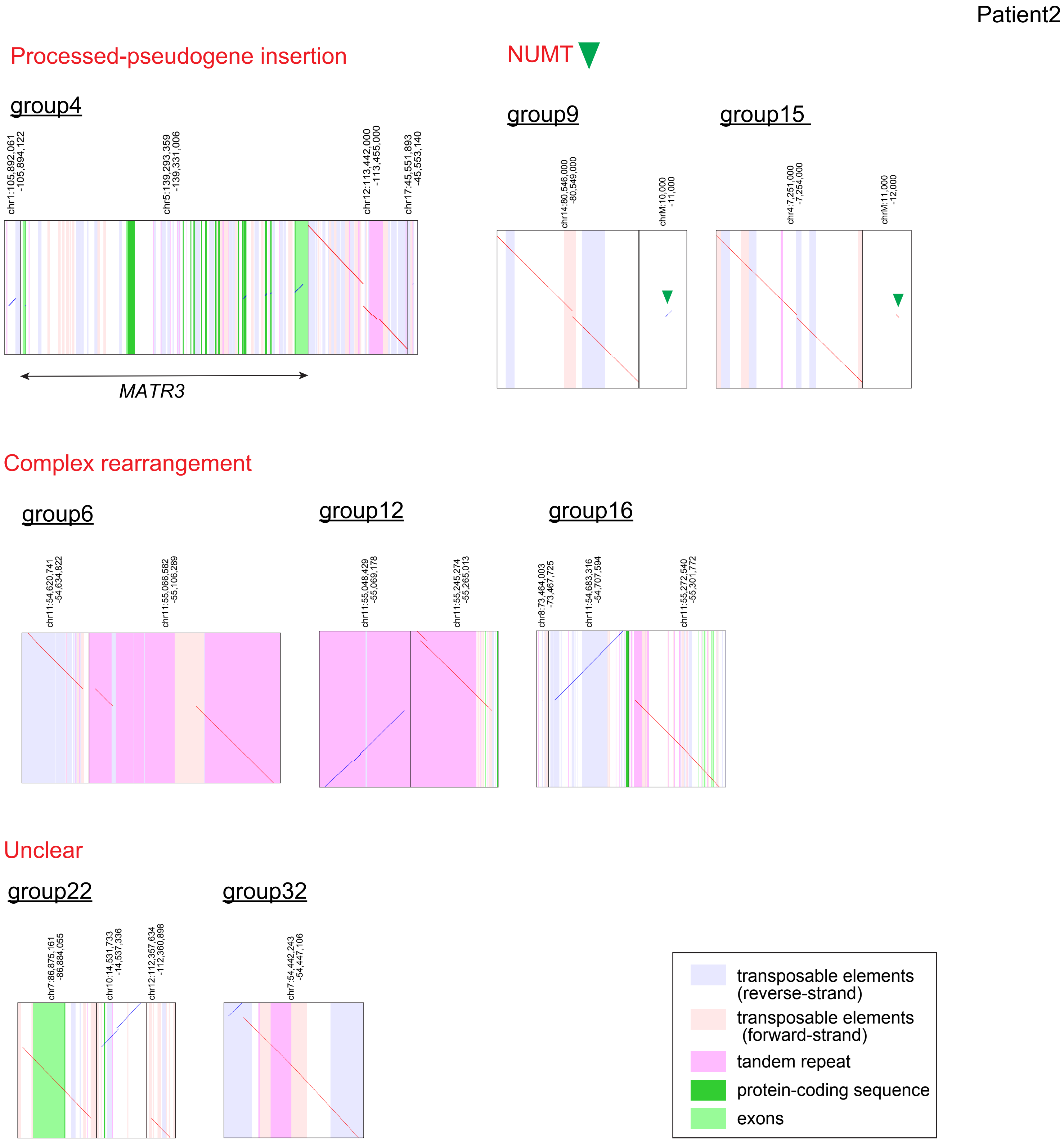

Fig S11. Other patient-only rearrangements in Patient 2.
Other patient-only rearrangements in Patient 2. Dot-plot pictures of lamassemble consensus sequence are shown. Patient 2 also has a processed-pseudogene insertion into chr12, which aligns to exons of *MATR3* in chr5. Part of this insertion is aligned, perhaps incorrectly, to a *MATR3* processed pseudogene in chr1.

a

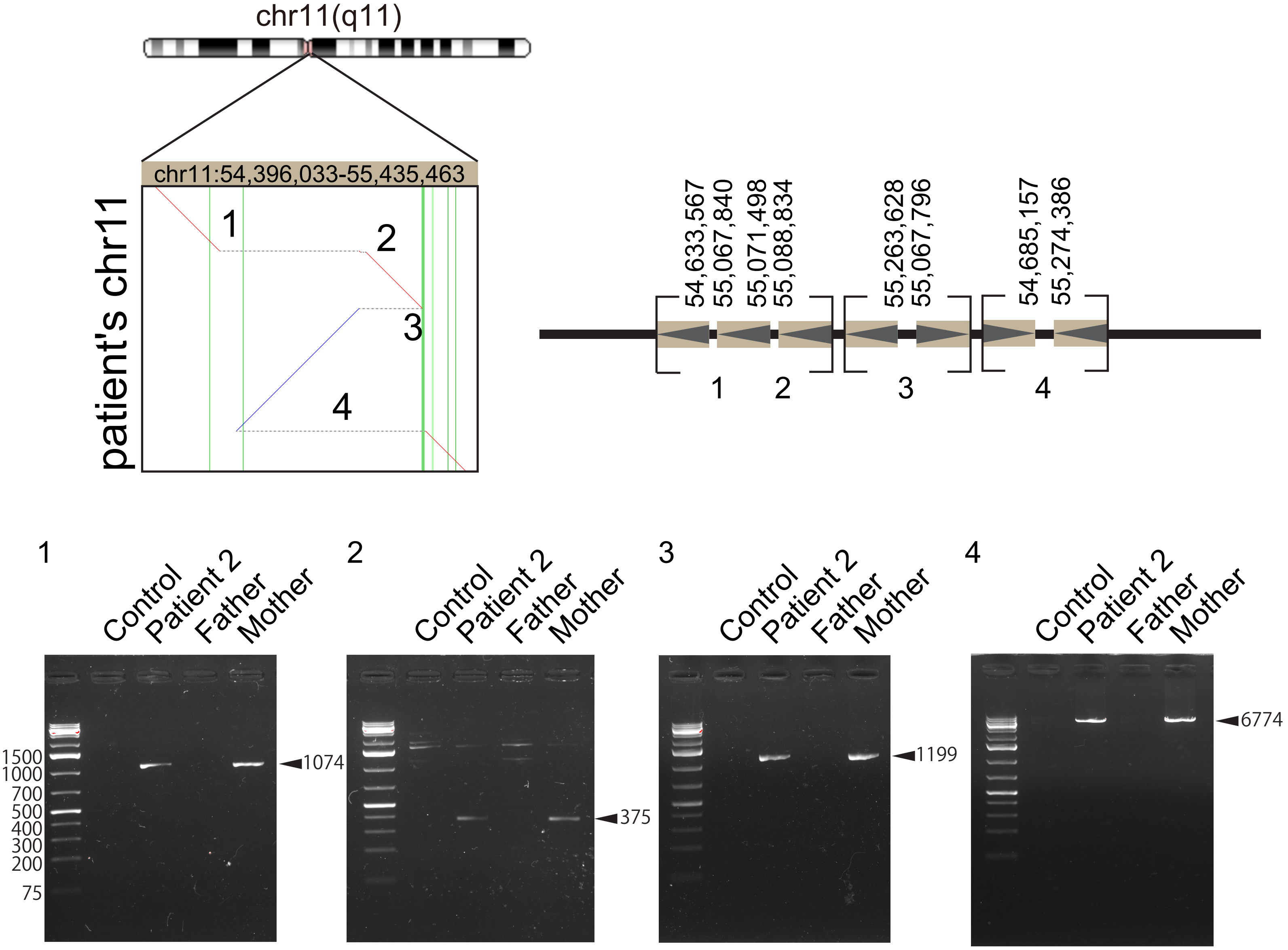
chr11(q11)
chr11:54,396,033-55,435,463
patient's chr11
1
2
3
4
54,633,567
55,067,840
55,071,498
55,088,834
55,263,628
55,067,796
54,685,157
55,274,386
1 2
3
4
1
Control
Patient 2
Father
Mother
1500
1000
700
500
400
300
200
75
1074
2
Control
Patient 2
Father
Mother
375
3
Control
Patient 2
Father
Mother
1199
4
Control
Patient 2
Father
Mother
6774

b

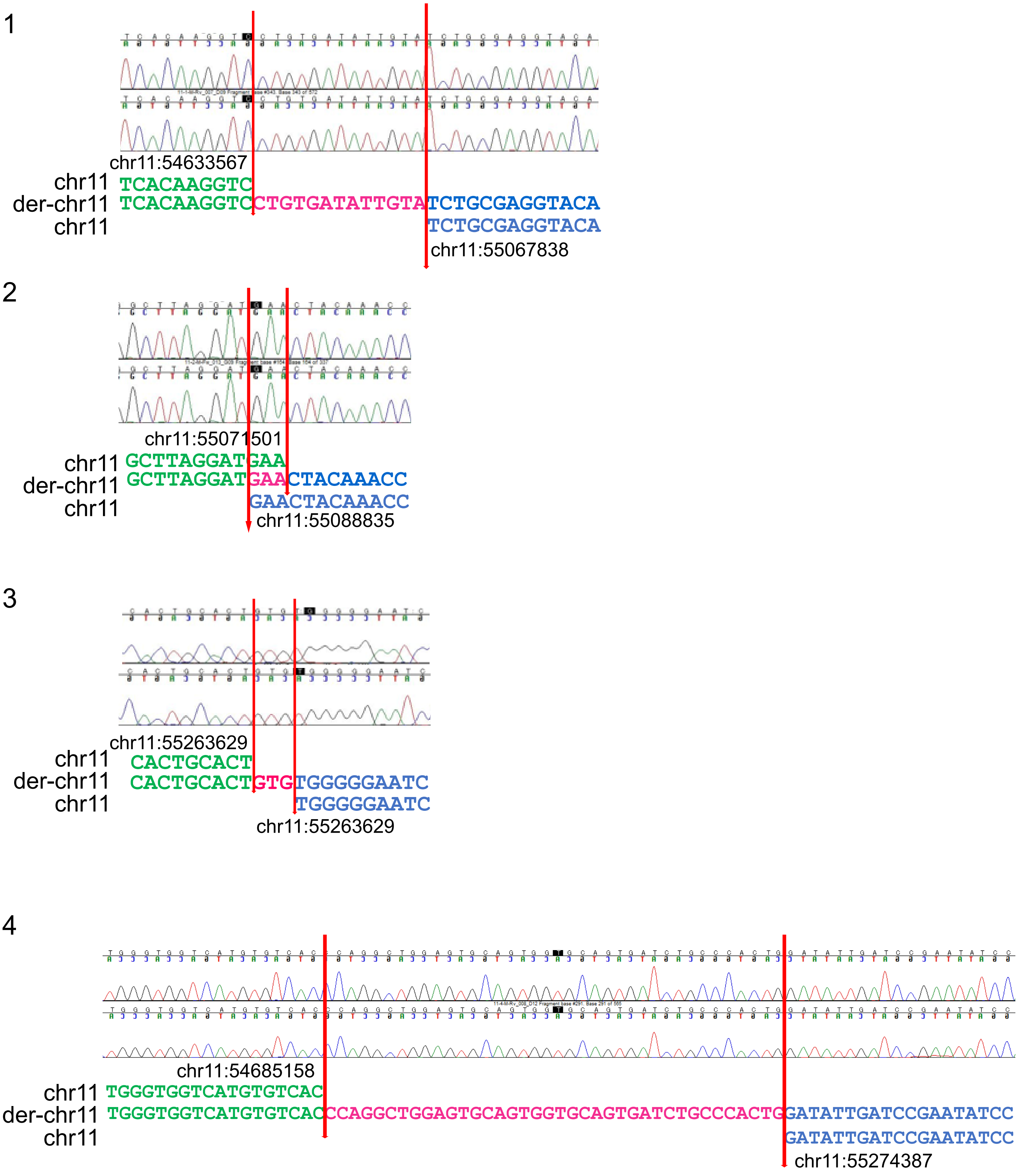

Fig S12. complex chr11 rearrangement in Patient 2
a. PCR confirmation for complex chr11 rearrangement in Patient2. Four breakpoints are confirmed by PCR. This rearrangement was inherited from mother. Arrow heads: predicted PCR product size (bp). b. Sanger sequence confirmation of the breakpoints.
.

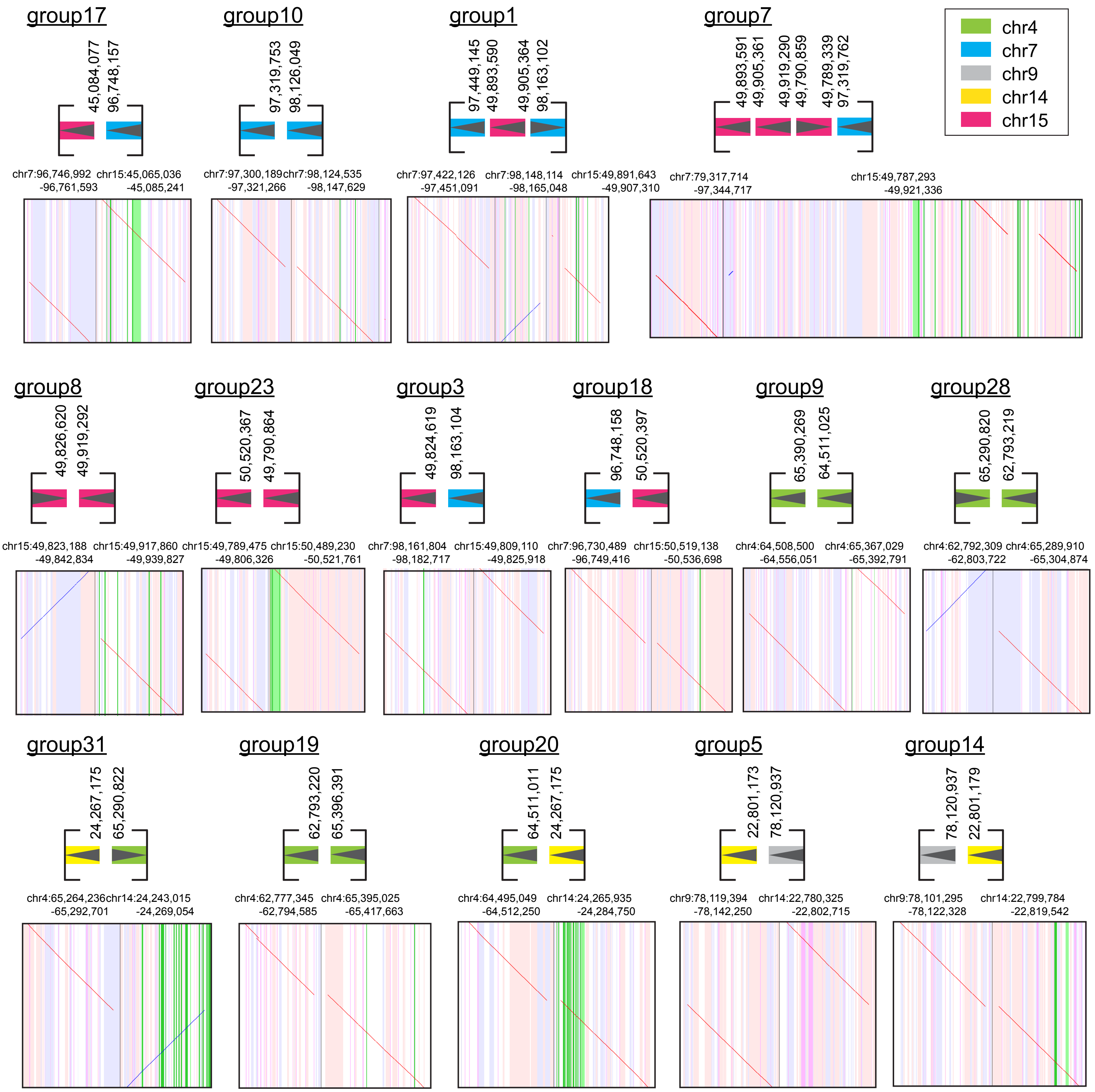

Fig S13. Rearrangement groups detected in two reciprocal chromosomal translocations in Patient 3.
Dot-plot pictures of lamassemble consensus sequences aligned to the human reference genome that explain translocation t(7;15)(q21;q15) and t(9,14)(q21;q11.2) in Patient 3. The vertical stripes indicate annotations in the reference genome: tandem repeats (purple), transposable elements (pink:forward-strand, blue:reverse-strand), green (exon) and dark green (protein-coding sequence).

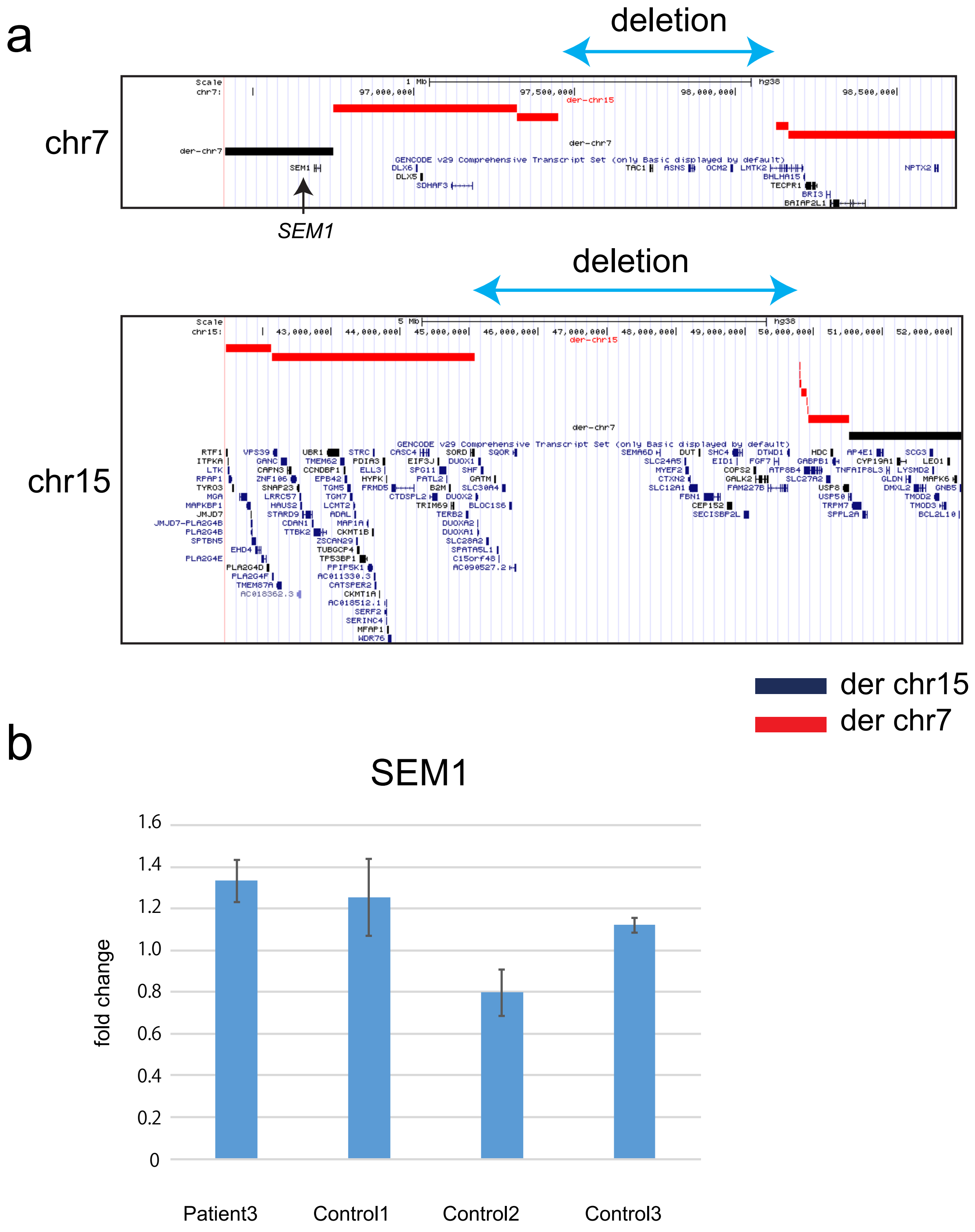

Fig S14. Genes at the rearrangement loci in Patient 3.
a. Joined rearranged fragments from Patient 3 are aligned to the reference genome and shown by UCSC genome browser. Several genes are completely deleted or disrupted by breakpoints. b. RT-PCR of SEM1. Error bars represent standard deviation.

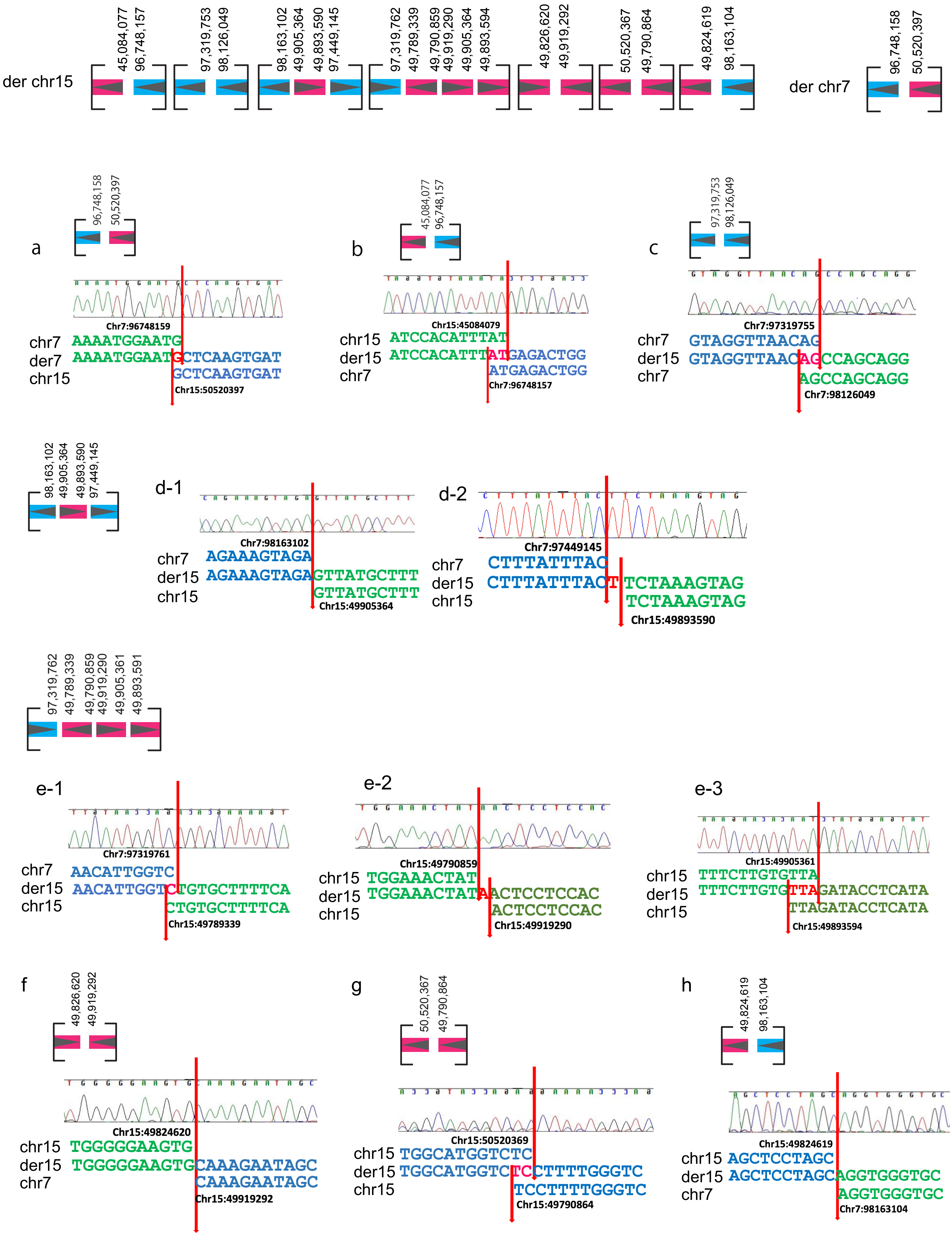

der chr15
45,084,077
96,748,157
97,319,753
98,126,049
98,163,102
49,905,364
49,893,590
97,449,145
97,319,762
49,789,339
49,790,859
49,919,290
49,905,364
49,893,594
49,826,620
49,919,292
50,520,367
49,790,864
49,824,619
98,163,104
der chr7
96,748,158
50,520,397
a
Chr7:96748159
chr7 AAAATGGAATG
der7 AAAATGGAATGCTCAAGTGAT
chr15 GCTCAAGTGAT
Chr15:50520397
b
Chr15:45084079
chr15 ATCCACATTTAT
der15 ATCCACATTTATGAGACTGG
chr7 ATGAGACTGG
Chr7:96748157
c
97,319,753
98,126,049
Chr7:97319755
chr7 GTAGGTTAACAG
der15 GTAGGTTAACAGCCAGCAGG
chr7 AGCCAGCAGG
Chr7:98126049
d-1
Chr7:98163102
chr7 AGAAAGTAGA
der15 AGAAAGTAGAGTTATGCTTT
chr15 GTTATGCTTT
Chr15:49905364
d-2
Chr7:97449145
chr7 CTTTATTTAC
der15 CTTTATTTACTTCTAAAGTAG
chr15 TCTAAAGTAG
Chr15:49893590
49,905,361
49,893,591
e-1
Chr7:97319761
chr7 AACATTGGTC
der15 AACATTGGTCTGTGCTTTTCA
chr15 CTGTGCTTTTCA
Chr15:49789339
e-2
Chr15:49790859
chr15 TGGAAACTAT
der15 TGGAAACTATAACTCCTCCAC
chr15 ACTCCTCCAC
Chr15:49919290
e-3
Chr15:49905361
chr15 TTTCTTGTGTTA
der15 TTTCTTGTGTTAGATACCTCATA
chr15 TTAGATACCTCATA
Chr15:49893594
f
Chr15:49824620
chr15 TGGGGGAAGTG
der15 TGGGGGAAGTGCAAAGAATAGC
chr7 CAAAGAATAGC
Chr15:49919292
g
Chr15:50520369
chr15 TGGCATGGTCTC
der15 TGGCATGGTCTCCTTTTGGGTC
chr15 TCCTTTTGGGTC
Chr15:49790864
h
Chr15:49824619
chr15 AGCTCCTAGC
der15 AGCTCCTAGCAGGTGGGTGC
chr7 AGGTGGGTGC
Chr7:98163104

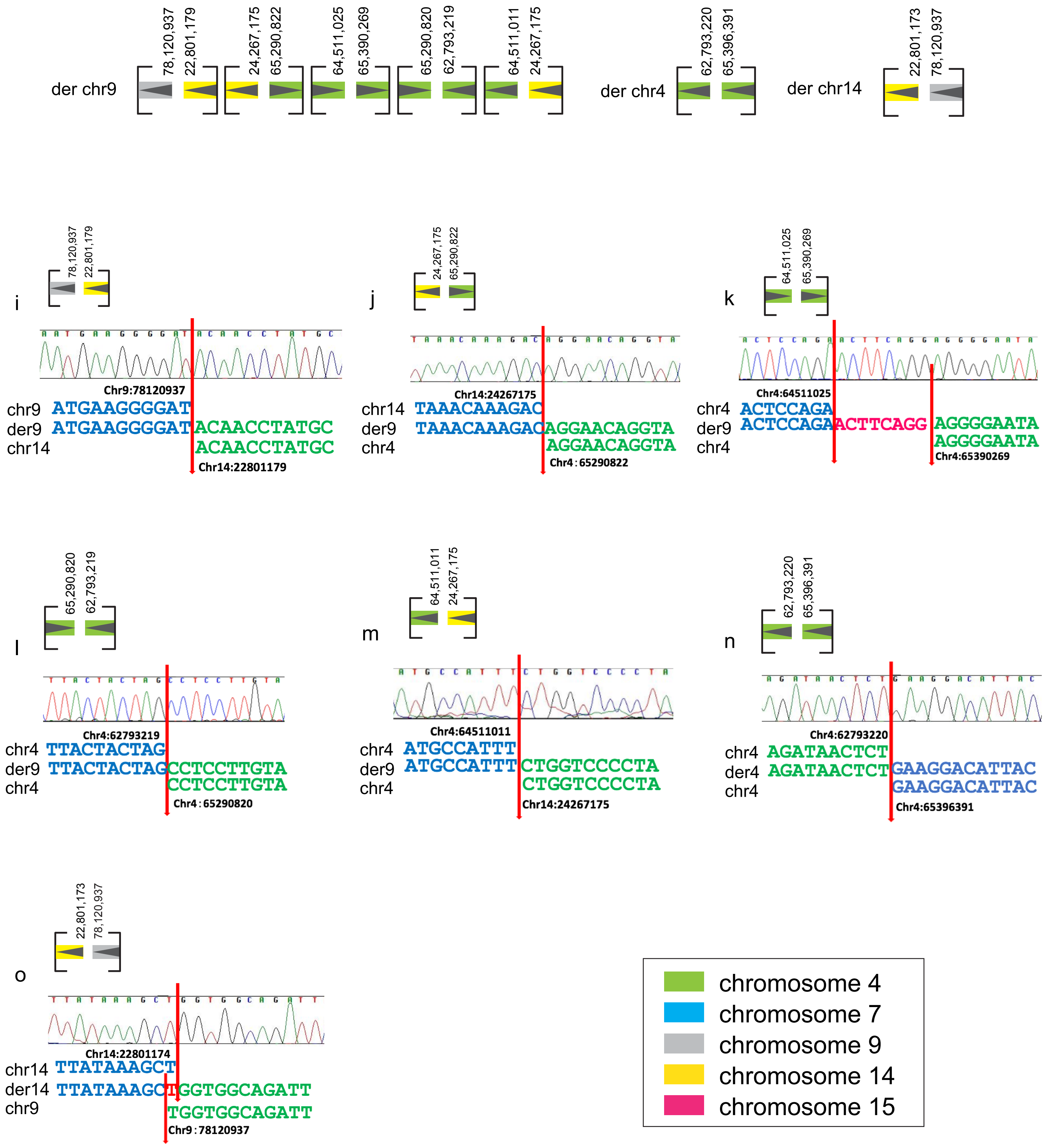

Fig S15. Sanger-sequence confirmation of the breakpoints.
Sanger sequence confirmation of all 18 breakpoints in Patient 3. Electropherograms of the Sanger sequencing data of each breakpoint are shown with the schematic picture of the rearrangements.

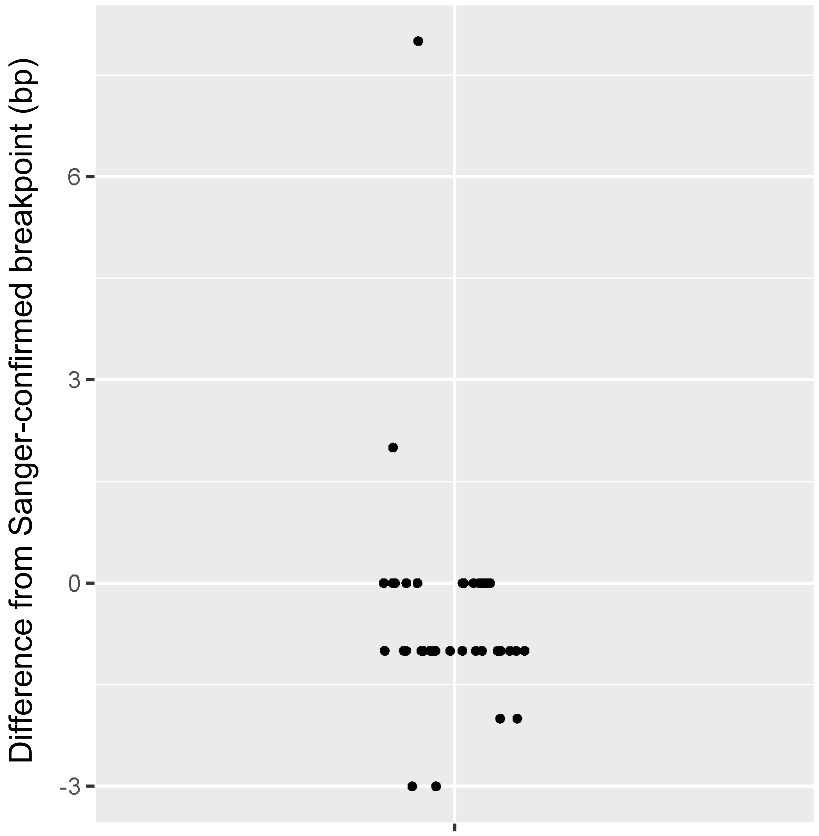

Fig S16. Accuracy of breakpoint prediction by dnarrange confirmed by Sanger sequencing.
Difference between Sanger sequence-confirmed breakpoints and dnarrange predictions. There is usually 0 or 1 bp difference from the Sanger-sequence results.

Patient 3

Tandem multiplication

group12 group15 group16 group21 group27 group30 group32

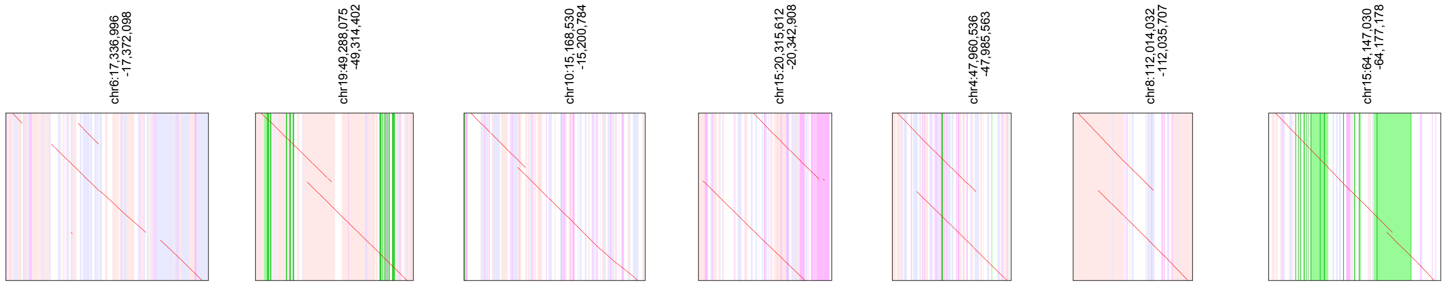

Tandem repeat expansion

group25 group29 group33

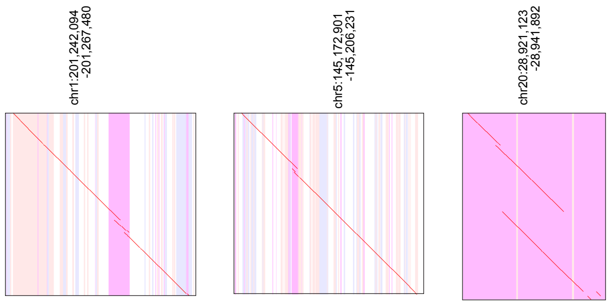

Non-tandem duplication

group13 group42

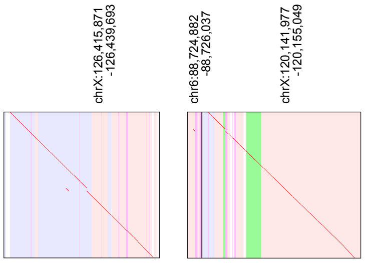

Large tandem duplications

group4

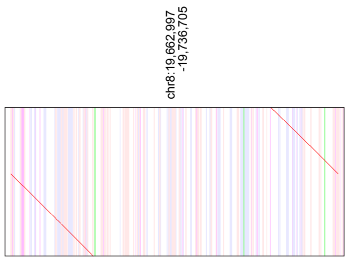

Inversion

group34

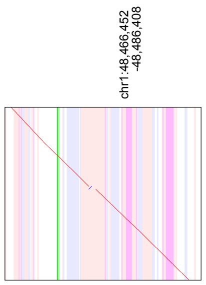

Deletions (or translocations)

group2 group11 group24 group26 group41

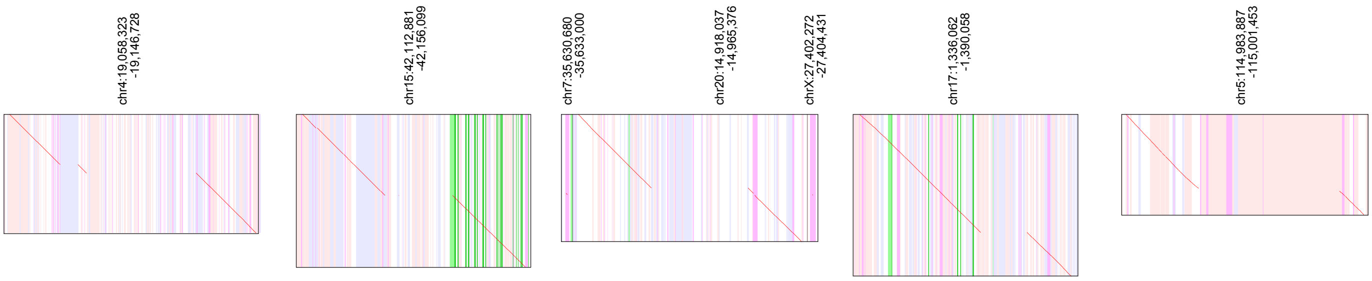

Patient 3

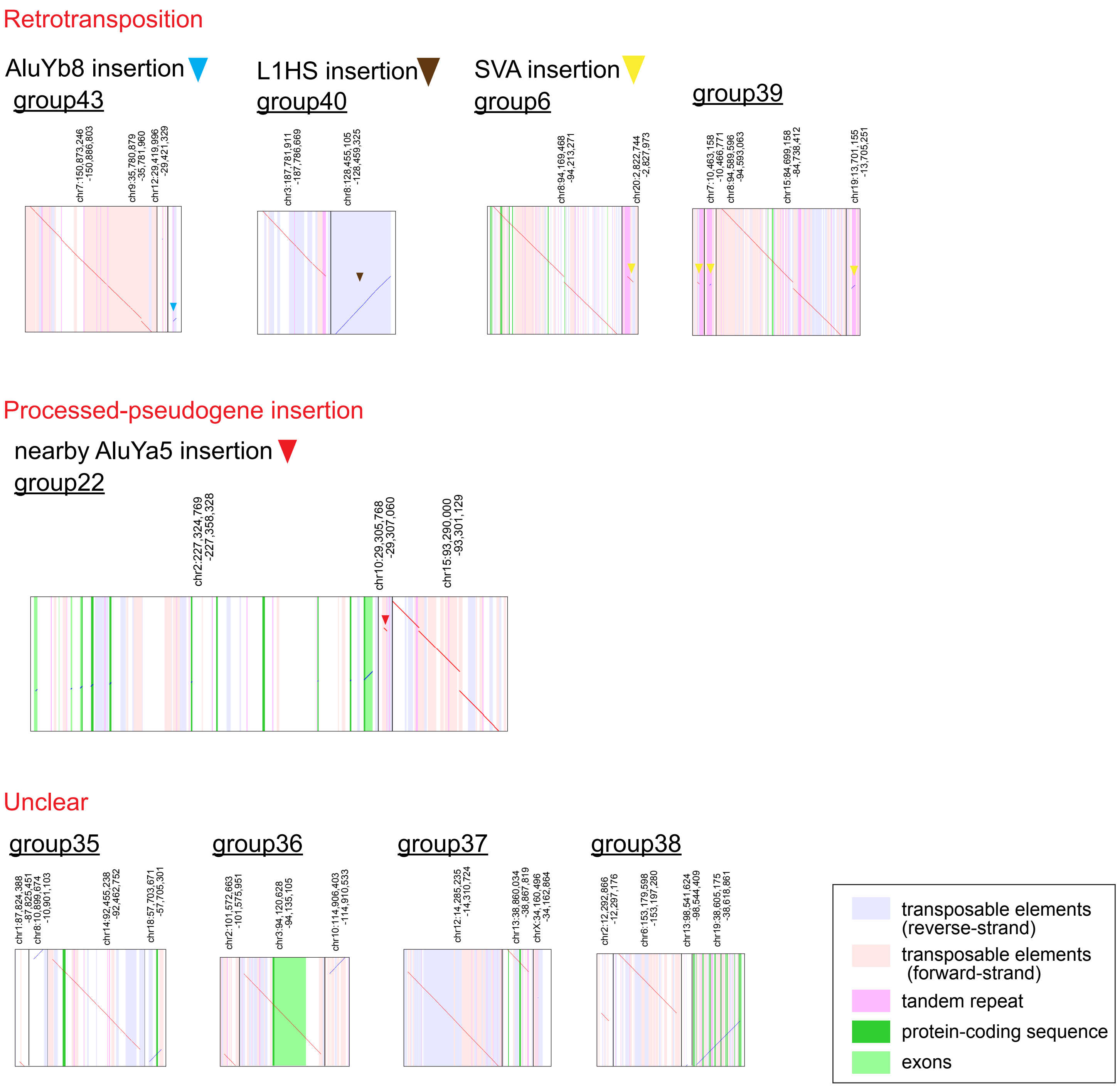

Fig S17. Other patient-only rearrangements in Patient 3.
Other patient-only rearrangements in Patient 3. Dot-plot pictures of lamassemble consensus sequences are shown. The vertical stripes indicate annotations in the reference genome: tandem repeats (purple), transposable elements (pink:forward-strand, blue:reverse-strand), green (exon) and dark green (protein-coding sequence).

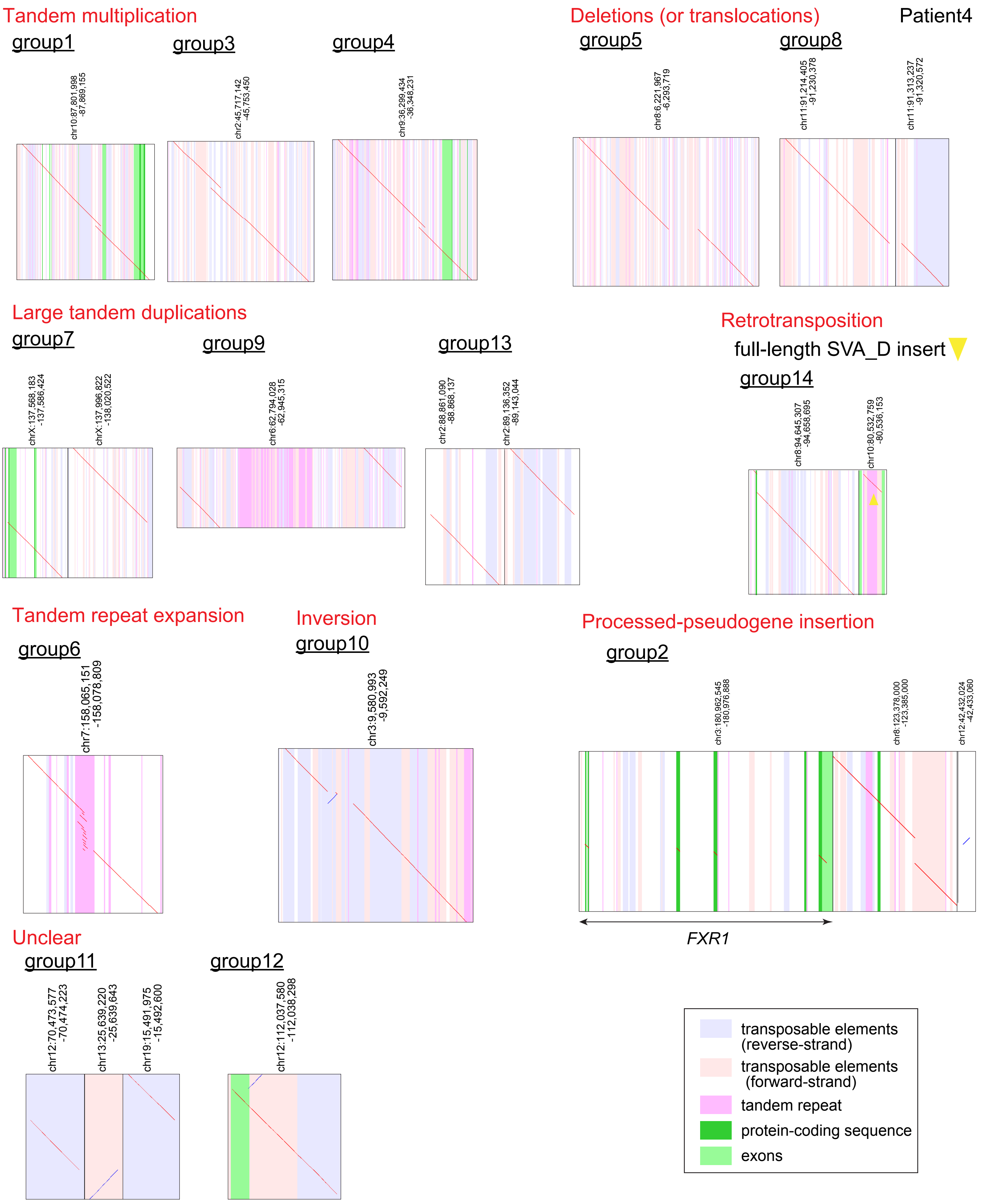

Fig S18. Patient-only rearrangements in Patient 4.
Other patient-only rearrangements in Patient 4. Patient 4 has a processed pseudogene insertion from exons of *FXR1*. Part of this insertion is aligned, perhaps incorrectly, to an *FXR1* processed pseudogene in chr12.

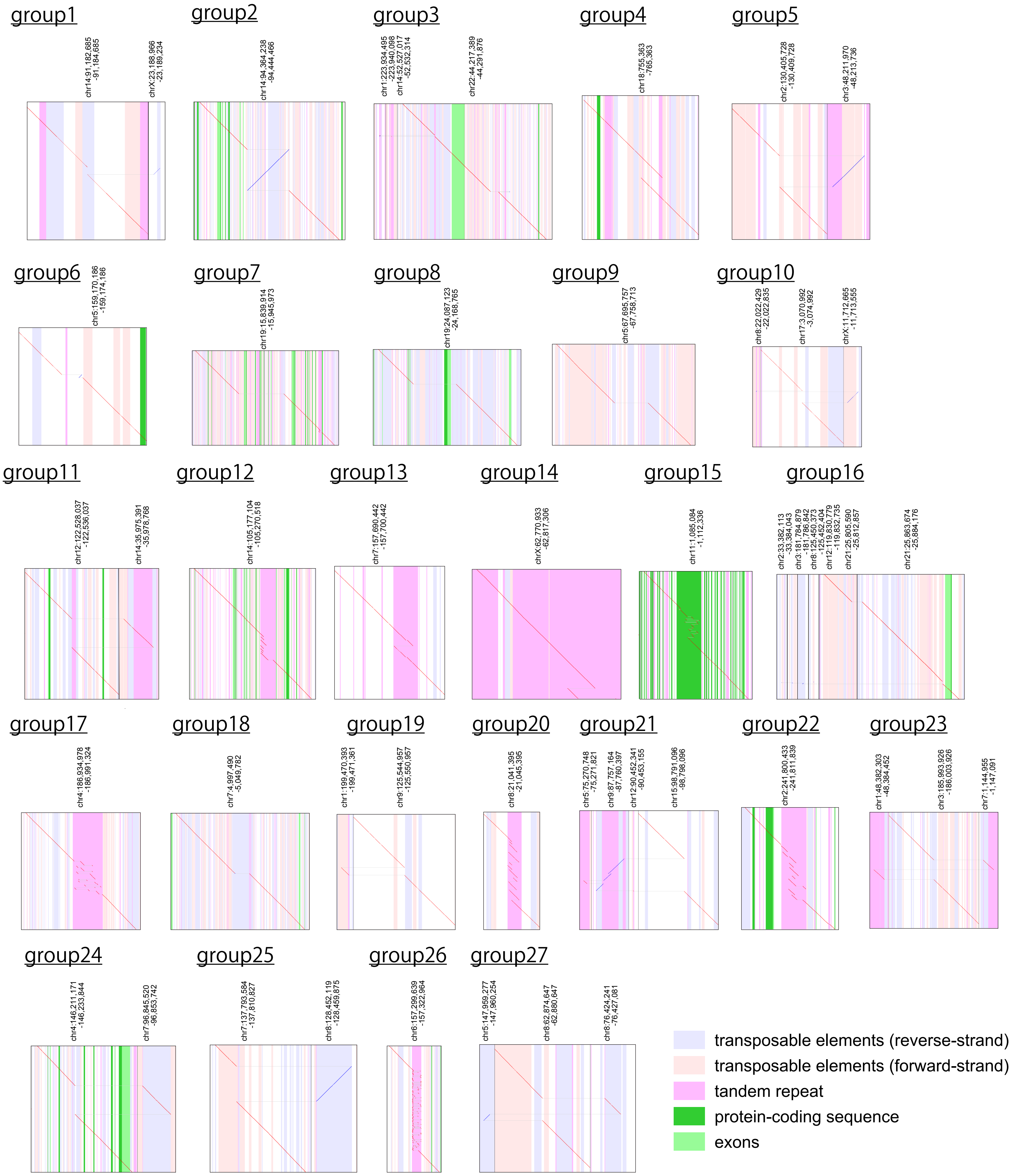

Fig S19. Control-1 only rearrangements.
We could identify 27 Control-1-only rearragements by using other unrelated 30 controls (Control-4 to Control-33). dnarrange automatically removed 14 of them using mother (Control-3), and 12 using father (Control-2) as controls. Group-23 was not filtered but this TE-insertion is present in Control-3.

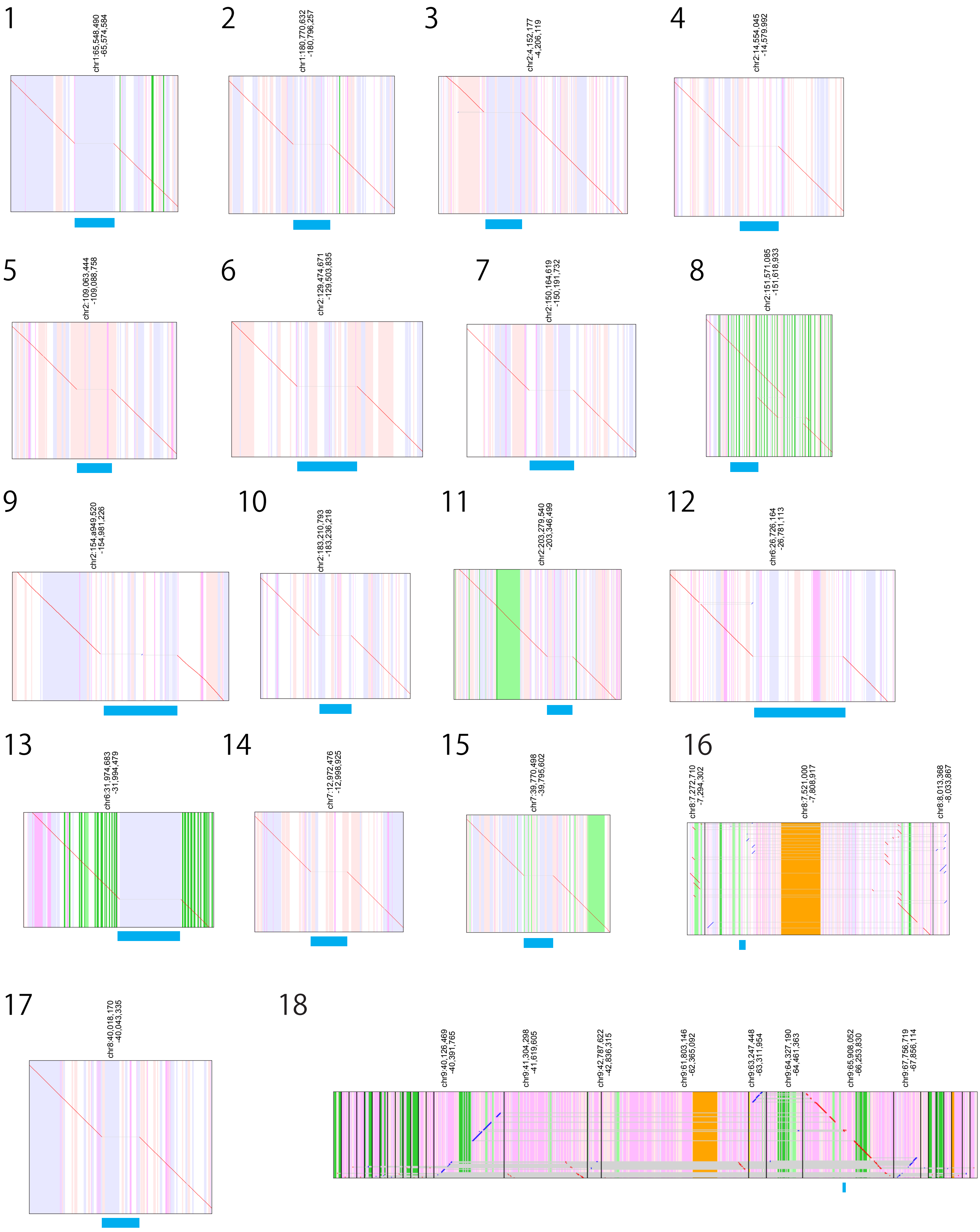

1
chr1:65,548,490
-65,574,584
2
chr1:180,770,632
-180,796,257
3
chr2:4,152,177
-4,206,119
4
chr2:14,554,045
-14,579,992
5
chr2:109,063,444
-109,088,758
6
chr2:129,474,671
-129,503,835
7
chr2:150,164,619
-150,191,732
8
chr2:151,571,085
-151,618,933
9
chr2:154,a949,520
-154,981,226
10
chr2:183,210,793
-183,236,218
11
chr2:203,279,540
-203,346,499
12
chr6:26,726,164
-26,781,113
13
chr6:31,974,683
-31,994,479
14
chr7:12,972,476
-12,998,925
15
chr7:39,770,498
-39,795,602
16
chr8:7,272,710
-7,294,302
chr8:7,521,000
-7,808,917
chr8:8,013,368
-8,033,867
17
chr8:40,018,170
-40,043,335
18
chr9:40,126,469
-40,391,765
chr9:41,304,298
-41,619,605
chr9:42,787,622
-42,836,315
chr9:61,803,146
-62,365,092
chr9:63,247,448
-63,311,954
chr9:64,327,190
-64,461,363
chr9:65,908,052
-66,253,830
chr9:67,756,719
-67,856,114

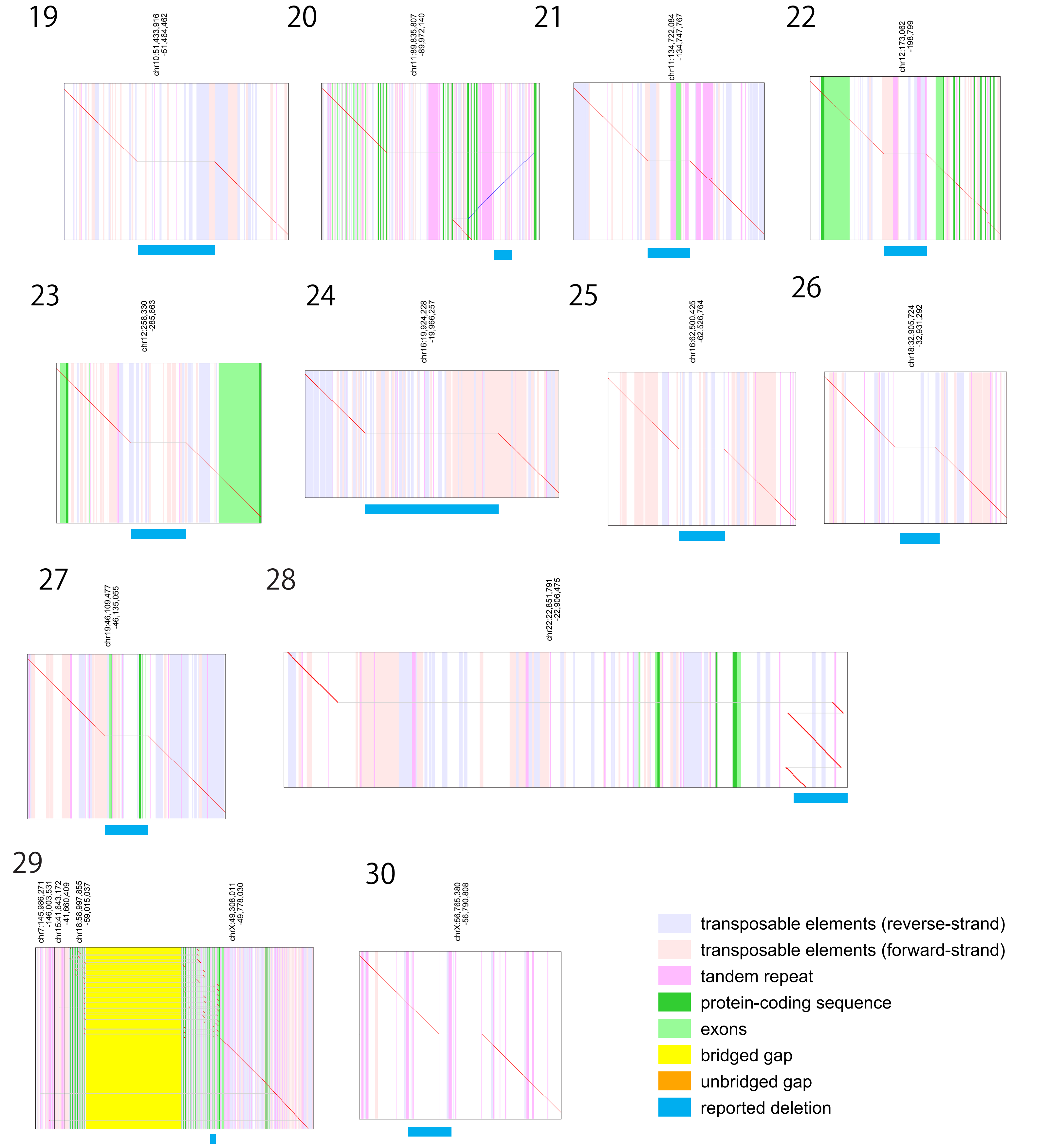

Fig S20. Published deletions in NA12878 were all detected by dnarrnage with further complexity.
dnarrange from NA12878 nanopore sequencing (rel6, https://github.com/nanopore-wgs-consortium) identifies 30 published deletions. Eight of them have further complexity than simple deletions (3, 8, 9, 16, 18, 20, 28, 29).
For 8, 16, 18, 29, we used latest lamassemble version (1.3.0) because it can handle repeats better. In site 30, we find the deletion at a location shifted from what was reported. This region has tandem repeats, as indicated by the repeating pattern of vertical stripes. The deletion is of one repeat unit, so its location has some ambiguity. In site 28, the reported deletion is also of one tandem repeat unit.

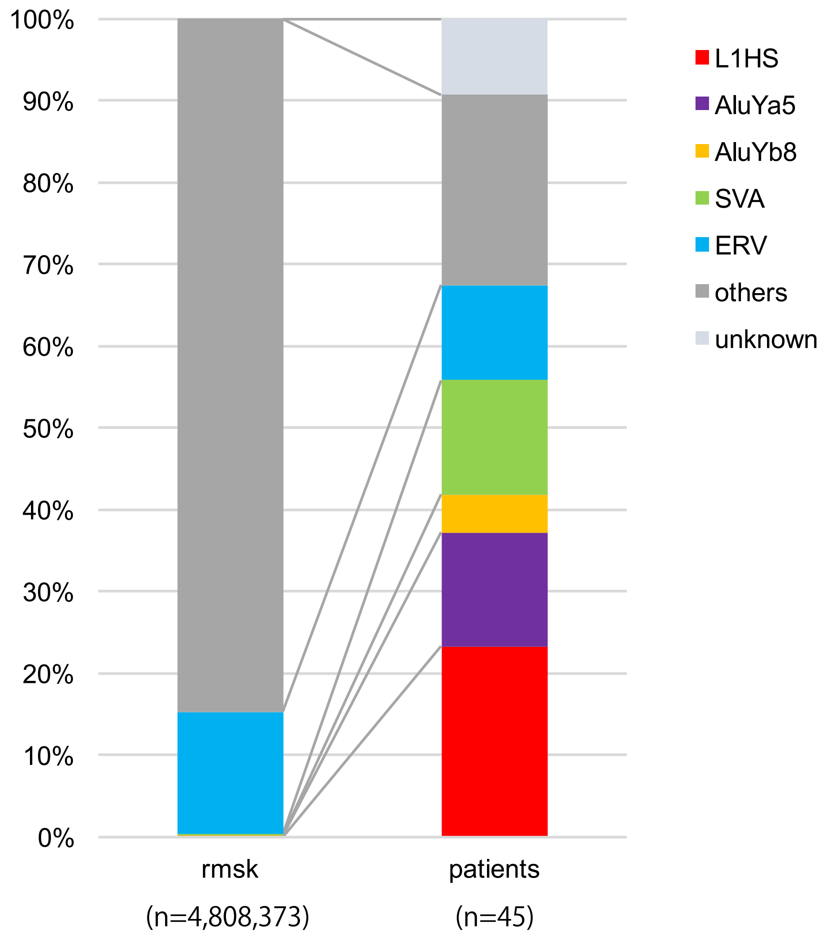

Fig S21. Active TEs are enriched in insertions.
Proportions of various transposable element types in the reference genome (rmsk, https://genome.ucsc.edu) and in patient-specific insertions (patients).

References

1. Bano G, Mansour S, Nussey S: **The association of primary hyperparathyroidism and primary ovarian failure: a de novo t(X; 2) (q22p13) reciprocal translocation.** *Eur J Endocrinol* 2008, **158:**261-263.
2. Nishimura-Tadaki A, Wada T, Bano G, Gough K, Warner J, Kosho T, Ando N, Hamanoue H, Sakakibara H, Nishimura G, et al: **Breakpoint determination of X;autosome balanced translocations in four patients with premature ovarian failure.** *J Hum Genet* 2011, **56:**156-160.
3. Saitsu H, Kurosawa K, Kawara H, Eguchi M, Mizuguchi T, Harada N, Kaname T, Kano H, Miyake N, Toda T, Matsumoto N: **Characterization of the complex 7q21.3 rearrangement in a patient with bilateral split-foot malformation and hearing loss.** *Am J Med Genet A* 2009, **149A:**1224-1230.
4. Saitsu H, Osaka H, Sugiyama S, Kurosawa K, Mizuguchi T, Nishiyama K, Nishimura A, Tsurusaki Y, Doi H, Miyake N, et al: **Early infantile epileptic encephalopathy associated with the disrupted gene encoding Slit-Robo Rho GTPase activating protein 2 (SRGAP2).** *Am J Med Genet A* 2012, **158A:**199-205.
5. Morgulis A, Gertz EM, Schaffer AA, Agarwala R: **WindowMasker: window-based masker for sequenced genomes.** *Bioinformatics* 2006, **22:**134-141.
6. Sone J, Mitsuhashi S, Fujita A, Mizuguchi T, Hamanaka K, Mori K, Koike H, Hashiguchi A, Takashima H, Sugiyama H, et al: **Long-read sequencing identifies GGC repeat expansions in NOTCH2NLC associated with neuronal intranuclear inclusion disease.** *Nat Genet* 2019, **51:**1215-1221.
7. Hamada M, Ono Y, Asai K, Frith MC: **Training alignment parameters for arbitrary sequencers with LAST-TRAIN.** *Bioinformatics* 2017, **33:**926-928.
8. Richardson SR, Doucet AJ, Kopera HC, Moldovan JB, Garcia-Perez JL, Moran JV: **The Influence of LINE-1 and SINE Retrotransposons on Mammalian Genomes.** *Microbiol Spectr* 2015, **3:**MDNA3-0061-2014.
9. Scherer SW, Poorkaj P, Allen T, Kim J, Geshuri D, Nunes M, Soder S, Stephens K, Pagon RA, Patton MA, et al.: **Fine mapping of the autosomal dominant split hand/split foot locus on chromosome 7, band q21.3-q22.1.** *Am J Hum Genet* 1994, **55:**12-20.
10. Crackower MA, Scherer SW, Rommens JM, Hui CC, Poorkaj P, Soder S, Cobben JM,

Hudgins L, Evans JP, Tsui LC: **Characterization of the split hand/split foot malformation locus SHFM1 at 7q21.3-q22.1 and analysis of a candidate gene for its expression during limb development.** *Hum Mol Genet* 1996, **5:**571-579.

11. Marchi E, Kanapin A, Magiorkinis G, Belshaw R: **Unfixed endogenous retroviral insertions in the human population.** *J Virol* 2014, **88:**9529-9537.
12. Ewing AD, Ballinger TJ, Earl D, Broad Institute Genome S, Analysis P, Platform, Harris CC, Ding L, Wilson RK, Haussler D: **Retrotransposition of gene transcripts leads to structural variation in mammalian genomes.** *Genome Biol* 2013, **14:**R22.
13. Tsuji J, Frith MC, Tomii K, Horton P: **Mammalian NUMT insertion is non-random.** *Nucleic Acids Res* 2012, **40:**9073-9088.
14. Ewing AD, Kazazian HH, Jr.: **High-throughput sequencing reveals extensive variation in human-specific L1 content in individual human genomes.** *Genome Res* 2010, **20:**1262-1270.
15. Jain M, Koren S, Miga KH, Quick J, Rand AC, Sasani TA, Tyson JR, Beggs AD, Dilthey AT, Fiddes IT, et al: **Nanopore sequencing and assembly of a human genome with ultra-long reads.** *Nat Biotechnol* 2018, **36:**338-345.
16. International HapMap C, Altshuler DM, Gibbs RA, Peltonen L, Altshuler DM, Gibbs RA, Peltonen L, Dermitzakis E, Schaffner SF, Yu F, et al: **Integrating common and rare genetic variation in diverse human populations.** *Nature* 2010, **467:**52-58.
17. Genomes Project C, Abecasis GR, Altshuler D, Auton A, Brooks LD, Durbin RM, Gibbs RA, Hurles ME, McVean GA: **A map of human genome variation from population-scale sequencing.** *Nature* 2010, **467:**1061-1073.
